# Bursa of Fabricius–independent B cells establish an IgA-mediated intestinal barrier that safeguards gut–liver homeostasis

**DOI:** 10.1073/pnas.2605569123

**Published:** 2026-07-15

**Authors:** Ryota Hirakawa, Motoshi Hisamatsu, Sayoko Maekawa, Eiki Asai, Miyuko Ohta, Ayumi Matsuo, Kunihiro Okano, Toh Miyazaki, Motofusa Akiyama, Masaaki Toyomizu, Jahidul Islam, Mutsumi Furukawa, Tomonori Nochi

**Affiliations:** ^a^International Education and Research Center for Food and Agricultural Immunology, Graduate School of Agricultural Science, Tohoku University, Miyagi 980-8572, Japan; ^b^ Laboratory of Animal Functional Morphology, Graduate School of Agricultural Science, Tohoku University, Miyagi 980-8572, Japan; ^c^ Laboratory of Animal Mucosal Immunology, Graduate School of Agricultural Science, Tohoku University, Miyagi 980-8572, Japan; ^d^GENODAS Inc.,Miyagi 980-0021, Japan; ^e^ Department of Biological Environment, Faculty of Bioresource Sciences, Akita Prefectural University, Akita 010-0195, Japan; ^f^Advanced Technology Development Center, Kyoritsu Seiyaku Corporation, Ibaraki 300-1252, Japan; ^g^ Laboratory of Animal Nutrition, Graduate School of Agricultural Science, Tohoku University, Miyagi 980-8572, Japan; ^h^ Division of Mucosal Vaccines, International Vaccine Design Center, The Institute of Medical Science, The University of Tokyo, Tokyo 108-8639, Japan; ^i^ Department of Animal Bioscience, University of Guelph, Ontario N1G 2W1, Canada; ^j^ School of Nutrition and Health Sciences, Taipei Medical University, Taipei City 11031, Taiwan; ^k^ Tohoku Center for Teaching and Learning, Institute for Excellence in Higher Education, Tohoku University, Miyagi 980-8576, Japan

**Keywords:** bursa of Fabricius, cecal tonsils, B cells, immunoglobulin A, host microbiome

## Abstract

The bursa of Fabricius (BF), a specialized lymphoid structure in birds, regulates avian B-cell development. However, the BF starts to regress posthatching, suggesting that as-yet-unidentified structures assume this function during maturation. This study reveals that BF-independent B-cell genesis involving the gut cecal tonsils (CTs) predominates over the BF-dependent pathway posthatching. Although B-cell progenitors originating from the bone marrow (BM) typically migrate to the BF, we identified a population that instead migrates to the CTs through CXCL12/CXCR4-mediated chemotaxis. These BF-independent CXCR4^+^ pre-B cells acquired surface IgM expression within the CT follicular region (FR) and differentiated into immunoglobulin A (IgA)-producing plasma cells. Inhibition of CXCR4^+^ cell influx from the BM impaired formation of the FR, altered the responsiveness of intestinal IgA to commensal bacteria, promoted gut dysbiosis, allowed translocation of pathogenic bacteria (e.g., *Streptococcus alactolyticus*) to the liver, and ultimately caused hepatic inflammation and metabolic dysfunction. These abnormalities were reversed by administering an IgA-enriched fecal preparation derived from healthy chickens. Collectively, these results reveal the existence of a population of BF-independent B cells that function in CTs. These cells represent a promising target for maintaining and improving the immunological and microbiological environment of the avian intestinal tract, which is closely linked to hepatic homeostasis.

## Significance

B cells were first identified in the bursa of Fabricius (BF), a lymphoid organ unique to birds. The BF has long been regarded as the central site of B-cell development, a concept that has remained largely unchanged since its discovery. Here, we identify a previously unrecognized population of BF-independent B cells in the cecal tonsils. Alongside conventional BF-dependent B cells, these BF-independent B cells contribute substantially to the production of intestinal immunoglobulin A (IgA), which predominantly recognizes beneficial microbiota. Furthermore, we demonstrate that intestinal IgA maintains microbial homeostasis, restricts the translocation of liver-colonizing pathogens, and preserves hepatic function. Collectively, our findings reveal a BF-independent pathway of B-cell development that maintains gut–liver homeostasis, thereby extending current understanding of avian immunology.

Avian immunology has played an integral role in advancing our understanding of the human immune system. The bursa of Fabricius (BF), a structure at the terminus of the digestive tract first described in the early 17th century, was later demonstrated to be the primary organ of avian hematopoiesis and shown by Glick (1956) to be indispensable for antibody production against pathogens (1). In fact, B cells, which differentiate into antibody-producing plasma cells (PCs), were first described in chickens (2–4). Although the BF is unique to birds, this discovery provided important insights into B-cell function in mammals and greatly advanced the field of immunology (5–7).

Historically, B-cell development in birds has been regarded as entirely BF-dependent. However, the BF reaches full development by around 1 mo posthatching and begins to regress approximately 50 to 100 d posthatching (8, 9), with near-complete regression by 5 to 7 mo of age. Moreover, involution of BF in adulthood has little effect on antibody production (10, 11), suggesting that B-cell development is supplanted by other tissues. The BF develops before hatching through the recruitment of B-cell precursors from the bone marrow (BM) via CXCL12- and CXCR4-mediated chemotaxis (12–14). These BF-derived B cells subsequently migrate to peripheral lymphoid tissues such as the spleen via CXCL13- and CXCR5-mediated chemotaxis, after which they commence antibody production in response to antigen recognition (15). Therefore, the CXCL12/CXCR4 and CXCL13/CXCR5 chemokine–chemokine receptor pathways are candidate mediators of BF-dependent and putative BF-independent B-cell development throughout the chicken lifespan.

Unlike mammals, birds lack lymph nodes but develop gut-associated lymphoid tissues (GALTs), such as the cecal tonsils (CTs) (16, 17). Although the spleen begins to form by recruiting B and T cells before hatching, the development of GALTs is markedly accelerated after hatching, and these structures acquire the capacity to synthesize antibodies, mainly of the immunoglobulin A (IgA) isotype (16, 18). GALT-derived IgA is secreted abundantly into the intestinal lumen, thereby influencing the development of the gut microbiome (19). Notably, certain bacterial species exhibit distinct functional phenotypes depending on whether they are IgA-coated or uncoated (20, 21). However, the molecular mechanisms underlying IgA synthesis in GALTs and their influence on gut function remain poorly understood.

In this study, we demonstrate BF-independent B-cell development in CTs concomitant with BF-dependent B-cell production. These BF-independent B cells also originate in the BM and migrate to the CTs via CXCL12–CXCR4 interactions while bypassing the BF. As chickens mature, BF-independent IgA-producing PCs predominate in gut antibody production. We further report that the absence of BF-independent IgA production permits translocation of pathogenic bacteria from the intestine, particularly to the liver, causing hepatic dysfunction and inflammation. These findings indicate that BF-independent B-cell genesis and intestinal IgA production in CTs help shape the gut microbiome and maintain host metabolic homeostasis throughout the lifespan of chickens.

## Results

### Identification and Characterization of BF-Independent B Cells that Differentiate into IgA PCs.

**Fig. 1.**
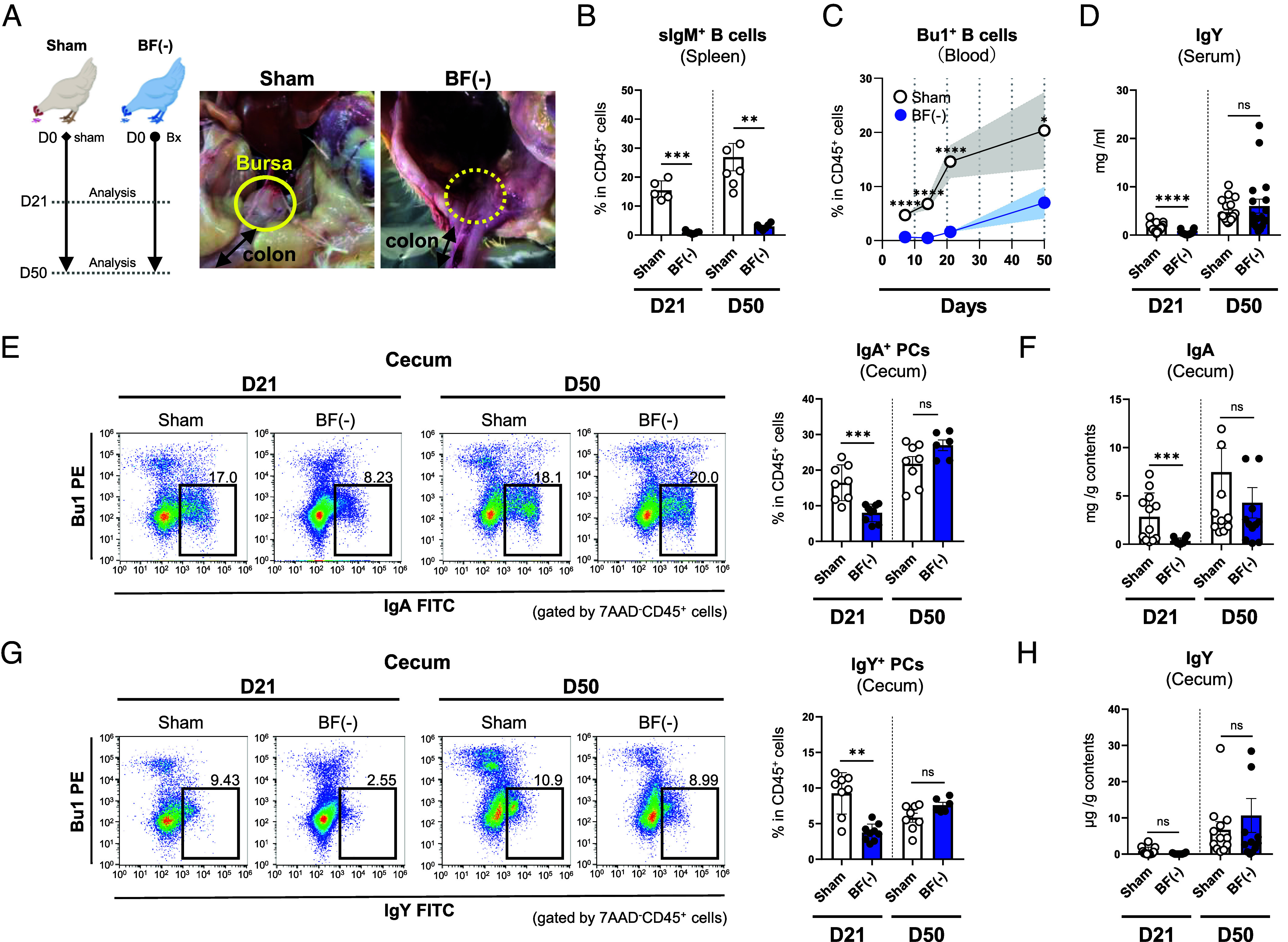
Identification of chicken B cells that differentiate into IgA-producing plasma cells (PCs) independently of the bursa of Fabricius (BF). (*A*) Accuracy of surgical BF removal [BF(−)] confirmed by visual inspection at day (D)21 and D50 posthatching. (*B*) Splenic Bu1^+^IgM^+^ B cells among CD45^+^ leukocytes at D21 and D50 (n = 5 to 9 per group and time point). (*C*) Bu1^+^ B cells in peripheral blood at D7, D14, D21, and D50 (n = 6 to 10 per group and time point). (*D*) Serum IgY levels at D21 and D50 (n = 18 to 30 per group and time point). (*E*) Abundant Bu1^−^IgA^+^ PCs in the cecum at D50 but not at D21 in the absence of BF (n = 6 to 9 per group and time point). (*F*) Comparable cecal IgA levels at D50 between sham-operated (Sham) and BF(−) chickens (n = 12 per group and time point). (*G*) Abundant Bu1^−^IgY^+^ PCs in the cecum at D50 but not D21 in the absence of the BF (n = 6 to 9 per group and time point). (*H*) Cecal IgY levels at D21 and D50 in Sham and BF(−) chickens (n = 12 per group and time point). Results obtained from samples collected from independent chickens are presented as mean ± SEM and were analyzed using a two-sided Mann–Whitney U test. ^∗^*P* < 0.05, ^∗∗^*P* < 0.01, ^∗∗∗^*P* < 0.001, ^∗∗∗∗^*P* < 0.0001. NS: not significant, D: day.

To investigate the involvement of the BF in intestinal immunity during chicken development and maturation, the entire BF was surgically removed at hatching, as described previously (2), and animals were raised until D21 or D50 for comparison of B cells and Ig release profiles with sham-operated (Sham) controls. Complete BF removal was confirmed at necropsy in the bursectomized group by visual inspection (Fig. 1*A*). Consistent with previous studies, IgM^+^ (Bu1^+^) B cells, identified by the pan-B cell marker Bu1, were undetectable in the spleen of bursectomized chickens at D21 and were scarcely detectable at D50 (Fig. 1*B*). Similarly, in peripheral blood, circulating Bu1^+^ B cells were undetectable up to D21 but became detectable by D50 (Fig. 1*C*). Serum IgY, the avian functional equivalent of IgG, was barely detectable at D21 but reached levels comparable to those of Sham chickens by D50 (Fig. 1*D*). The number of IgA PCs in the cecum and cecal IgA content were significantly lower in bursectomized chickens than in Sham controls at D21 but were comparable by D50 (Fig. 1 *E* and *F*). Cecal IgY PC level exhibited a similar trend; however, the amount of IgY secreted into the intestinal contents was extremely low compared with IgA (Fig. 1 *G* and *H*). Notably, oral immunization of bursectomized chickens with *Salmonella enterica* induced detectable levels of antigen-specific intestinal IgA, albeit slightly lower than those detected in Sham controls (*SI Appendix,* Fig. S1 *A and B*). This immunization failed to induce antigen-specific serum IgY, whereas serum IgY titers were effectively upregulated in Sham controls following immunization (*SI Appendix,* Fig. S1 *A and C*). Collectively, these results suggest the presence of a BF-independent B-cell development pathway that can partially compensate for early BF loss, resulting in the gradual establishment of intestinal immunity, including the generation of IgA PCs.

### Identification of B Cells at Distinct Stages of Differentiation in Gut CTs.

To characterize potential BF-independent B-cell development in the avian immune system, we first examined B- and T-cell distribution in multiple tissues outside the BF using immunostaining and flow cytometry. Lymphoid accumulation of B cells, predominantly Bu1^+^ B cells and T cells, mainly CD3^+^ T cells, was clearly detected in the spleen, CTs, ileal Peyer’s patches (PPs), cecum, and colon but not in the jejunum (*SI Appendix,* Fig. S2*A*). In CTs, PPs, cecum, and colon, Bu1^+^ B cells formed follicular regions (FRs) adjacent to interfollicular regions (IFRs) containing CD3^+^ T cells and Bu1^+^ B cells. Flow cytometry revealed at least three distinct B-cell populations, Bu1^high^ surface IgM(sIgM)^−^, Bu1^low^ sIgM^low^ and Bu1^low^ sIgM^high^, corresponding to different stages of B-cell differentiation from pre-B cells to sIgM^+^ B cells (*SI Appendix,* Fig. S2*B*). Abundant pre-B cells were observed in the BF, whereas more numerous sIgM^+^ B cells were found in the spleen (*SI Appendix,* Fig. S2*B*). Pre-B cells and sIgM^+^ B cells were clearly observed in CTs and PPs (*SI Appendix,* Fig. S2*B*). The distribution of B cells within CTs was strongly dependent on differentiation stage, with pre-B cells predominantly detected in marginal areas of FRs and most sIgM^+^ B cells localized within IFRs (*SI Appendix,* Fig. S2 *C* and *D*). These results indicate that CTs function as primary lymphoid tissues containing differentiating B cells and secondary lymphoid tissues containing mature B cells capable of pathogen detection.

**Fig. 2.**
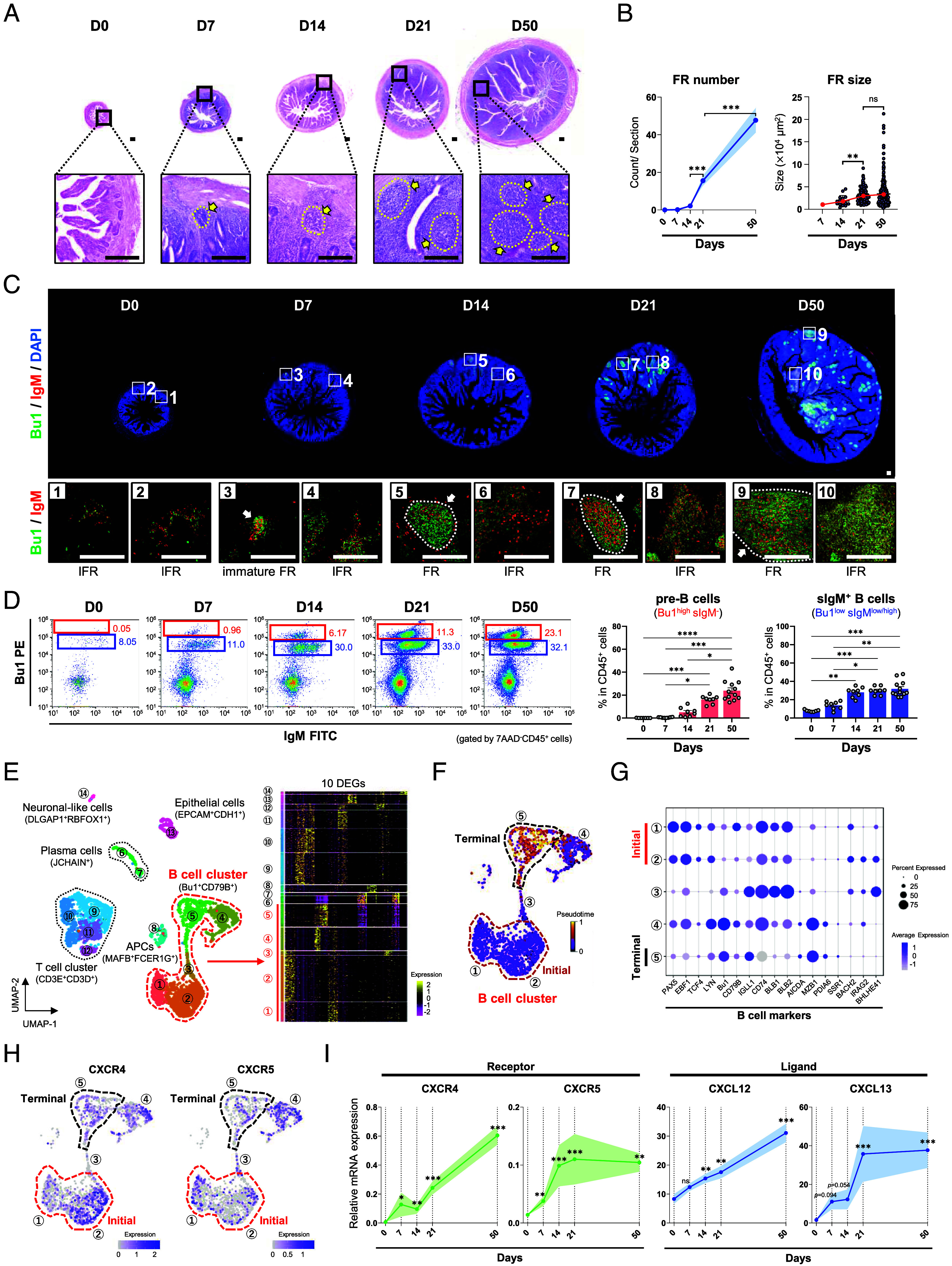
Identification of B cells at distinct stages of differentiation within gut cecal tonsils (CTs). (*A*) Representative hematoxylin and eosin images of CTs at D0, D7, D14, D21, and D50. Yellow arrows: follicular regions (FRs). (*B*) Increased number and size of FRs with age (n = 6 to 7 per time point). (*C*) Immunofluorescence images showing Bu1^+^ (green) and IgM^+^ (red) cells in CTs at the indicated ages. High magnification: FRs and interfollicular (IFR) regions. (Scale bar, 200 μm.) (*D*) Frequencies of pre-B and surface IgM (sIgM)^+^ B cells in CTs based on Bu1 and sIgM expression (n = 7 to 12 per time point). (*E*) Heatmap of DEGs and uniform manifold approximation and projection (UMAP) of 3,472 cells isolated from CTs at D50. (*F*) RNA velocity–based pseudotime analysis using dynamical modeling. (*G*) Genes expressed in cell clusters C1 to C5, confirming their identity as B-cell lineages. (*H*) Scatterplots of CXCR4 and CXCR5 expression across B-cell clusters. (*I*) mRNA expression levels of *cxcr4* and *cxcr5* as well as their respective ligands *cxcl12* and *cxcl13* in CTs at D0, D7, D14, D21, and D50 (n = 6 to 8 per time point). Results obtained from samples collected from independent chickens are presented as mean ± SEM and were analyzed using either the Kruskal–Wallis test followed by Dunn’s multiple comparison test or a two-sided Mann–Whitney U test. ^∗^*P* < 0.05, ^∗∗^*P* < 0.01, ^∗∗∗^*P* < 0.001, ^∗∗∗∗^*P* < 0.0001. NS: not significant, D: day. (Scale bar, 200 μm.)

CTs were not detected at hatching (D0), but organogenesis was initiated around D7, and CTs became prominent by D21, when FRs enlarged through the expansion of sIgM^+^ B cells and pre-B cells (Fig. 2 *A*–*C*). Flow cytometry further revealed cell type–dependent recruitment to CTs, with sIgM^+^ B cells appearing by D7 and pre-B cells emerging around D14 (Fig. 2*D*). Single-cell RNA sequencing (scRNA-seq) identified 14 distinct cell clusters in CTs, including five B-cell clusters [clusters 1 to 5 (C1 to C5)] (Fig. 2*E*, *SI Appendix,* Fig. S3*A*, and Dataset S1). Notably, clusters C1 and C2 highly expressed *pax5* and *ebf1*, master regulators of B-cell lineage commitment (22), and these two clusters represented the earliest stage of B-cell differentiation based on RNA velocity analyses (Fig. 2 *F* and *G* and *SI Appendix,* Fig. S3 *B* and *C*). Cells in clusters C1 and C2 also abundantly expressed CXCR4 but not CXCR5 (Fig. 2*H*). CXCR4^+^ cells were predominantly localized within FRs, wherein the CXCR4 ligand CXCL12 was also frequently detected (*SI Appendix,* Fig. S3 *D*–*J*). The expression levels of CXCR4 and CXCL12 increased progressively in FRs after hatching, whereas CXCR5 and CXCL13 expression levels peaked by D21 (Fig. 2*I*). Together, these results suggest that sustained pre-B-cell accumulation in CTs is primarily driven by CXCR4-mediated chemotaxis.

### Identification of BF-Independent CXCR4^+^ Pre-B Cells in CTs.

**Fig. 3.**
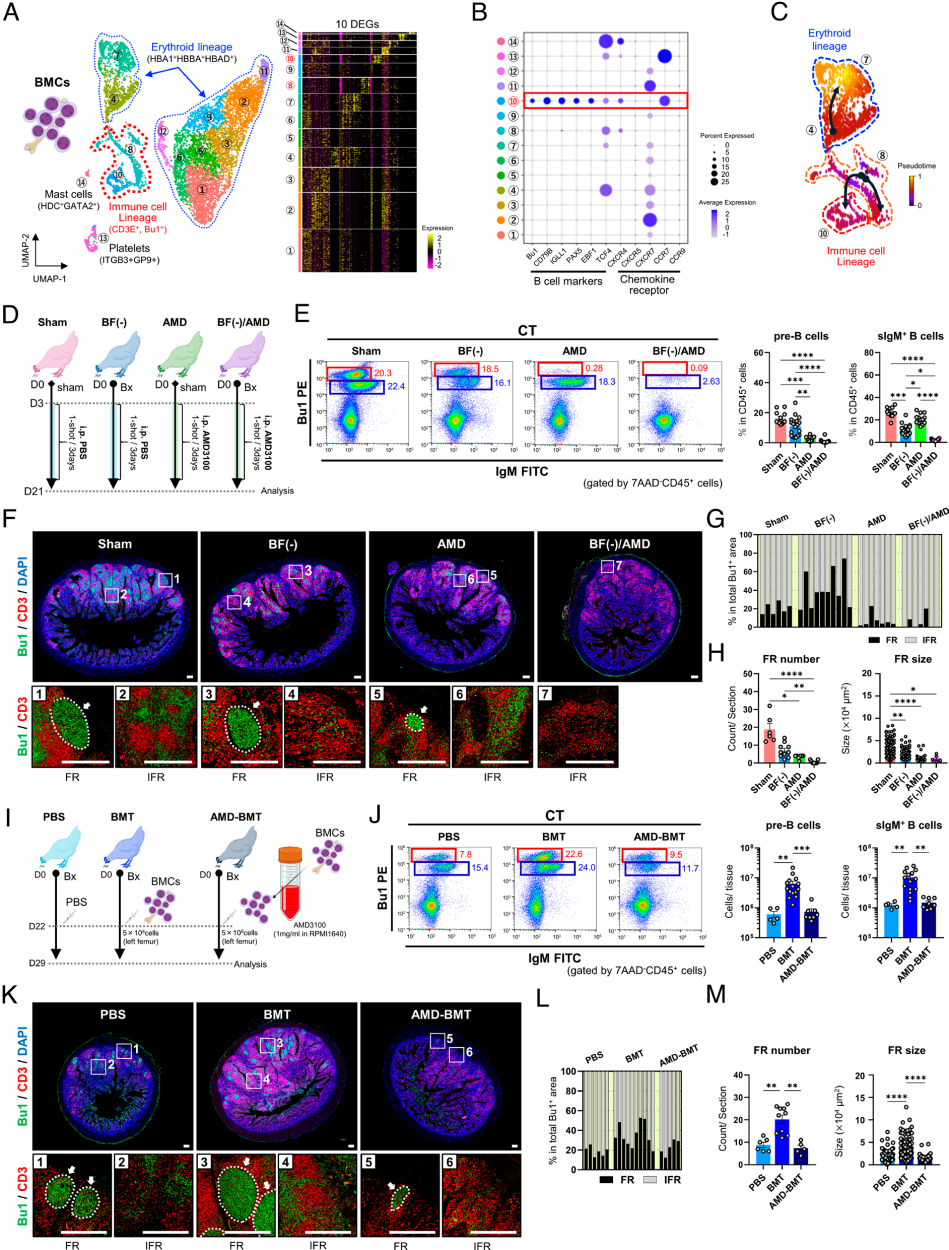
Demonstration of BF-independent pre-B cells in CTs. (*A*) Heatmap of DEGs and UMAP showing gene expression profiles of 7,481 cells isolated from the bone marrow (BM) at D50. (*B*) Genes expressed in cell cluster C10, confirming their identity as lymphoid progenitors (including B-cell precursors). (*C*) RNA velocity–based pseudotime analysis using dynamical modeling. (*D*) Four animal groups: Sham, BF removal [BF(−)], AMD3100 treatment (AMD) and combined treatment [BF(−)/AMD]. (*E*) Frequencies of pre-B cells and sIgM^+^ B cells (Bu1^low^IgM^low^ and Bu1^low^IgM^high^) in CTs (n = 12 to 19 per group). (*F*) Immunofluorescence images of CTs containing Bu1^+^ B cells (green) and CD3^+^ T cells (red). (*G*) Frequencies of FRs and IFRs in the total Bu1^+^ B-cell area of CTs. (*H*) Number and size of FRs in CTs (n = 6 to 10 per group). (*I*) Three animal groups: bursectomize BF(−) chickens transplanted with PBS, BM cells (BMT), or BM cells pretreated with AMD3100 (AMD-BMT). (*J*) Cell number of pre-B cells and sIgM^+^ B cells in CTs (n = 5 to 15 per group). (*K*) Immunofluorescence images of CTs revealing Bu1^+^ B cells (green) and CD3^+^ T cells (red). (*L*) Frequencies of FRs and IFRs in the total Bu1^+^ B-cell area of CTs. (*M*) Number and size of FRs in CTs (n = 5 to 10 per group). Results obtained from samples collected from independent chickens are presented as mean ± SEM and were analyzed by the Kruskal–Wallis test followed by Dunn’s multiple comparisons test. ^∗^*P* < 0.05, ^∗∗^*P* < 0.01, ^∗∗∗^*P* < 0.001, ^∗∗∗∗^*P* < 0.0001. NS: not significant, D: day, Bx: BF removal. (Scale bar, 200 μm.)

We next examined the origins of pre-B cells in CTs and their potential dependence on the BF. Subsequent scRNA-seq of BM identified 14 distinct cell clusters, among which a subset within one population (C10) specifically expressed *pax5* and *ebf1*, along with *bu1* and *cd79b*, which encode proteins forming a complex with the B-cell receptor (Fig. 3 *A* and *B*, *SI Appendix,* Fig. S4*A*, and Dataset S2). The C10 population appeared to comprise multiple lymphoid progenitor types undergoing differentiation and included a subset exhibiting high CXCR4 expression but no detectable CXCR5 expression (Fig. 3 *B* and *C*, *SI Appendix,* Fig. S4 *B*–*F*, and Dataset S3). Given the tropism of BM-derived cells toward CXCL12 (*SI Appendix,* Fig. S4*G*), these CXCR4^+^ B-cell precursors may gradually infiltrate CTs after hatching without first passing through the BF. B-cell receptor repertoire analyses indicate that these cells already possess a certain degree of immunoglobulin diversity, suggesting that they are already engaged in B-cell differentiation in CTs (*SI Appendix*, Fig. S5 *A*–*E*). To verify BF-independent B-cell development in CTs, CXCL12/CXCR4-mediated chemotaxis was inhibited by treating chickens with the CXCR4 antagonist AMD3100 (12) in combination with BF removal (Fig. 3*D*), and B-cell migration profiles were examined. Treatment with AMD3100 suppressed the migration of pre-B cells into CTs, resulting in abnormal FR development and a reduced number of sIgM^+^ B cells within the tissue (Fig. 3 *E*–*H*). In vivo administration of aCXCL12 neutralizing antibodies (*SI Appendix,* Fig. S6 *A*–*D*) also suppressed the migration of pre-B cells into CTs. Notably, the number of sIgM^+^ B cells (but not pre-B cells) in IFR of CTs was markedly reduced at D21 following early BF removal (Fig. 3 *E*–*H*), consistent with a significant loss of sIgM^+^ cells in the spleen and disappearance of germinal centers (*SI Appendix,* Fig. S7 *A*–*D*). However, by D50, sIgM^+^ B cells developed sufficiently in CTs even in the absence of the BF, suggesting differentiation from BF-independent pre-B cells (*SI Appendix,* Fig. S8 *A*–*D*).

To characterize the migration pattern of BM-derived CXCR4^+^ B-cell precursors into CTs independently of the BF, an adoptive transfer experiment was conducted in which BF-deficient chickens received BM-derived cells from healthy donors (Fig. 3*I*). Consistent with enhanced migration into CTs in the absence of the BF, adoptive transfer of BM-derived cells increased the numbers of pre-B cells and sIgM^+^ B cells in CTs, as well as the number and size of FRs. These increases were markedly suppressed when donor BM-derived cells were pretreated with AMD3100 (Fig. 3 *J*–*M*), confirming dependence on CXCL12/CXCR4-mediated chemotaxis. It should be noted that the numbers of pre-B cells and sIgM^+^ cells in CTs were also markedly reduced following thymectomy (*SI Appendix,* Fig. S9 *A*–*F*). Adoptive transfer of thymus-derived CD4^+^ or CD8^+^ T cells rescued CT development in thymectomized chickens (*SI Appendix,* Fig. S9 *E*–*I*), consistent with the presence of a T-cell subset in CTs expressing CXCL12 (*SI Appendix,* Fig. S9 *J*–*L*). CXCL12 expression in CD4^+^ and CD8^+^ T cells isolated from CTs was confirmed by qRT-PCR, supporting the conclusion that these T-cell populations represent an important local source of CXCL12 (*SI Appendix,* Fig. S9*J*). These results indicate that BM-derived CXCR4^+^ B-cell precursors, in cooperation with thymus-derived CXCL12-expressing cells, migrate directly into CTs via CXCL12/CXCR4-mediated chemotaxis.

### Predominance of BF-Independent Pre-B Cells in Shaping Intestinal Immunity and the Microbiota.

**Fig. 4.**
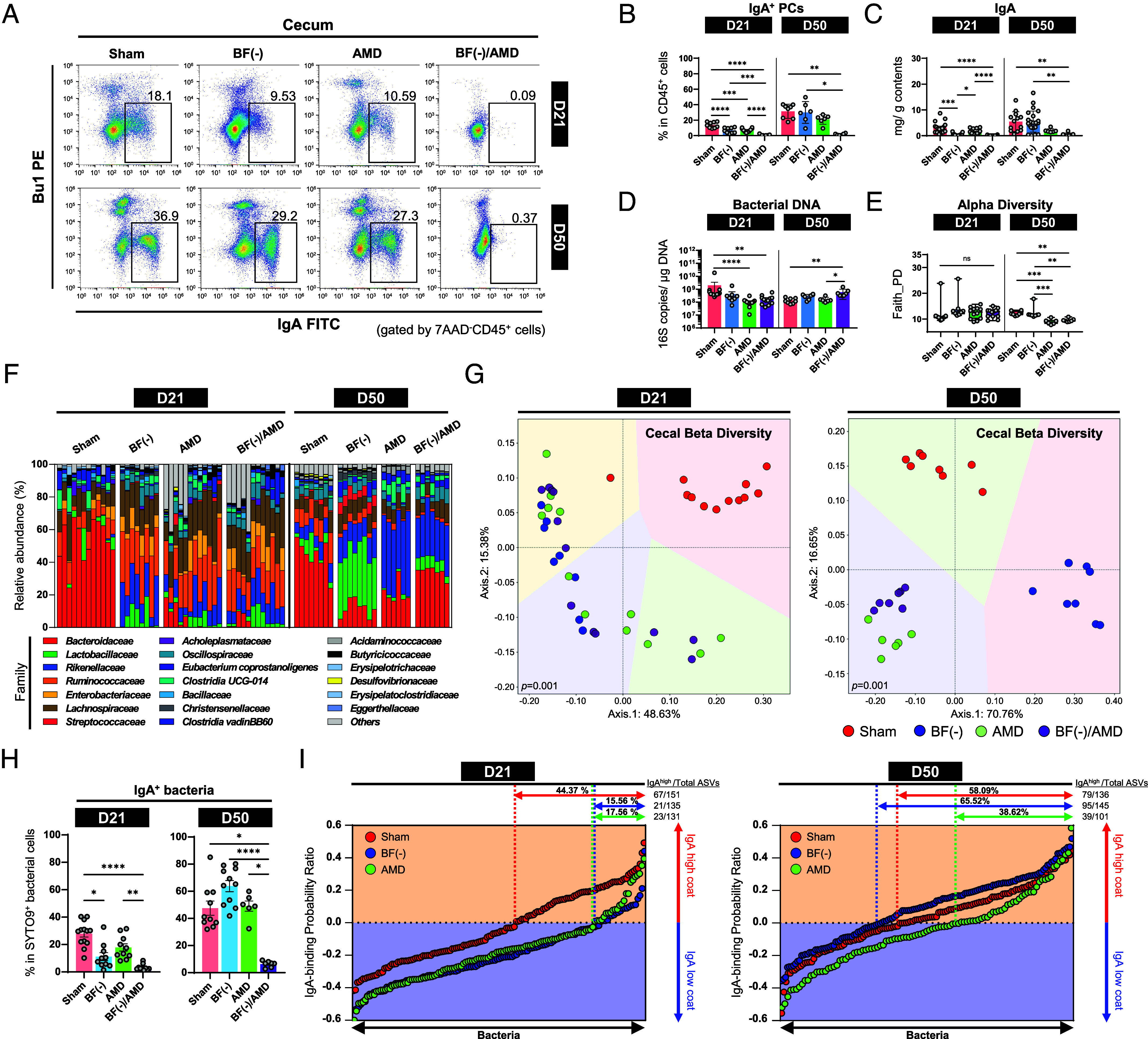
Shaping of cecal microbiome structure by BF-independent B cell–derived IgA. (*A* and *B*) Frequencies of Bu1^−^IgA^+^ plasma cells in the cecum at D21 and D50 (n = 6 to 10 per group and time point). (*C*) Cecal IgA levels at D21 and D50 (n = 6 to 23 per group and time point). (*D*) Copy number of 16S rRNA in cecal contents (n = 6 per group and time point). (*E*) Phylogenetic diversity of the cecal microbiome (n = 6 to 12 per group and time point). (*F*) Family-level bacteria composition of the cecal microbiome. (*G*) PCoA of weighted UniFrac distances (n = 6 to 12 per group and time point). Two-dimensional sample classification was conducted using the k-means clustering algorithm. (*H*) Frequencies of SIgA-coated microbes at D21 and D50 (n = 6 to 13 per group and time point). (*I*) IgA-binding probability ratio of bacteria (ASV) calculated using IgAScores (n = 6 to 8 per group and time point). Results obtained from samples collected from independent chickens are presented as mean ± SEM and were analyzed by the Kruskal–Wallis test followed by Dunn’s multiple comparisons test. ^∗^*P* < 0.05, ^∗∗^*P* < 0.01, ^∗∗∗^*P* < 0.001, ^∗∗∗∗^*P* < 0.0001. NS: not significant, D: day.

To examine the relative contributions of BF-dependent and -independent B cells to immune development and function in the gastrointestinal tract, the number of IgA^+^ PCs and the amount of IgA in the cecum were compared following BF removal and/or suppression of CT development using AMD3100 treatment. Production of IgA was reduced at D21 in chickens receiving bursectomy and/or AMD3100 treatment posthatching compared with Sham or vehicle-treated controls (Fig. 4 *A*–*C*), but IgA levels became comparable to controls by D50. In contrast, IgA remained nearly undetectable in chickens receiving both BF removal and AMD3100 treatment (Fig. 4 *A*–*C*). Notably, IgA^+^ PCs increased following adoptive transfer of BM-derived cells into BF-deficient chickens (*SI Appendix,* Fig. S10 *A*–*D*). These findings indicate that both BF-dependent and -independent B cells cooperatively contribute to IgA production.

Microbiome analysis revealed only minor differences in total bacterial numbers among the groups (Fig. 4*D*), but α-diversity was significantly reduced in chickens treated with AMD3100, particularly at D50, regardless of BF removal or Sham control (Fig. 4*E*). Furthermore, gut microbiome and the metabolic functions were significantly changed after either BF removal or AMD3100 treatment at both D21 and D50. Notably, AMD3100 treatment had a more pronounced effect than BF removal at D50 (Fig. 4 *F* and *G* and *SI Appendix,* Fig. S11 *A*–*C*). To investigate the microbial target specificity of BF independent B cells-derived IgA, IgA-coated bacteria in chickens treated with either BF removal or AMD3100 were analyzed by the FACS-based IgA-SEQ approach. The proportion and diversity of IgA-coated bacteria in the cecum was reduced at D21 following BF removal, but this reduction did not persist until D50 (Fig. 4*H* and *SI Appendix,* Fig. S12 *A*–*E*). Instead, most gut bacteria in chickens subjected to BF removal were adequately coated with IgA at D50 (Fig. 4*I*), with IgA-binding targets similar to those observed in Sham chickens (*SI Appendix,* Fig. S13 *A* and *B*). Furthermore, a strong association between IgA-coated and IgA-uncoated bacterial populations emerged only at D50, suggesting that the influence of BF-independent IgA on the cecal microbiota increases over the chicken lifespan (*SI Appendix,* Fig. S12*E*). Among IgA-coated bacteria, members of the family *Lachnospiraceae*, which are known producers of short-chain fatty acids (SCFAs) (23), were frequently detected in both Sham and bursectomized chickens but were scarce in AMD3100-treated chickens, particularly at D50 (*SI Appendix,* Fig. S13 *C* and *D*). These results suggest that BM-derived pre-B cells gradually assume the role of BF-derived sIgM^+^ B cells in CTs, with their contribution becoming progressively more important in shaping the intestinal microbiome over time, including the generation of IgA responses targeting beneficial bacterial taxa such as *Lachnospiraceae*.

### Impact of BF-Independent Immunity on Hepatic Metabolic and Immune Functions.

**Fig. 5.**
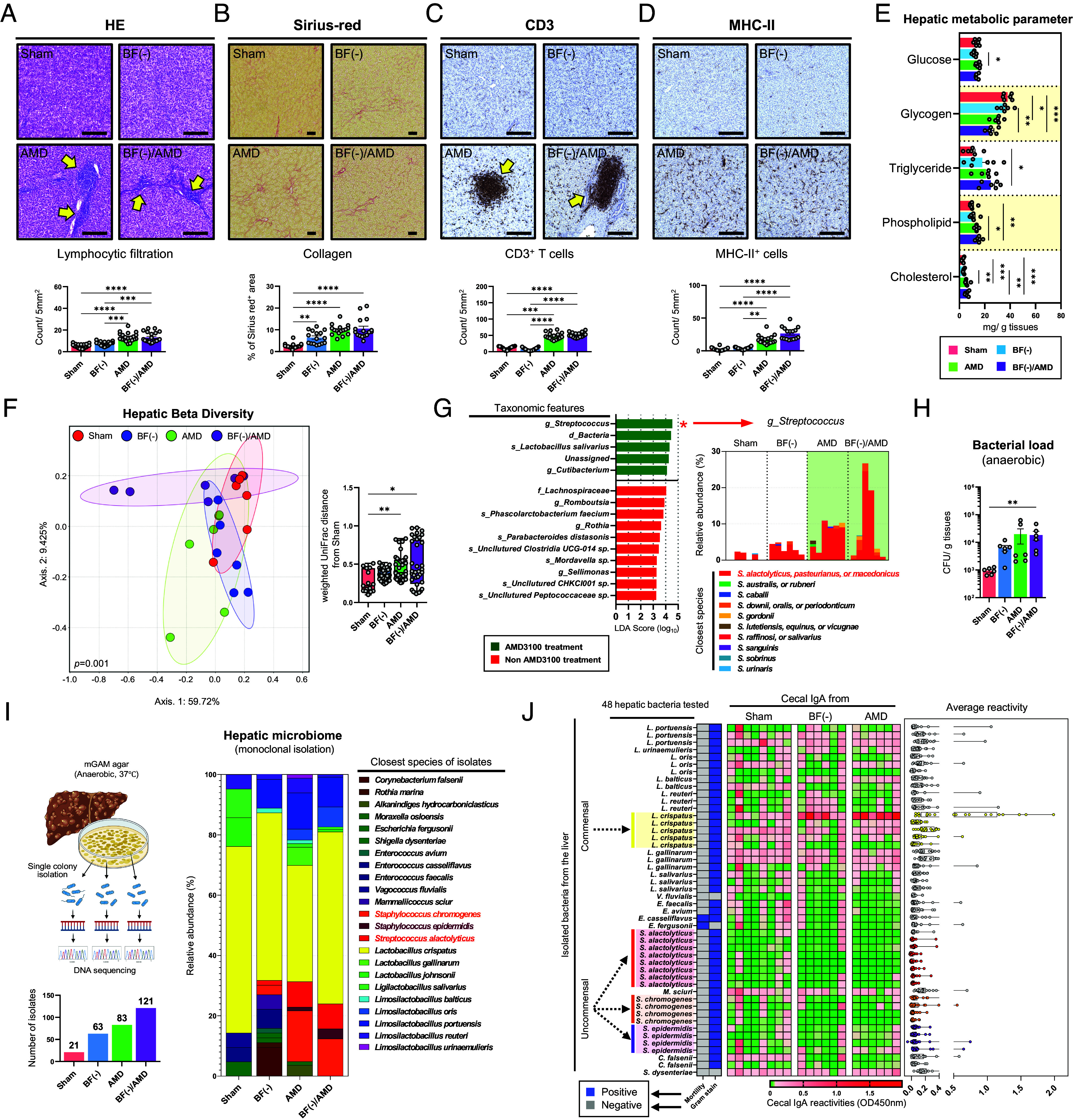
Intestinal immune impairment induced by disruption of CT development promoted hepatic inflammation, metabolic dysfunction, and pathogenic bacteria infiltration. (*A*) H&E-stained images showing lymphocytic filtration (yellow arrows) in liver tissue of D50 chickens treated with AMD3100 to disrupt CT development with or without BF removal (n = 5 per group). (*B*) Sirius red-stained images showing the development of collagen fibers in the liver of AMD3100-treated D50 chickens (n = 4 per group). (*C* and *D*) Immunohistochemical staining showing CD3^+^ T cells (*C*) and MHC-II^+^ antigen-presenting cells (*D*) in the liver of AMD3100-treated D50 chickens (n = 4 per group). (*E*) Altered hepatic metabolic parameters at D50 (n = 6 per group). (*F*) PCoA of weighted UniFrac distances characterizing the hepatic microbiome (n = 6 per group). (*G*) LEfSe analysis revealing enriched taxa in the liver of AMD3100-treated chickens. Species-level classifications in *Streptococcus* were performed with BLAST algorithm. (*H*) Bacterial load in liver samples cultured under anaerobic conditions. (*I*) Species-level abundance of 286 active isolates from the liver (n = 3 per group). (*J*) Reactivity of intestinal IgA against 48 hepatic isolates representing 18 species (n = 6 to 8 per group). Results obtained from samples collected from independent chickens are presented as mean ± SEM and were analyzed by the Kruskal–Wallis test followed by Dunn’s multiple comparisons test. ^∗^*P* < 0.05, ^∗∗^*P* < 0.01, ^∗∗∗^*P* < 0.001, ^∗∗∗∗^*P* < 0.0001. NS: not significant. (Scale bar, 100 μm.)

Hepatic tissue abnormalities were detected in chickens treated with AMD3100, with or without BF removal. These abnormalities included enhanced eosin staining of hepatocytes, sinusoidal hypertrophy, excessive collagen deposition and leukocyte accumulation (Fig. 5 *A* and *B*), suggesting that BF-independent B-cell function within CTs is an essential component of the gut–liver axis that maintains nutritional and metabolic homeostasis. Moreover, AMD3100-treated chickens exhibited increased numbers of MHC class II–presenting cells scattered throughout the liver, along with accumulation of CD3^+^ T cells in Glisson’s capsule and liver lobules (Fig. 5 *C* and *D*). Hepatic levels of cholesterol, phospholipid, and triglyceride were increased, whereas glycogen content was reduced following AMD3100 treatment (Fig. 5*E*), indicating substantial metabolic dysregulation. These findings were further supported by bulk RNA sequencing (RNA-seq), which revealed upregulation of multiple genes involved in immune responses (e.g., inflammation, phagocytosis) and lipid metabolism (*SI Appendix,* Fig. S14 *A*–*E*). These gene expression changes were associated with altered hepatic markers of liver function in AMD3100-treated chickens lacking the BF at both D21 and more markedly at D50 (*SI Appendix,* Fig. S14 *A*–*E* and Datasets S4–S8).

To address the mechanism underlying the hepatic abnormalities induced by AMD3100 treatment, we next examined the hepatic microbiome. The increased levels of bacterial DNA detected in plasma suggest that B-cell depletion promotes bacterial translocation into the circulation (*SI Appendix,* Fig. S15*A*). Multiple microbial detection assays demonstrated that the avian liver harbors a microbiome, and that B-cell depletion in CTs increased hepatic bacterial burden (*SI Appendix,* Fig. S15 *A*–*D*). AMD3100 treatment altered the hepatic bacterial community compared with untreated controls (Fig. 5*F*). Particularly, g_*Streptococcus*, *g_Cutibacterium,* and s_*Lactobacillus salivarius* were abundantly detected in the liver following AMD3100 treatment (Fig. 5*G*). Many of these hepatic bacteria, including pathogenic bacteria such as *Streptococcus alactolyticus, Staphylococcus epidermidis,* and *Staphylococcus chromogenes*, could be isolated and cultured under anaerobic but not aerobic conditions (Fig. 5 *H* and *I*, *SI Appendix*, Fig. S15 *B*, *E*, and *F*, and Datasets S9 and S10). In contrast, beneficial bacteria (e.g., *Lactobacillus crispatus*) were detected regardless of treatment (Fig. 5*I* and *SI Appendix,* Fig. S15*F*). Whole-genome sequencing of selected bacterial isolates unique to the AMD3100-treated group revealed a *S. alactolyticus* strain expressing the extracellular glycogen-degrading enzyme pullulanase, as well as several virulence factors associated with immune evasion (*SI Appendix,* Fig. S15 *G*–*I* and Datasets S11–S14). Notably, most pathogenic bacterial isolates detected in the liver following AMD3100 treatment were not coated by intestinal IgA, whereas beneficial bacterial isolates were mostly IgA-coated (Fig. 5*J* and *SI Appendix,* Fig. S16 *A* and *B*). These findings indicate that CT-derived IgA shapes the gut microbiome and contributes to the maintenance of hepatic homeostasis in chickens (*SI Appendix,* Fig. S16 *C* and *D* and Dataset S15). These results further suggest that loss of CT function by AMD3100 treatment rather than BF loss abolishes IgA recognition of beneficial bacteria and the capacity to prevent translocation of selected IgA-uncoated pathogenic bacteria to the liver, despite preserved and even enhanced immune activation.

### Reversal of Hepatic Abnormalities Following Administration of IgA-Enriched Preparations.

**Fig. 6.**
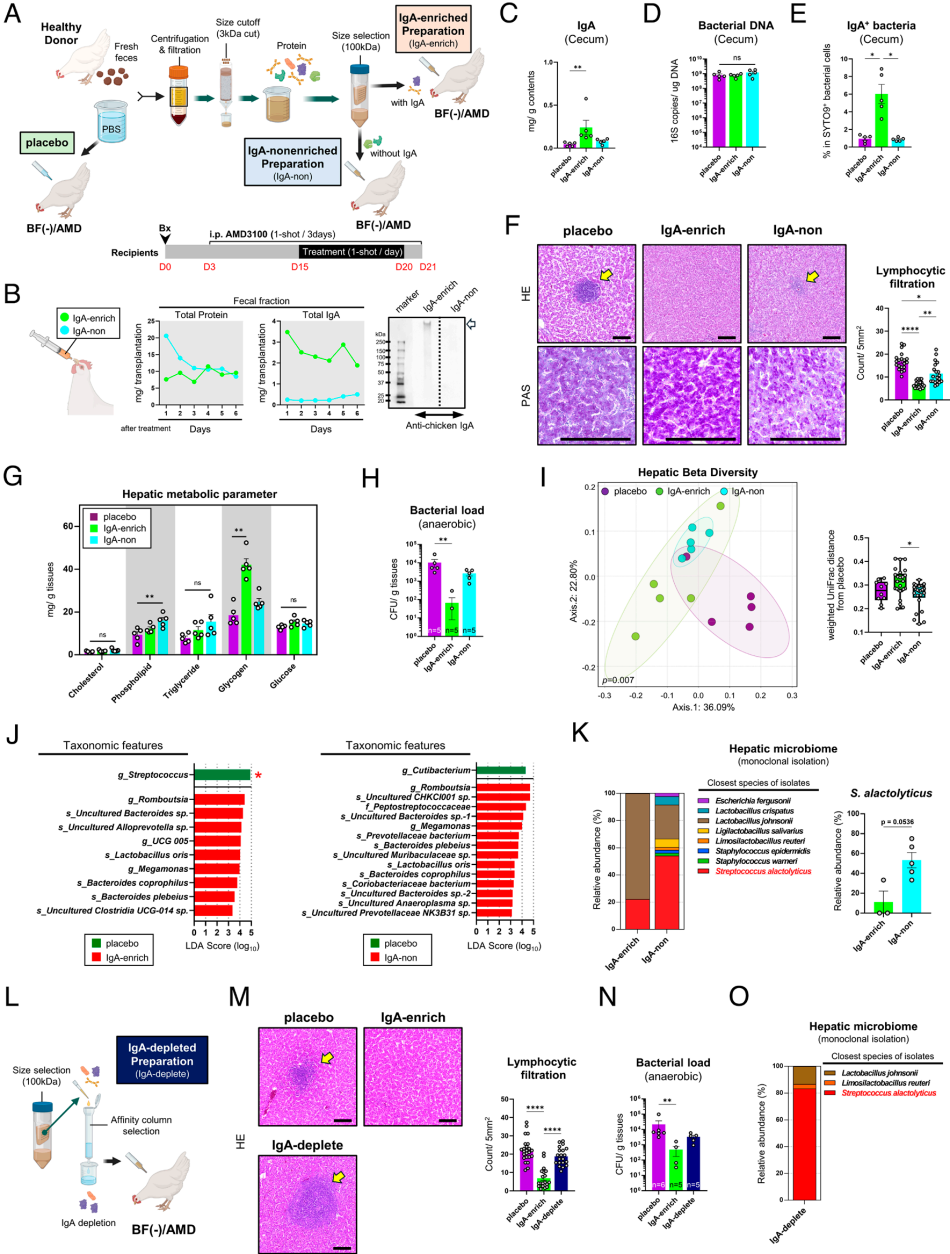
Restoration of hepatic function by transplantation with an IgA-enriched fecal preparation. (*A* and *B*) Three animal groups: untreated BF(−)/AMD chickens, BF(−)/AMD chickens treated with an IgA-enriched fecal preparation (IgA-enrich) and BF(−)/AMD chickens treated with an IgA-non-enriched fecal preparation (IgA-non). (*C*) Total IgA concentrations in cecal contents (n = 5 per group). (*D*) 16S rRNA copy number in cecal contents (n = 5 per group). (*E*) Frequencies of IgA-coated microbes after transplantation (n = 5 per group). (*F*) H&E-stained images showing lymphocytic filtration (yellow arrows) in liver tissue after transplantation (n = 5 per group). (*G*) Hepatic metabolic parameters (n = 5 per group). (*H*) Bacterial load in liver samples cultured under anaerobic conditions (n = 5 per group). (*I*) Hepatic microbiome and PCoA of weighted UniFrac distances (n = 5 per group). (*J*) LEfSe presenting enriched taxa in the liver. (*K*) Relative abundance of 63 active isolates and *S. alactolyticus* in the liver (n = 3 to 5 per group). (*L*) Three animal groups: untreated BF(−)/AMD chickens, BF(−)/AMD chickens treated with an IgA-enriched fecal preparation (IgA-enrich) and BF(−)/AMD chickens treated with an IgA-depleted fecal preparation (IgA-deplete). (*M*) H&E-stained images showing lymphocytic filtration (yellow arrows) in liver tissue after transplantation (n = 5 to 6 per group). (*N*) Bacterial load in liver samples cultured under anaerobic conditions (n = 5 to 6 per group). (*O*) Relative abundance of 60 active isolates and *S. alactolyticus* in the liver of BF(−)/AMD chickens treated with an IgA-depleted fecal preparation (n = 5). Results obtained from samples collected from independent chickens are presented as mean ± SEM and were analyzed using either the Kruskal–Wallis test followed by Dunn’s multiple comparison test or a two-sided Mann–Whitney U test. ^∗^*P* < 0.05, ^∗∗^*P* < 0.01, ^∗∗∗∗^*P* < 0.0001. NS: not significant, D: day. (Scale bar, 100 μm.)

To further assess the importance of intestinal IgA production in regulating hepatic function and preventing dysfunction, IgA-deficient chickens were administered IgA-enriched fecal preparations (or IgA-depleted fecal preparation as a control) derived from healthy chickens. Briefly, fecal protein fractions with or without IgA were prepared from fecal samples of healthy chickens by removing bacteria and metabolites, followed by size-based fractionation into <100- and >100-kDa fraction (Fig. 6*A*). The IgA content comprised 20 to 46% of total protein in the >100-kDa fraction but only 1 to 5% in the <100-kDa fraction (Fig. 6*B*). Chickens deficient in IgA and receiving the >100-kDa fraction showed increased cecal IgA levels, a higher frequency of IgA-coated bacteria, shifts in the cecal microbiota, and distinct microbial compositions between IgA-coated and -uncoated bacteria (Fig. 6 *C*–*E* and *SI Appendix*, Fig. S17 *A*–*E*). In contrast, administration of the <100-kDa fraction failed to restore these parameters. Importantly, consistent with our previous results (Fig. 5*J*), the IgA-binding score of *Streptococcus* remained very low even after administration of the >100-kDa fraction, while the members of f_*Lachnospiraceae* and g_*Eubacterium coprostanoligenes* were preferentially coated by transplanted IgA in the recipients (*SI Appendix,* Fig. S17 *F* and *G*). Furthermore, administration of the IgA-enriched preparation reduced hepatic inflammation, enhanced hepatic glycogen content, reduced serum triglyceride levels and inhibited gut-to-liver translocation of pathogenic bacteria (Fig. 6 *F*–*J* and *SI Appendix,* Fig. S18 *A*–*C*). For instance, pathogenic *S. alactolyticus* was rarely detected in the livers of IgA-deficient chickens administered the IgA-enriched preparation (>100 kDa) but was abundant in liver samples from IgA-deficient chickens administered the <100 kDa preparation (Fig. 6*K*, *SI Appendix,* Fig. S18*D*, and Dataset S16). Of note, the relative abundance of *Streptococcus* in the cecum was not significantly altered by any treatment (*SI Appendix,* Fig. S17*C*). To finally assess whether the observed hepatic recovery was attributable to intestinal IgA, an IgA-depleted >100-kDa fraction was prepared by removing IgA using affinity chromatography with anti-chicken IgA antibodies (Fig. 6*L* *and* *SI Appendix,* Fig. S19*A*). Transplantation of the IgA-depleted >100-kDa fraction, in which residual IgA accounted for only 4.1 to 19.1% of the total protein, failed to ameliorate the associated hepatic abnormalities or restore bacterial IgA coating in the cecum (Fig. 6 *M*–*O*, *SI Appendix,* Fig. S19 *A*–*C*, and Dataset S17). Taken together, these results indicate that the development of the cecal microbiota depends on intestinal IgA, which is generated by newly identified BF-independent B cells in collaboration with BF-derived B cells. Disruption of IgA-mediated intestinal immunity promotes the rapid influx of IgA-noncoated pathogenic bacteria (such as *S. alactolyticus*) from the gut into the liver, leading to hepatic metabolic and immune abnormalities.

## Discussion

The present study provides the evidence for a BF-independent B-cell differentiation pathway and its functional significance in supporting intestinal IgA production, shaping the gut microbiome and preventing translocation of pathogenic gut bacteria to the liver. Unique aspects of this study include characterization of avian immune system development, particularly intestinal immunity, in the absence of the BF, as well as a long-term observation period (up to 50 d) following bursectomy. This design allowed us to demonstrate that the BF-independent B-cell differentiation pathway predominates during posthatching maturation. BF-dependent and -independent B-cell populations cooperatively supported intestinal IgA immunity while exhibiting distinct bacterial recognition profiles, indicating functional differences in antigen specificity. Furthermore, our findings imply the existence of a transitional phase in which BF-dependent immunity remains significant while BF-independent B-cell responses expand progressively. Another central finding of this study is B-cell differentiation in the BM independent of the BF. Although it has been widely accepted that B cells develop in the BF from BM progenitor cells, our findings clearly demonstrate that BM-derived B-cell precursors can migrate directly into GALTs, such as CTs, where they mature into fully functional B cells.

These findings do not refute or diminish the importance of BF-derived B cells; rather, they suggest that BF-dependent and BF-independent B cells play complementary roles in coordinating immune development during avian maturation. BF-derived B cells begin migrating into CTs shortly after hatching, followed by the direct migration of BM-derived CXCR4^+^ B-cell precursors (Fig. 2 *A*–*D*). The BF-dependent pathway is mediated by CXCL12, which is produced primarily by stromal cells in the BF anlage during early morphogenesis (embryonic days 11 to 14) and by pre-B cells within BF follicles after hatching (13, 14). As chickens mature, however, the BF gradually regresses, and its immunological functions are gradually assumed by the BM and CTs, particularly the continuous generation of B-cell precursors and their differentiation into pre-B cells and sIgM^+^ B cells (*SI Appendix*, Fig. S8 *A*–*D*). Within CTs, stromal cells and sparsely distributed CD45^+^ immune cells, including CD4^+^ and CD8^+^ T cells, produce high levels of CXCL12, thereby facilitating the infiltration of BM-derived CXCR4^+^ B-cell precursors (*SI Appendix*, Fig. S9 *E*–*L*). These findings indicate that the thymus contributes not only to T-cell development but also to the generation of B cell–enriched lymphoid structures in CTs. The molecular mechanisms regulating CXCL12 expression in CD4^+^ and CD8^+^ T cells, as well as the involvement of T cells in the differentiation of B-cell precursors within CTs, require further study.

*Streptococcus* infection markedly impairs hepatic metabolic and immune functions (24–26). A major finding of the current study is that, in the absence of intestinal IgA production (caused via inhibition of B-cell migration to CTs), *Streptococcus* species such as *S. alactolyticus* and *Staphylococcus* species such as *S. chromogenes* and *S. epidermidis* frequently invade the liver and induce severe metabolic dysfunction and immune abnormalities (Fig. 5*I*). *S. alactolyticus*, which was frequently observed in the liver of AMD3100-treated chickens at D21 and D50, possesses genes involved in extracellular glycogen degradation such as *pulA* and may “steal” hepatic glycogen for metabolic support (27, 28). Beneficial *Lactobacillus* species such as *L. crispatus* were also commonly found in the liver, regardless of gut IgA status. Some strains of *L. crispatus* possess *amyX*, which encodes an endoamylase that can break down hepatocyte-derived glycogen (*SI Appendix*, Fig. S15*H*). However, the *pulA* gene highly conserved in *S. alactolyticus* encodes an enzyme that cleaves α-1,6-glycosidic bonds at glycogen branch points, releasing glucose, maltose, and other oligosaccharides (29). Conversely, *AmyX* primarily hydrolyzes α-1,4-glycosidic bonds to generate oligosaccharides. This difference in enzymatic activity may enable *S. alactolyticus* to better exploit hepatic glycogen, particularly branched glycogen structures that are inaccessible to many other bacteria, thereby facilitating its colonization and persistence. *S. alactolyticus* also possesses virulence factors (such as *cpsB* and *hasC*) that facilitate its colonization in the liver (*SI Appendix*, Fig. S15*G*). *AmyX* and *PulA* cooperate to completely degrade hepatic glycogen into oligosaccharides, which can subsequently be metabolized by other bacteria (30, 31); therefore, it is highly likely that coinfection by *S. alactolyticus* and *L. crispatus* enhances bacterial diversity in the liver. Consistent with this notion, hepatic bacterial diversity increased dramatically in IgA-deficient chickens at D50 due to the influx of pathogenic bacteria, accompanied by severe hepatic metabolic dysfunction and lymphocyte aggregation.

Kupffer cells located around the portal vein efficiently recognize IgA-coated bacteria via IgA Fc receptors (Fcα/μR and FcαRI) in mice and humans (32–34). Although the presence of such receptors by Kupffer cells has not yet been demonstrated in chickens, our findings raise the possibility that IgA coating of gut bacteria may prevent liver translocation by facilitating the phagocytic activity of Kupffer cells in chickens. Conversely, pathogens like *Streptococcus* and *Staphylococcus*, which are not recognized by intestinal IgA, may evade detection by Kupffer cells, leading to persistent hepatic inflammation once they cross the intestinal barrier (Fig. 5*J*). The specificity of intestinal IgA in preventing bacterial translocation from the gut to the liver warrants further investigation. Multiple commensal bacteria, such as *Lachnospiraceae*, are recognized by intestinal IgA with high sensitivity and may contribute to the formation of bacterial communities predominantly composed of IgA-coated resident microbes (*SI Appendix*, Fig. S13 *C* and *D*). This coating may be exploited therapeutically to prevent liver infection by gut-derived pathogens. Although a vaccine against pathogenic *Streptococcus* or *Staphylococcus* to prevent liver translocation may be ineffective owing to weak inherent immunogenicity, enhancing intestinal IgA-mediated bacterial communities could strengthen the mucosal barrier and prevent the translocation of pathogenic bacteria that are typically coated with intestinal IgA. Among commensal bacteria, *Lachnospiraceae* represent promising candidates for enhancing intestinal immunity, as they are strongly recognized by intestinal IgA produced by these newly identified BF-independent B cells and generate SCFAs, such as butyrate, that promote intestinal IgA synthesis (35–37). The specificity of IgA for *Lachnospiraceae* has also been confirmed in mice and humans, further supporting the protective potential of these microbes against intestinal inflammation and susceptibility to gastric and hepatic diseases (38, 39). We suggest that probiotic strategies employing beneficial bacteria with antigenic properties, such as *Lachnospiraceae*, represents a potential therapeutic approach for stimulating intestinal immunity through SCFA secretion and increased IgA coating of gut bacteria, thereby strengthening intestinal IgA–bacteria barrier.

In conclusion, this study demonstrates the differentiation of BM-derived B cells in intestinal CTs without BF and shows that these CT-resident B cells are essential for intestinal IgA production during posthatching maturation. The intestinal immune response conferred by CTs contributes to the establishment of a gut microbiome composed primarily of IgA-coated commensal bacteria, thereby limiting bacterial translocation and protecting against hepatic infection.

## Materials and Methods

Chickens, mice, cell lines, antibodies, and other materials used in this study are described in *SI Appendix*. Surgical treatment, oral immunization, adoptive cell transfer, and fecal material transfer were performed in chickens, and an anti-CXCL12 monoclonal antibody was generated by fusing splenocytes from immunized mice with myeloma cells. The number and distribution of immune cells were analyzed by flow cytometry and immunohistochemistry. IgA-coated and noncoated bacteria, as well as immune cells, were purified by magnetic-activated cell sorting. scRNA-seq and 16S rRNA amplicon sequencing data were analyzed using Seurat framework and QIIME 2 pipeline, respectively. Detailed and full experimental procedures are provided in *SI Appendix*.

## Supplementary Material

Appendix 01 (PDF)

Dataset S01 (XLSX)

Dataset S02 (XLSX)

Dataset S03 (XLSX)

Dataset S04 (XLSX)

Dataset S05 (XLSX)

Dataset S06 (XLSX)

Dataset S07 (XLSX)

Dataset S08 (XLSX)

Dataset S09 (XLSX)

Dataset S10 (XLSX)

Dataset S11 (XLSX)

Dataset S12 (XLSX)

Dataset S13 (XLSX)

Dataset S14 (XLSX)

Dataset S15 (XLSX)

Dataset S16 (XLSX)

Dataset S17 (XLSX)

## Data Availability

16S amplicon sequencing data, bulk RNA sequencing data, scRNA-seq data have been deposited in NCBI Sequence Read Archive (PRJNA1346295 (40), PRJNA1346473 (41), PRJNA1346141 (42), and PRJNA1400916 (43)). All other data are included in the manuscript and/or supporting information.
