# Supplementary material for: Bursa of Fabricius–independent B cells establish an IgA-mediated intestinal barrier that safeguards gut–liver homeostasis
Source: Proc Natl Acad Sci U S A. 2026 Jul 15;123(29):e2605569123. doi: 10.1073/pnas.2605569123 (PMC13389660; doi:10.1073/pnas.2605569123)
Supplement: Supplementary file 1 — Appendix 01 (PDF) [file pnas.2605569123.sapp.pdf]

PNAS

Supporting Information for

Bursa of Fabricius–independent B cells establish an IgA-mediated intestinal barrier that safeguards gut-liver homeostasis

Ryota Hirakawa[a,b,c], Motoshi Hisamatsu[a,b], Sayoko Maekawa[a,b], Eiki Asai[a,b], Miyuko Ohta[a,b], Ayumi Matsuo[d], Kunihiro Okano[d,e], Toh Miyazaki[f], Motofusa Akiyama[c,f], Masaaki Toyomizu[a,g], Jahidul Islam[a,b,c], Mutsumi Furukawa[a,b,c], Tomonori Nochi[a,b,c,h,i,j]

*Tomonori Nochi, Ph.D.

Email: nochi@tohoku.ac.jp

This PDF file includes:

Supporting text

Supplemental Figures S1 to S19

Legends for Datasets S1 to S17

SI References

Other supporting materials for this manuscript include the following:

Datasets S1 to S17

Supporting text

Methods and Materials

Animals

All animal experiments were conducted following the principles of the Basel Declaration, and animal protocols were approved by the Tohoku University Institutional Animal Care and Use Committee. Fertilized chicken eggs (Ross strain 308, *Gallus domesticus*) and male newborn chicks (day [D]0) were purchased from a commercial hatchery (Miyagi, Japan). Fertilized eggs were incubated at 38.0°C under 55% humidity until they reached the required Hamburger and Hamilton stage. All chickens were housed in wire cages maintained at 24°C–35°C and 55% or low humidity under a 24-h light/0 h dark photoperiod with a basal diet (21.0% crude protein and 2950 kcal/kg metabolizable energy) and water until the completion of the experiments. BALB/cAJcl and BALB/cAJcl-*nu*/*nu* mice used for generating monoclonal antibodies against avian CXCL12 (aCXCL12) and ascites containing these antibodies, respectively, were purchased from CLEA (Tokyo, Japan). In most experiments, D21 was considered an early stage during which BF-dependent B-cell development remains highly active, whereas D50 was a later stage characterized by more pronounced BF-independent immune maturation.

Bursectomy and thymectomy

The BF or thymus was surgically removed from 0-day-old chicks under anaesthesia (0.3 mg/kg medetomidine, 4 mg/kg midazolam and 5 mg/kg butorphanol tartrate), while whole CTs and cecal tissues were harvested at D21 and D50 as indicated to analyse the effects on immune cell populations by flow cytometry. Plasma samples and cecal contents were collected and stored at −30°C for subsequent enzyme-linked immunosorbent assays (ELISAs) or at −80°C for 16S rRNA amplicon sequencing analysis.

Oral immunization with *S. enterica*

Sham-operated healthy chickens, and chickens subjected to bursectomy were immunized with 1 × 10^9 colony-forming units (CFUs) of inactivated *S. enterica* (Kyoritsu Seiyaku, Tokyo, Japan) suspended in 0.5 mL sterilized PBS on D22 and D29 by oral administration. Plasma and fecal samples were collected from all chickens before immunization on D22 and D33 (4 days after booster immunization at D29), and stored at −30°C or −80°C for ELISAs.

Inhibition of the CXCR4$^+$ cell flux towards CXCL12

Normal healthy and bursectomized chickens were injected intraperitonially (i.p.) with 2 mg/kg AMD3100 (Abcam, ab120718) dissolved in sterilized PBS once every 3 days from D0 to D21 or to D50 as indicated. In some experiments, normal chickens were injected i.p. with 0.8 mg purified mouse anti-chicken CXCL12 monoclonal antibody (53D-10, mouse IgG_{1k}) generated in-house for this study or with a corresponding isotype control antibody (MOPC-21, mouse IgG_{1k}: Selleck Chemicals, A2106) at D7, D14 and D18 and then reared until D21. Tissues were harvested from all treatment groups at D21 or D50 as indicated to examine the effects on immune cell populations by flow cytometry. Cecal contents were also collected and stored at −80°C for ELISAs and 16S rRNA amplicon sequencing analysis.

BM transplantation

Normal healthy chickens (D22) were used as donors to isolate BM cells from the right femur, and the frequency of CXCR4$^+$ cells was investigated by flow cytometry. Untreated isolated BM cells or BM cells pretreated with 1 mg/mL AMD3100 (Abcam, ab120718) at 37°C for 30 min were adaptively transferred intravenously (5 × 10^8 per injection) into bursectomized chickens at D22. CTs and cecal tissues were harvested 7 days after transplantation (D29) to evaluate the recovery of immune cell populations by flow cytometry. Cecal contents were also collected at the indicated times and stored at −80°C for ELISAs.

Thymic T-cell transplantation

Thymus tissue samples were collected from normal healthy chickens (12 days of age) and thymic $CD4^+$ or $CD8\alpha^+$ cells purified by AutoMACS system (Miltenyi Biotec, Germany) after removing $CD4^+CD8\alpha^+$ and $CD4^-CD8\alpha^-$ cells. The purified $CD4^+$ and $CD8\alpha^+$ cells were injected intravenously (i.v.) at 3×10^6 cells per injection into 12-day-old recipient chickens thymectomized immediately after hatching. CTs and cecal tissues were harvested from recipients 10 days after thymocyte transplantation (D22) to evaluate the recovery of immune cell populations by flow cytometry. Cecal contents were also collected at the indicated times and stored at −80°C for ELISAs.

Transplantation of fecal preparations

Bursectomized and AMD3100-treated chickens were administered soluble proteins derived from the feces of healthy normal chickens to assess the effects of BF and CT loss on resistance to pathogenic gut microbes. Briefly, 2 g of fresh donor feces was suspended in sterilized PBS, filtered through medical gauze to completely remove large particles, and centrifuged at 15,000 rpm and 4°C for 10 min. The supernatant was collected and filtered through 0.20-μm pore filters (ADVANTEC, 25AS020AS) to completely remove live bacteria, then filtered through PD-10 Columns (Cytiva, 17085101) to exclude metabolites, and finally filtered through Amicon Ultra Centrifugal Filters with a 100-kDa molecular weight cutoff (Merck, UFC9100) to prepare fractions containing soluble proteins greater than or less than 100 kDa. Additionally, to prepare the IgA-depleted fraction, IgA was removed from the >100-kDa protein fraction prepared from feces collected one day before transplantation by affinity chromatography using a HiTrap™ NHS-activated HP column (Cytiva, 17071601) coupled with goat polyclonal anti-chicken IgA antibodies (Bethyl Laboratories). These three fecal protein preparations (>100kDA, IgA-depleted >100kDa, and <100 kDa) were orally administered once daily to the indicated treatment groups from D15 to D21. One day after the final administration (D22), plasma samples, cecal contents

and liver tissues were harvested from recipients and stored at −30°C or −80°C for subsequent histological, biochemical and 16S rRNA amplicon sequencing analyses.

Mouse cell line for hybridoma generation

Mouse myeloma cells (P3X63Ag8.653) were obtained from The European Collection of Authenticated Cell Cultures (Porton Down, UK) and cultured in RPMI 1640 medium (Gibco, 11875119) supplemented with 10% (v/v) fetal bovine serum (FBS; Biowest, Nuaillé, France) and 100 U/mL penicillin–100 U/mL streptomycin (Thermo Fisher Scientific, 15070-063).

Isolation of chicken immunocytes

Mononuclear cells were isolated from CTs, ileal Peyer's patches (PPs), cecum, colon, jejunum, BM, BF, thymus, spleen and liver. Briefly, intestinal tissues, including CTs, PPs, cecum, colon and jejunum, were first cut into 1–2-cm pieces and stirred in RPMI 1640 medium (FUJIFILM Wako Pure Chemical, 189-02025) containing 1 mM EDTA (Nippon gene, 311-90075) for 25 min at 37°C to remove the epithelial layer. Tissues were then minced and incubated in RPMI 1640 medium containing 10% FBS, 0.5 mg/mL collagenase (Sigma-Aldrich, C2139), 5 U/mL deoxyribonuclease I (Sigma-Aldrich, D4513) and 50 μM of β-mercaptoethanol (Nakalai Tesque, 21438-82) for 80 min at 37°C to isolate cells from the intestinal lamina propria. The liver was also enzymatically digested using the same method to obtain mononuclear cells. The cell suspension in enzyme solution was filtered through Falcon™ Cell Strainers (Corning, 352340) to isolate individual cells for flow cytometry. Alternatively, BM, BF, thymus and spleen samples were dispersed mechanically on Falcon™ Cell Strainers without enzyme treatment.

Expression and purification of recombinant avian (a)CXCL12

The full-length aCXCL12 gene, excluding the signal peptide-coding sequence, was amplified by PCR using cDNA synthesized from the pooled mRNA of healthy chicken tissues and

the primer pair (sense) 5’-ATCTGAGCTCTTCAAAGCTTTGGAGGAGAAGCCTGTCAGCCTGACT-3’ and (antisense) 5’-AGTACGCTCGAGTTACAATGCTTGCCTCTTACATCT-3’ (underlined sequences denote the *SapI* and *XhoI* restriction enzyme sites). The PCR amplicons and bacterial expression vector pPAL7 (Bio-Rad, Hercules, CA, USA) were digested with *SapI* (New England Biolabs, R0569S) and *XhoI* (Takara, 1094A) to generate compatible ends and subsequently ligated using the DNA Ligation Kit version 2 (Takara, 6022). Rosetta™ 2(DE3)pLysS competent cells (Novagen, 71403) were transformed with complete vector (paCXCL12-PAL7) and cultured for 6 h at 29°C in the presence of 0.5 mM isopropyl-β-D-1-thiogalactopyranoside (Takara, 9030) to induce the expression of recombinant aCXCL12. After washing, the bacterial cells were lysed by sonication in phosphate buffer (pH 7.2) and recombinant aCXCL12 was purified using a Profinity eXact Protein Purification System (Bio-Rad, Hercules, CA, USA). The purity of recombinant aCXCL12 was confirmed by SDS-PAGE using 5%–20% e-PAGEL polyacrylamide gels (ATTO, E-T520L).

Generation of CXCL12 monoclonal antibody

BALB/c mice were subcutaneously immunized with 10 µg of purified aCXCL12 together with TiterMax® Gold (TiterMax, G-1) as an adjuvant on three or four occasions at 2-week intervals. One week after the final immunization, splenic mononuclear cells were collected and subsequently fused with myeloma cells (P3X63Ag8.653) using PEG1500 (Roche, 10783641001), and hybridomas were negatively selected by removing unfused myeloma cells using hypoxanthine, aminopterin, thymidine (Gibco, 21060017) and HT (Gibco, 11067030). The hybridoma culture supernatants were collected to confirm the secretion of mouse IgG antibodies specific to aCXCL12 by ELISA. The isotype was determined using a rapid monoclonal antibody isotyping kit (Antagen Biosciences, ISO-M8A-5). One hybridoma clone (53D-10) secreting mouse IgG1κ specific to aCXCL12 was injected into the peritoneal cavity of BALB/cAJcl-*nu/nu* mice pretreated with 500 µL pristane (Funakoshi, 980-60542) to obtain ascites containing mouse anti-aCXCL12 monoclonal antibody. Antibodies were purified using Protein G Sepharose™ 4 Fast

Flow beads (Cytiva, 17061801), and a proportion labelled with Alexa Fluor 647 (Thermo Fisher Scientific, A20173) for immunostaining.

Chemotaxis assay

The chemotaxis of BM cells towards aCXCL12 was evaluated using the CytoSelect™ 96-well Cell Migration Assay (5 µm; Cell Biolabs, CBA-105). Briefly, BM cells were isolated from the left femurs of bursectomized chickens at approximately D50, filtered through Falcon™ Cell Strainers (Corning, 352340) and incubated with cell lysis buffer containing NH_4Cl (155 mmol/L), $KHCO_3$ (10 mmol/L) and EDTA (0.1 mmol/L) for 10 min at room temperature to remove erythrocytes. A fraction of the cells was treated with 1 mg/mL AMD3100 (Abcam, ab120718**)** in RPMI 1640 medium (FUJIFILM Wako Pure Chemical, 189-02025) containing 10% (v/v) FBS and 100 U/mL penicillin–100 U/mL streptomycin (Thermo Fisher Scientific, 15070-063) or vehicle for 30 min at room temperature. Treated and vehicle-control cells were washed twice and adjusted to a density of 1×10^5 per 100 µL in serum-free RPMI 1640 medium and added to the upper chamber of transwell cassettes, whereas chemotaxis medium (RPMI 1640 with 10% FBS) supplemented with 0, 10 or 100 ng/mL aCXCL12 was added to the bottom chamber. The transwell cassettes were incubated for 2.5 h at 37°C under a 5% CO_2 atmosphere and the migrating cells in the bottom chamber were subsequently lysed and quantified using CyQuantr^R GR fluorescent dye on a fluorescence plate reader. The assay was performed three times using BM cells obtained from three independently treated chickens.

Magnetic-activated cell sorting of T cells

Thymic cells of defined phenotypes were isolated for adoptive T-cell transfer using MACS. Briefly, thymic cells were isolated and incubated with mouse FITC-CD8α (CT-8, 50 µg/mL) and PE-CD4 (CT-4, 10 µg/mL) for 30 min at 4°C. To obtain $CD4^+CD8\alpha^-$ cells, total T cells were stained with anti-FITC MicroBeads (20 µL/10^7 cells; Miltenyi Biotec, 130-048-701) at 4°C for 30 min, and both $CD4^+CD8^+$ cells and $CD4^-CD8^+$ cells were removed via negative selection using

an AutoMACS system (Miltenyi Biotec). The remaining cells were then incubated with anti-PE MicroBeads (20 µL/1 × 10^7 cells; Miltenyi Biotec, 130-048-801) at 4°C for 30 min to collect the $CD4^+CD8\alpha^-$ cells through positive selection. To obtain $CD4^-CD8\alpha^+$ cells, total T cells were stained with anti-PE MicroBeads UltraPure at 4°C for 30 min, and $CD4^+CD8\alpha^+$ cells and $CD4^+CD8\alpha^-$ cells removed via negative selection by AutoMACS. The remaining cells were then incubated with anti-FITC Microbeads at 4°C for 30 min to collect $CD4^-CD8\alpha^+$ cells through positive selection. Similarly, $CD4^+$ T cells, $CD8\alpha^+$ T cells, and $Bu1^+$ B cells were isolated from CTs and BF by positive selection for qRT-PCR analysis.

Flow cytometry

Cell populations obtained as described were counted via staining with Turk's solution and flow cytometry. Subsequently, 1 million cells in solution or 50 µL samples of whole blood were incubated for 30 min at 4°C with fluorescent dye-conjugated antibodies against the following cell surface markers obtained from either Southern Biotech (Birmingham, AB, USA) or Bio-Rad: FITC-IgM (M-1, 10 µg/mL), FITC-CD3 (CT-3, 10 µg/mL), FITC-CD8α (CT-8, 10 µg/mL), FITC-MHC-II (2G11, 2.5 µg/mL), PE-Bu1 (AV20, 2 µg/mL), PE-CD4 (CT-4, 2 µg/mL), PE-CD45 (LT40, 2 µg/mL), PE-KUL01 (KUL01, 2 µg/mL), Cy5-CD8α (CT-8, 2 µg/mL), APC-CD45 (LT40, 2 µg/mL), PE/Cy7-CD4 (CT-4, 2 µg/mL) and CXCR4 (9D9, 2 µg/mL) labelled with Alexa Fluor 647 (Thermo Fisher Scientific, A30009). For intracellular staining of IgY, IgA and CXCL12, cells were fixed with 4% paraformaldehyde (Nacalai Tesque, 30525-89-4), permeabilized with 0.1% saponin (Nacalai Tesque, 30502-42) and stained with FITC-IgY (G-1, 500 ng/mL), FITC-IgA (A-1, 500 ng/mL) or Alexa Fluor 647-aCXCL12 (53D-10, 4 µg/mL) for 30 min. Cell viability solution (10 µL/test; BD Biosciences, 555815) was used to exclude dead cells from the analyses. The stained (viable) cells were stratified into distinct staining populations by multicolour analysis using an Attune™ NxT Acoustic Focusing Cytometer (Thermo Fisher Scientific) and FlowJo 1D software (BD Biosciences). Blood cells were analysed similarly following red blood cell lysis.

Histology

The indicated tissues (CTs and liver) were fixed in 4% (w/v) paraformaldehyde for 24 h, embedded in paraffin, cut into 5-μm sections, deparaffinized, and stained with H&E or PAS and Sirius red (liver sections only). Tissue images were obtained using either BZ-X800, BZ-900, or BZ-1000 microscope (Keyence, Osaka, Japan) and staining quantified using Fiji (ImageJ) v1.54p (US National Institutes of Health). To quantify collagen deposition area and lymphocytic infiltration, four images were randomly acquired from each liver tissue section.

Immunohistochemistry

Tissue sections (5 μm) prepared as described for histology were stained with antibodies to detect T-cell subsets, B cells and plasma cells secreting IgM, IgA, IgY or CXCL12. Briefly, sections were treated with REAL Target Retrieval Solution (DAKO, S1699) for 40 min at 98°C, incubated with 0.5% (w/v) blocking reagent (PerkinElmer, FP1020) for 30 min at room temperature, treated with primary antibodies overnight at 4°C, and subsequently incubated in secondary antibodies for 1 h at room temperature. Unlabelled primary antibodies were obtained from commercial sources (abcam, Southern Biotech, Bio-Rad or Bethyl) or generated in-house for this study. The latter group included anti-CD3 (SP7, rabbit IgG, 1:150), anti-chicken Bu1 (AV20, mouse IgG1$_{\kappa}$, 1:200), anti-chicken MHC-II (2G11, mouse IgG1$_{\kappa}$, 1:100), anti-chicken CXCR4 (9D9, mouse IgG2a$_{\kappa}$, 1:1,000), anti-chicken IgM (goat polyclonal, 1:2,000), and anti-chicken CXCL12 (53D-10, mouse IgG1$_{\kappa}$, 1.5 μg/mL). The following isotype control antibodies were used to confirm the specificity of the primary antibodies: rabbit IgG (EPR25A, 1:150, Abcam), mouse IgG1$_{\kappa}$ (MOPC-21, 1:100, Selleck Chemicals), mouse IgG2a$_{\kappa}$ (MCA1210, 1:1000, Bio-Rad) and goat IgG (polyclonal, 1:20,000–1:50,000, Bethyl Laboratories, Montgomery, TX, USA). Secondary antibodies were as follows: Histofine Simple Stain MAX PO (R) and (M) (undiluted, Nichirei Biosciences, Tokyo, Japan), Alexa Fluor 647-conjugated donkey anti-rabbit IgG (1:200, Jackson ImmunoResearch, West Grove, PA, USA), Alexa Fluor 647-conjugated donkey anti-goat IgG (1:200, Jackson ImmunoResearch), and Alexa Fluor 488-conjugated

donkey anti-mouse IgG (1:200, Jackson ImmunoResearch). Horseradish peroxidase (HRP) activity from conjugated antibodies was visualized using 3,3′ -diaminobenzidine tetrahydrochloride (Dojindo, D006). Immunostained sections were counterstained with either hematoxylin or 1 μg/mL DAPI (Dojindo, 340-07971). All tissue images were obtained using either BZ-X800, BZ-900, or BZ-1000 fluorescence microscope (Keyence) and staining was quantified using Fiji (ImageJ) v1.54p. Images were selected randomly to quantify cell density and frequency.

Fluorescence *in situ* hybridization (FISH)

Liver tissue samples were processed for FISH to detect invading microorganisms. Briefly, tissue sections prepared for histology were deparaffined, blocked with pre-hybridization buffer [20 mM Tris-HCl (pH 8.0), 0.9 M NaCl, 0.01% SDS and 40% formamide] containing 150 μg/mL sheared salmon sperm DNA (BioDynamics, F012) at room temperature for 1 h, and then incubated for 3.5 h in a dark humid chamber at 46°C with 2 pmol/μL of either EUB338 probe (5'-Cy3-GCTGCCTCCCGTAGGAGT-3') or non-EUB338 probe (5'-Cy3-CGACGGAGGGCATCCTCA-3') in the presence of 150 μg/mL sheared salmon sperm DNA. Sections were then rinsed with post-hybridization buffer [0.215 M NaCl, 0.02 M Tris (pH 7.5), 0.025 M EDTA] at 48°C for 15 min and counterstained with 1 μg/mL DAPI.

3D image analysis of CTs using Amira

Serial images were loaded into Amira software (Thermo Fisher Scientific) for superimposition and 3D visualization (1). Briefly, 54 serial sections stained with anti-chicken CXCL12 monoclonal antibody followed by HRP-conjugated anti-mouse IgG were used for 3D reconstruction of CTs. Follicular structures were defined by the accumulation of cells surrounded by collagen fibers and manually visualized using Amira to generate distinct images. In addition, $CXCL12^{+}$ cells were manually classified according to their location (outside or inside follicular structures). Images with vertical communities were automatically aligned and compiled into 3D images using Amira.

Laser capture microdissection (LMD)

Specific regions of interest within tissue sections mounted on MembraneSlides (Leica, 11505158) were microdissected using an LMD 6500/7000 system (Leica Microsystems, Wetzlar, Germany). The microbeam was operated at a power 35 with a cut speed of 36, and the dissected tissues were catapulted into 0.2-mL PCR tube caps. Three distinct regions in CTs were dissected: FR, IFR (not containing intestinal crypt and epithelial cells) and villi. Each region was captured in a single cap with an area exceeding 10,000,000 μm^2. After completing the sample collection, the tubes were capped, centrifuged at 15,000 ×*g* for 10 min, and kept on ice until RNA extraction.

RNA extraction and RT-PCR

Total RNA was extracted from harvested and microdissected tissues using the ReliaPrep™ RNA tissue miniprep system (Promega, Z6111), and from purified cell fractions collected by MACS by using the ReliaPrep™ RNA Cell Miniprep System (Promega, Z6011). Briefly, cDNAs were synthesized from total RNA using the PrimeScript RT reagent kit with oligo(dT) primers and random hexamers (Takara, RR037A) and qPCR was performed using SYBR premix Ex Taq II (Takara, RR820A) to determine the mRNA expression levels of CXCR4, CXCR5, CCL19, CXCL12, and CXCL13, with β-actin expression measured as the control for normalization. All primers were designed using the Perfect Real-time Support System (Takara) and expression data were obtained using a Dice real-time PCR system (Takara).

Bulk RNA-seq and data analysis

Total RNA was extracted from the liver tissues of sham-operated and bursectomized chickens administered AMD3100 at D21 (n = 4) and D50 (n = 4) using the ReliaPrep™ RNA tissue miniprep system (Promega, Z6111). Library preparation with polyA selection and next-generation sequencing (NGS) was performed by Azenta Life Sciences (Burlington, MA, USA).

The libraries were sequenced on the NovaSeq™ (Illumina, San Diego, CA, USA) platform in accordance with the manufacturer's instructions, generating 150/150-bp paired-end reads. The total number of raw sequence reads ranged from 20,748,728 to 33,589,340. Raw RNA-seq data (FASTQ files) were quality-assessed using FastQC v.0.12.1, and quality trimming was performed using fastp v0.22.0. The clean reads were mapped to the chicken genome (Gallus_gallus.bGalGal1.mat.broiler.GRCg7b) using HISAT2 v2.2.1 with default settings. The mapping rate of all samples exceeded 94%. Bam files were sorted and indexed using SAMtools v1.6. Assembly, gene annotation and quantification were performed using featureCounts v2.0.6, and the alignment rate of all samples exceeded 84.9%. TPMCalculator was used to quantity mRNA abundance directly from alignments by parsing BAM files. Principal component analysis (PCA) was performed on TMM-normalized read counts obtained from edgeR, and permutational multivariate analysis of variance (PERMANOVA) with 999 permutations was used to assess significant differences among the groups. Differentially expressed genes (DEGs) were identified using edgeR based on a cutoff $p < 0.05$ and subjected to Gene Ontology (GO) pathway enrichment analyses or gene set enrichment analysis using clusterProfiler package in R, with significance denoted by $p < 0.05$.

Single-cell (sc)RNA-seq and data analysis

Single-cell suspensions of BM and CTs isolated from healthy chickens at D50 were first analysed using an Attune™ NxT Acoustic Focusing Cytometer (Thermo Fisher Scientific) to confirm cell composition and viability. Libraries were constructed using the Chromium Next GEM Single Cell 5' GEM Kit v2 (10x Genomics, Pleasanton, CA, USA) and Dual Index Kit TT Set A (10x Genomics) with Chromium Controller Next GEM (10x Genomics) according to the manufacturer's instructions, and quality was confirmed using an Agilent 2100 bioanalyser (Agilent Technologies, Santa Clara, CA, USA). Subsequently, libraries with different indices were multiplexed and loaded onto a DNBSEQ-G400RS instrument according to the manufacturer's instructions. Sequencing was performed using a 2×100-bp paired-end configuration by Kazusa

DNA Research Institute (Chiba, Japan). The Cell Ranger v.9.0.0 pipeline was used to analyse the sequencing data generated by the Chromium Single Cell Gene Expression platform. The Ensembl gene annotation file (Gallus_gallus.bGalGal1.mat.broiler.GRCg7b.114.gtf) was filtered using Cell Ranger mkgtf to retain only protein-coding genes to build the reference. Raw sequence data were processed using the *Gallus gallus* reference genome. Data normalization, dimensional reduction, cell clustering and differential expression analyses were performed using Seurat v.5.3.1. For quality control, genes detected in fewer than three cells and cells expressing <200 genes were excluded from the count matrix before downstream analyses. Cell filtering thresholds were determined according to data distribution and expression patterns. Cells with >4,000 expressed genes were also excluded to avoid doublets. In addition, BM cells with a mitochondrial gene count >20% or a ribosomal RNA gene count >40%, and CT cells with a mitochondrial gene count >25%, a ribosomal RNA gene count >40% or a hemoglobin gene count >10% were also excluded to ensure cell quality. Finally, doublet cells were excluded using scDblFinder v.1.18.0. Each dataset was normalized independently using SCTransform. PCA was performed using RunPCA for linear dimension reduction. Uniform manifold approximation and projection (UMAP) was then performed on the top 30 principal components using RunUMAP. For cell clustering, a shared nearest neighbor graph was generated using FindNeighbors, and clustering was performed using FindClusters with a resolution of 0.5. Cluster-specific marker genes and DEGs were identified using the FindAllMarkers function based on a $p < 0.05$ cutoff (Dataset S1 and 2). Differentially expressed genes were subjected to GO pathway enrichment analyses using clusterProfiler package in R, with significance denoted by $p < 0.05$ (Dataset S3). To conduct RNA velocity analysis, loom files were generated using the velocyto.py pipeline v0.17.17 according to the developer’s instructions. The resulting loom files were subsequently imported into Scanpy v1.11.1 and processed with scVelo v0.3.3 for downstream velocity estimation and visualization. Briefly, the data were filtered and normalized, and one- and two-order moments were computed across 30 principal components and 30 neighbors. Transcriptional dynamics were modelled using the dynamical model, and RNA velocity pseudotime were estimated. The velocity length and confidence were also computed. CellRank 2 v2.0.7 was employed to determine both initial and

terminal states based on RNA velocity-derived transition probabilities (2). BCR sequences were reconstructed from 5′ scRNA-seq data using TRUST4 with a *Gallus gallus* immunoglobulin reference (3). The reconstructed sequences were subsequently reannotated using IgBLAST against custom chicken IGH and IGL germline reference databases generated from IMGT-registered sequences (4), and the resulting IgBLAST-derived IGHV/IGLV annotations were integrated into the Seurat metadata. Cells containing both IGHV and IGLV annotations were defined as paired IGH–IGL-positive cells and used for downstream analyses. The top-scoring IgBLAST V-gene call was assigned as the representative IGHV or IGLV gene for each cell, and gene-level usage frequencies were calculated for each B-cell state.

Bacterial 16S rRNA sequencing

Genomic DNA was extracted from IgA-coated and -uncoated bacteria obtained for IgA-seq or from the cecum using a Stool DNA Isolation Kit (Norgen Biotek, 27600) according to the manufacturer’s protocol for 16S rRNA sequencing analysis (5). In addition, liver (left lobe) tissues were isolated in sterilized PBS and mechanically dispersed through 40-µm Falcon™ Cell Strainers (Corning, 352340) within a biosafety cabinet. Bacterial genomic DNA without host DNA was then directly extracted from tissue suspensions using a QIAamp DNA Microbiome Kit (Qiagen, 51704) according to the manufacturer’s protocol (6). The V3 and V4 regions of the bacterial 16S rRNA gene were amplified by PCR using PrimeSTAR HS DNA polymerase (Takara, R010A) and the following primers (adapter tag sequences are underlined): forward primer mix (5′-TGCTCTTCCGATCTGACNNNCCTACGGGNGGCWGCAG-3′, 5′-TGCTCTTCCGATCTGACNNNNCCTACGGGNGGCWGCAG-3′, 5′-TGCTCTTCCGATCTGACNNNNNCCTACGGGNGGCWGCAG-3′, 5′-TGCTCTTCCGATCTGACNNNNNNCCTACGGGNGGCWGCAG-3′) and reverse primer mix (5′-CGCTCTTCCGATCTCTGNNNGACTACHVGGGTATCTAATCC-3′, 5′-CGCTCTTCCGATCTCTGNNNNGACTACHVGGGTATCTAATCC-3′, 5′-CGCTCTTCCGATCTCTGNNNNNGACTACHVGGGTATCTAATCC-3′, 5′-

CGCTCTTCCGATCTCTGNNNNNNGACTACHVGGGTATCTAATCC-3′). The PCR fragments obtained from the first-round PCR were amplified individually by the second-round PCR using forward (5′-CAAGCAGAAGACGGCATACGAGATxxxxxxxxxGTGACTGGAGTTCAGACGTGTGCTCTTCCGATCTGAC-3′) and reverse primers (5′-AATGATACGGCGACCACCGAGATCTACACxxxxxACACTCTTTCCCTACACGACGCTCTTCCGATCTCTG-3′), including nine and five base indices, respectively (denoted as ′xxxxxxxxx′ and ′xxxxx′, respectively), to distinguish each sample. All PCR products were then sequenced using the MiSeq platform (Illumina) with the MiSeq reagent kit v2 (500 cycles, Illumina).

Data analysis for 16S rRNA sequencing

Demultiplexed raw sequences were acquired from the Base Space Sequence Hub (Illumina), and raw FASTQ files were analysed using QIIME 2 (version 2023.2 for macOS and OS X) (7). The DADA2 pipeline was used for quality filtering, trimming, denoising, merging paired FASTQ files, obtaining the amplicon sequence variances (ASV) table and removing chimaeras. ASVs were classified using the Silva 138 reference database, which was trimmed to match the V3–V4 region of the 16S rRNA gene using extract-reads in QIIME 2 to improve classification specificity. For species-level taxonomic annotation, the representative sequence of each ASV was aligned against the NCBI nucleotide database using the BLAST algorithm. The species exhibiting the highest sequence similarity (up to three) were subsequently assigned to each ASV. Alpha diversity and beta diversity (weighted UniFrac dissimilarity) were calculated using the QIIME 2 script diversity plugin. Weighted UniFrac distance-based beta diversity was calculated at rarefaction depths, which corresponded to the minimum read counts among samples in each analysis, and visualized using principal coordinate analysis (PCoA). PERMANOVA with 999 permutations was performed on weighted UniFrac distances to assess significant differences among groups. Linear discriminant analysis was also conducted using LDS effect size (LEfSe) to identify the most discriminative taxa among the groups using MicrobiomeAnalyst 2.0 (8). Functional profiles based on Kyoto Encyclopedia of Genes and Genomes ortholog abundance

were inferred from 16S rRNA marker gene sequences using q2-picrust2. The contributions of individual bacterial species to predicted enzyme abundance were evaluated using standalone PICRUSt2 v2.6.2 (9). Distinct MetaCycle pathways among the groups were determined by edgeR with a threshold of $p < 0.05$, and commonly upregulated or downregulated pathways among all chickens with immune abnormalities compared with sham-operated chickens were visualized by a Venn diagram.

IgA-seq analysis

Microorganisms recognized by IgA in the cecum were identified by IgA-seq analysis as described previously with some modifications (5). In brief, fresh cecal contents were suspended in PBS and filtered through cell strainers with 40-μm pores to remove debris. Clarified suspensions containing 0.1 mg of cecal contents were blocked with 10% (v/v) normal rat serum (Sigma-Ardrich, R9759) and 0.5% (w/v) bovine serum albumin (BSA; Sigma-Ardrich, A3059) for 30 min at 4°C, and treated for 30 min at 4°C with 5 μg/mL PE anti-chicken IgA (A-1, Southern Biotech) and 500 nM SYTO 9, followed by anti-PE MicroBeads UltraPure (20 μL/test; Miltenyi Biotec, 130-105-639) for 30 min at 4°C. IgA-coated and -uncoated microorganisms were collected using an AutoMACS magnetic cell sorter (Miltenyi Biotec), and genomic DNA was extracted using a Stool DNA Isolation Kit. The proportions (%) of IgA-coated and uncoated SYTO 9^+ microorganisms in the cecal contents were analysed by flow cytometry using an Attune NxT Acoustic Focusing Cytometer. Procrustes analysis was performed using the vegan package in R to assess the similarities between IgA^+ and IgA^- microbial community structures. IgA-binding indices (probability ratio) were computed using the IgAScores by providing four inputs: the taxon abundances in the IgA^+ and IgA^- fractions, and the FACS-derived sizes of the IgA^+ and IgA^- bacterial populations. The probability ratio ranges from −1.0 (absent in the IgA^+ fraction) to 1.0 (absent in the IgA^- fraction). Using the probability ratio of 28 major bacterial families, PCA was performed to visualize the differences in IgA reactivity against intestinal microorganisms at 21 and 50 days of age. Missing values were imputed using multivariate imputation by chained equations

via the IterativeImputer function in scikit-learn. Pairwise Euclidean distances from Sham samples were calculated to assess the influence of bursectomy or AMD3100 treatment on IgA bacterial reactivities.

Estimation of bacterial loads in serum, intestine and liver by PCR

Bacterial abundances were estimated by PCR using cell-free DNA extracted from plasma using NucleoSpin® cfDNA XS (Takara, U0900A), from liver samples using the QIAamp DNA Microbiome Kit (Qiagen, 51704), and from cecal contents using the Stool DNA Isolation Kit (Norgen Biotek, 27600). Each 25-μL PCR mixture contained 2× TB Green® Premix Ex Taq™ II (Tli RNaseH Plus, Takara, RR820A), genomic DNA and 10 pmol of forward (5′-GTGSTGCAYGGYTGTCGTCA-3′) and reverse primer (5′-ACGTCRTCCMCACCTTCCTC-3′). A standard three-step PCR protocol was used as follows (10): denaturation at 94° C for 10 min; 40 cycles of 94°C for 60 s, annealing at 60°C for 60 s and elongation at 72°C for 90 s and final elongation at 72°C for 5 min. To prepare standard DNA, the 16S rRNA gene (146 bp, 5′-GTGGTGCACGGCTGTCGTCAGCTCGTGTCGTGAGATGTTGGGTTAAGTCCCGCAACGAGCGCAACCCTTATTGTTAGTTGCCATCATTCAGTTGGGCACTCTAGCGAGACTGCCGGTAATAAACCGGAGGAAGGTGTGGACGACGT-3′) was amplified from *S. alactolyticus* genomic DNA, and the amplicons were cloned into the pGEM-T Easy vector (Promega, A1360). The plasmids were then introduced into *Escherichia coli* JM109 competent cells (Takara, 9052) and extracted using a PureYield™ Plasmid Miniprep System (Promega, A1223) to generate a copy number-based standard curve.

Immunoglobulin quantification

Chicken cecal contents and feces were suspended in PBS (4 μL/mg), homogenized using a multi-bead shocker, and centrifuged at 15,000 rpm. The supernatant concentrations of each Ig subclass (IgY and IgA) in the cecal supernatant and serum were determined using ELISA as described (11). Briefly, 96-well ELISA plates (Thermo Fisher Scientific) were coated overnight at

4°C with either goat anti-chicken IgY or IgA polyclonal antibodies (500 ng/mL, Bethyl Laboratories) and blocked for 1 h at 20°C with 1% (w/v) BSA (Sigma-Aldrich, A3059) and 0.05% (v/v) Tween-20. Plates were then incubated with serially diluted fecal supernatant, serum samples or reference serum containing chicken IgY or IgA at known concentrations for 2 h at 20°C. To investigate antigen-specific antibody titer, other plates were coated overnight at 4°C with 2×10^8 CFU/well *S. enterica* used for the immunization study and then blocked for 1 h at 20°C with 0.05% (v/v) Tween-20. After blocking, plates were incubated with serially diluted plasma or fecal supernatant samples obtained before and 4 days after booster immunization for 2 h at 20°C. After washing, the plates were incubated with 100 ng/mL HRP-conjugated Ig subclass-specific goat polyclonal antibodies (Bethyl Laboratories) for 1 h at 20°C, and signals were developed using a tetramethylbenzidine microwell peroxidase substrate system (SeraCare Life Sciences, 5120-0050). The reaction was stopped by adding H_2SO_4, and the absorbance was measured at 450 nm. The concentration of each Ig subclass was then quantified based on a standard curve.

Western blotting

Fecal suspensions were mixed 1:1 with SDS sample buffer containing 62.5 μM Tris-HCl (pH 6.8), 2% (w/v) SDS, 10% (v/v) glycerol and 0.02% (w/v) bromophenol blue, lysed, and constituent proteins separated by SDS-PAGE using 5%–20% e-PAGEL polyacrylamide gels (ATTO, E-T520L). After electrophoresis, protein bands were stained directly with SimplyBlue™ SafeStain (Thermo Fisher Scientific, LC6060) or transferred onto Immobilon-P membranes (Millipore, IPVH20200) for immunoblotting. Briefly, membranes were blocked with 0.05% (v/v) Tween-20 and 2.5% (w/v) Difco™ skim milk (BD, 232100) overnight at 4°C and then treated with an HRP-conjugated goat anti-chicken IgA antibody diluted 1:10,000 (Bethyl Laboratories) for 1 h at 20°C. The reaction was developed using EzWestLumi plus reagent (ATTO, WSE-7120S) and signals detected using Ez-Capture MG (ATTO).

Assessment of bacterial translocation and viability in the liver

Chickens were euthanized, and liver tissues aseptically dissected. A 200-mg liver tissue sample for each animal was mashed through 45-μm cell strainers (Corning, 352340) with a plunger, washed with 2 mL sterile PBS, diluted (1-, 10- or 100-fold) and spread on modified Gifu Anaerobic Medium (SHIMADZU, 302054268) agar plates at 37°C for 24 h under anaerobic or aerobic conditions. The CFU were manually counted from three agar plates at each dilution. To assess bacterial viability, mashed tissue was centrifuged at 430 ×*g* to remove large debris including host cells, and the supernatant was centrifuged at 8,000 ×*g* for 10 min. The pellet was washed twice with sterile PBS and resuspended in 1 mL of sterile PBS, and live and dead bacteria were quantified using the Bioplorer Rapid Microbial Detection System (Koyo Sangyo, Tokyo, Japan).

Isolation of hepatic bacteria strains

Liver homogenate was spread and anaerobically cultured on modified Gifu Anaerobic Medium (SHIMADZU, 302054268) agar plates at 37°C for up to 48 h. Then, 288 colonies from D50 chickens (n = 12) and 123 colonies from D22 chickens (n = 13) were transferred to liquid medium (SHIMADZU, 302054336), anaerobically incubated for 24 h, and stored at −80°C with 25% (v/v) glycerol. The 16S rRNA gene (V1–V9) locus of each isolate was amplified by PCR using the F27 (5′-AGRGTTTGATYMTGGCTCAG-3′) and R1492 primers (5′-TACGGYTACCTTGTTACGACTT-3′) and sequenced. The paired FASTQ files were merged and trimmed using Ape and sequences aligned using the BLAST program. The species exhibiting the highest 16S rRNA sequence similarity were subsequently assigned to each hepatic isolate. Based on the BLAST results, 396 sequences exhibited ≥99% identity, 13 sequences exhibited ≥97% identity and 1 sequence each exhibited ≥95% and ≥92% identity to the reference sequences. The 16S rRNA gene sequences of 411 isolates are presented (Dataset S9, 16 and 17). A phylogenetic tree based on 16S rRNA gene sequences was constructed using the Neighbor-Joining method based on the Kimura two-parameter model in MEGA v12. Sites

containing gaps or missing data were partially deleted with a site coverage cutoff of 95%. Bootstrap analysis was performed with 1,000 replicates to assess the robustness of the tree topology.

Whole-genome sequencing and analysis of bacteria in liver

Genomic DNA of *S. alactolyticus* from the livers of chickens subjected to bursectomy and AMD3100 treatment was isolated using the DNeasy Blood & Tissue Kit (Qiagen, 60504) according to the manufacturer's instructions. A pre-lysis step was included for gram-positive bacteria using a solution consisting of 30 mg/mL lysozyme (FUJIFILM Wako Pure Chemical, 123-06721) and 50 U/mL mutanolysin (Sigma-Aldrich, M9901) dissolved in 1× TE buffer (Nippon Gene, 317-09281) (12). The bacterial genome was sequenced on a NovaSeq system according to the manufacturer's instructions, generating 150/150-bp paired-end reads. Whole-genome sequencing data from *Streptococcus alactolyticus* (29 isolates), *Staphylococcus chromogenes* (31 isolates), *Staphylococcus epidermidis* (37 isolates), *Lactobacillus crispatus* (41 isolates), *Lactobacillus oris* (six isolates), *Lactobacillus reuteri* (four isolates), *Lactobacillus salivarius* (five isolates), *Lactobacillus portuensis* (two isolates), *Lactobacillus johnsonii* (six isolates), *Enterococcus avium* (four isolates), *Enterococcus faecalis* (four isolates), *Escherichia fergusonii* (four isolates), *Shigella dysenteriae* (four isolates) and *Vagococcus fluvialis* (six isolates) were also obtained from the Sequence Read Archive using the SRA Toolkit (Dataset S11). Paired-end reads were quality-filtered and trimmed using fastp v0.22.0, and the resulting clean reads were used for *de novo* genome assembly with the SPAdes v4.0.0 careful option. The quality of the resulting assembly was assessed using QUAST v5.3.0 (Dataset S12) and CheckM v1.1.3 (Dataset S13). Assemblies (154 isolates) with >98% completeness and <10% contamination were considered high-quality, and retained for downstream functional annotation analyses. The taxonomic identity of our representative isolate was confirmed using FastANI v1.34 using the genomes of 46 *Streptococcus* species (Dataset S10). For the reference genome of *S. alactolyticus*, sequencing data of *S. alactolyticus* JCM31116 generated on the PacBio sequencing

platform were assembled using Flye v2.9.6. The assembled contigs were annotated using Prokka v1.13.0 with species-specific options. The presence of genes associated with metabolism and transport of glucose/polysaccharides were confirmed by Prokka. Virulence factors were detected using Abricate v1.0.1 with the corresponding virulence factor database (minimum DNA identity = 70%, minimum DNA coverage = 70%; Dataset S14).

Hepatic bacteria-specific ELISA

A hepatic bacteria-specific IgA ELISA was developed evaluating the infection profile. Briefly, single colonies of *L. crispatus* (five isolates), *L. gallinarum* (three isolates), *L. salivarius* (three isolates), *Limosilactobacillus balticus* (three isolates), *L. oris* (three isolates), *L. portuensis* (three isolates), L. *reuteri* (three isolates), *L. urinaemulieris* (one isolate), *S. chromogenes* (four isolates), *S. epidermidis* (four isolates), *S. alactolyticus* (eight isolates), *E. casseliflavus* (one isolate), *E. faecalis* (one isolate), *E. avium* (one isolate), *M. sciuri* (one isolate), *E. fergusonii* (one isolate), *Corynebacterium falsenii* (two isolates) and *V. fluvialis* (one isolate) from D50 chicken liver were anaerobically incubated in GAM medium (SHIMADZU, 302054220) for 10–24 h at 37°C. Bacterial cells were collected by centrifugation, suspended in sterile PBS, and heat-killed at 95°C for 25 min. Then, 96-well plates were coated overnight at 4°C with 100 µL of 20 µg/mL heat-killed bacterial suspension, blocked for 1 h at 20°C with 0.05% (v/v) Tween-20 containing 1% (w/v) BSA (Sigma-Ardrich, A3059), and incubated with 100 µL serially diluted IgA-containing cecal supernatants (adjusted to 20 µg/mL IgA) for 2 h at 20°C. Plates were then washed and incubated with 100 ng/mL HRP-conjugated goat anti-chicken IgA as the secondary antibody for 1 h at 20°C. Signals were developed using a tetramethylbenzidine microwell peroxidase substrate system (SeraCare Life Sciences, 5120-0050). The background was defined as the optical density from wells coated with bacterial isolates but incubated with IgA-free sterile PBS.

Serum chemistry assay

Blood samples were collected from the leftwing veins of chickens and centrifuged at 2,000 ×*g* to obtain serum. Serum concentrations of glucose, cholesterol, non-esterified fatty acids, triglycerides, phospholipid and alkaline phosphatase were measured using commercial test kits (FUJIFILM Wako Pure Chemical).

Liver lipid and glycogen analysis

Liver tissues (100 mg per animal) were homogenized in 1 mL PBS, mixed with chloroform:methanol (1:2, v/v), incubated for 1 h at 4°C, and centrifuged at 15,000 ×*g* for 5 min. Extracts in the lower layer including lipids were collected, evaporated under vacuum to dryness, and dissolved in isopropyl 2-ethyl-1-butanol:isopropanol:Triton X-100 (8:1:1, v/v). The triglyceride, cholesterol and phospholipid levels in the extracts were measured using commercial test kits (FUJIFILM Wako Pure Chemical). To measure glycogen contents, liver tissues were first homogenized in 25 mM citrate buffer (pH 4.2) containing 2.5 g/L NaF at 0°C and centrifuged at 14,000 ×*g* for 5 min to remove debris. The glycogen content was then measured using an EnzyChrom™ Glycogen Assay Kit (BioAssay Systems, E2GN-100).

Statistical analysis and bioinformatics

All statistical analyses were performed using GraphPad Prism version 8.4.3 (www.graphpad.com), R Studio (https://www.rstudio.com/) or Python 3.10.14 (https://www.python.org/). Based on failed tests for normality, paired datasets were compared using the nonparametric two-tailed Mann–Whitney U test and more than two datasets using the Kruskal–Wallis test with post hoc Dunnett’s multiple comparisons tests. Aggregate results are presented as the mean ± SEM, and $p < 0.05$ was considered statistically significant for all tests. Sample sizes and statistical tests are denoted in the figure legends. Two-dimensional sample classification was conducted using the k-means clustering algorithm scikit-learn implemented in Python. Partial least-squares path modelling was performed on datasets from chickens at D50

(Dataset S15) to quantify the effect size of bacterial IgA reactivity on the cecal microbiome, hepatic microbiome, and hepatic biological indicators using the PLS-PM package in R.

Supplemental Figures

Fig. S1. Oral immunization with heat-killed *Salmonella enterica* to bursectomized chickens.
(***A***) Measurements of serum and fecal IgY and IgA following oral immunization with heat-killed *S. enterica* (SE) or PBS to bursectomized [BF(−)] and Sham chickens (n = 8 per group). (***B***) Levels of *S. enterica*-specific fecal IgA and IgY following immunization in BF(−) and Sham chickens. (**C**) Levels of serum IgA and IgY following immunization in BF(−) and Sham chickens. Results obtained from samples collected from independent chickens are presented as mean ± SEM and were analyzed by the Kruskal–Wallis test followed by Dunn's multiple comparisons test. $^{*}p < 0.05$, $^{**}p < 0.01$, $^{***}p < 0.001$, NS, not significant; D, day.

Fig. S2. Tissue distribution of B cells in healthy chickens.
(***A***) Representative images of multiple immune tissues, including the thymus, bursa of Fabricius (BF), spleen, cecal tonsils (CTs) and ileal Peyer's patches and distinct regions of the intestine (cecum, colon and jejunum) from healthy D35 chickens immunostained for $Bu1^{+}$ B cells (green) and $CD3^{+}$ T cells (red). (***B***) Frequencies of immature pre-B cells ($Bu1^{high}IgM^{-}$) and $sIgM^{+}$ B cells ($Bu1^{low}IgM^{low}$ and $Bu1^{low}IgM^{high}$) within the $7AAD^{-}CD45^{+}$ population (n = 6 per tissue). (***C***) Representative immunofluorescence images of Bu1 (green) and IgM (red) in CTs from healthy chickens at D35. (***D***) Expression of Bu1 (green) and IgM (red) in each cell present in the follicular regions. Results obtained from samples collected from independent chickens are presented as mean ± SEM. White arrows denote FRs. Scale bars: 200 μm.

Fig. S3. Distribution of $CXCR4^{+}$ and $CXCL12^{+}$ cells in cecal tonsils (CTs).
(***A***) Scatterplots of cells expressing either Bu1 or CD79B. (***B***) Velocity length and confidence computed using dynamical modelling in scVelo. (***C***) Initial and terminal cell states inferred from RNA velocity data using CellRank 2 to estimate lineage transition probabilities. (***D***) Expression of CXCR4 within the $7AAD^{-}$ cell population of CTs from healthy chickens at D50 (n = 6 per group). (***E***) mRNA expression levels of *cxcr4* in $Bu1^{+}$ B cells purified from CTs or spleen of chickens at D35-D50 (n = 6 per group). (***F***) Representative immunohistochemical images of $CXCR4^{+}$ cells in CTs from healthy chickens at D50. FR, follicular region; IFR, interfollicular region. (***G***) Laser microdissection of FRs, IFRs and villi to measure the region-specific expression levels of *cxcl12*, *cxcl13* and *ccl19* (n = 4–10 per region). (***H***) SDS-PAGE to confirm the purity of recombinant aCXCL12 expressed in *Escherichia coli* using the pPAL7 expression vector. (***I***) Representative immunohistochemical images of $aCXCL12^{+}$ cells in CTs from healthy chickens at D50. Scale bars: 40 or 200 μm. (***J***) 3D images of FRs reconstructed from 54 serial tissue images using Amira detailing the distribution of $aCXCL12^{+}$ cells inside (blue) and outside (green) the FR. Scale bars: 50 μm. Results obtained from samples collected from independent chickens are presented as mean ± SEM and were analyzed by either the Kruskal–Wallis test followed by Dunn's multiple

comparisons test (***G***) or a two-sided Mann–Whitney U test (***D* and *E***). $^{*}p < 0.05$, $^{**}p < 0.01$; NS, not significant; ND, not detected; D, day.

Fig. S4. B-cell progenitors in chicken bone marrow (BM).

(***A***) Scatterplots of HBA1, HBBA and HBAD expression in erythroblast lineages, ITGB3 and GP9 in the platelet lineage, HDC and GATA2 in the mast cell lineage, Bu1, CD79B, PAX5, EBF1 and IKZF3 in the B-cell lineage, and CD3E and BCL11B in the T-cell lineage (projecting on the UMAP plot). (***B***) GO analysis of marker genes in cell cluster C10. (***C***) Velocity length and confidence computed using dynamical modelling in scVelo. (***D***) Fate probabilities inferred by CellRank 2 from RNA velocity data. (***E***) Frequency of CXCR4^{+} cells within the 7AAD^{-}CD45^{+} population of the right femur BM from healthy chickens (n = 5 per tissue). (***F***) Representative immunohistochemical images of CXCR4^{+} cells (brown) in the right femur BM from healthy chickens. (***G***) Chemotaxis of right femur BM cells from ~D50 bursectomy chickens towards recombinant aCXCL12. Results obtained from samples collected from independent chickens are presented as mean ± SEM and were analyzed using the Kruskal–Wallis test followed by Dunn's multiple comparisons test. $^{*}p < 0.05$. Scale bars: 200 μm.

Fig. S5. IGHV and IGLV gene usage in B-cell subsets of CTs.

(***A***) UMAP visualization of B-cell lineage cells colored by B-cell subset. (***B***) Scatterplots showing cells expressing AICDA. (***C***) UMAP visualization of cells with paired IGHV and IGLV annotations. (***D***) IGHV gene usage frequencies in immature and mature B cells. (***E***) IGLV gene usage frequencies in immature and mature B cells.

Fig. S6. Influence of mouse anti-chicken CXCL12 monoclonal antibody administration on B cell maintenance in CTs.

(***A***) Healthy chickens were injected three times with either mouse anti-chicken CXCL12 (53D-10, mouse IgG1$_{\kappa}$) or isotype control mouse IgG1$_{\kappa}$ at D7, D14 and D18. (***B***) Frequency of B cells (Bu1^{high}) within the BF 7AAD^{-}CD45^{+} population (n = 3–4 per treatment). (***C***) Frequencies of immature pre-B cells (Bu1^{high}IgM^{-}) and sIgM^{+} B cells (Bu1^{low}IgM^{low} and Bu1^{low}IgM^{high}) within the spleen 7AAD^{-}CD45^{+} population (n = 3–4 per treatment). (***D***) Frequencies of immature pre-B cells and sIgM^{+} B cells within the cecal tonsil 7AAD^{-}CD45^{+} population (n = 3–4 per treatment). Results obtained from samples collected from independent chickens are presented as mean ± SEM and were analyzed by a two-sided Mann–Whitney U test. $^{**}p < 0.01$. NS, not significant; D, day.

Fig. S7. Influence of bursectomy [BF(−)] or AMD3100 treatment on B cell maintenance in the spleen.

(***A***) Frequency of sIgM$^+$ B cells (Bu1^{low}IgM^{low} and Bu1^{low}IgM^{high}) in the spleen from sham-operated (Sham), bursectomized [BF(−)], and AMD3100-treated chickens (AMD) (n = 5–13 per group). (***B***) Representative immunofluorescence images of Bu1$^+$ B cells (green) and CD3$^+$ T cells (red) in the spleen for each group. (***C***) Bu1$^+$ area in the spleen (n = 5 per group). (***D***) Number of germinal centers (GCs) in the spleen (n = 5 per group). White arrows: GCs. Scale bars: 200 μm. Results obtained from samples collected from independent chickens are presented as mean ± SEM and were analyzed by the Kruskal–Wallis test followed by Dunn's multiple comparisons test. $^*p < 0.05$, $^{**}p < 0.01$, $^{***}p < 0.001$. NS, not significant; D, day.

Fig. S8. Influence of bursectomy on B cell maintenance in CTs at D50.
(***A***) Frequencies of immature pre-B cells (Bu1^{high}IgM$^-$) and sIgM$^+$ B cells (Bu1^{low}IgM^{low} and Bu1^{low}IgM^{high}) within the cecal tonsil 7AAD$^-$CD45$^+$ population for sham-operated (Sham) and bursectomized [BF(−)] chickens at D50 (n = 10–12 per group). (***B***) Representative immunofluorescence images of Bu1$^+$ B cells (green) and CD3$^+$ T cells (red) in CTs from Sham and BF(−) chickens at D50. White arrows: FR. Scale bars: 200 μm. (***C***) The Bu1$^+$ area in the total FR and IFR of CTs from Sham and BF(−) chickens (n = 6 per group). (***D***) Number and size of FRs in CTs from Sham and BF(−) chickens (n = 5 per group). Results obtained from samples collected from independent chickens are presented as mean ± SEM and were analyzed using a two-sided Mann–Whitney U test. $^*p < 0.05$. NS, not significant; D, day.

Fig. S9. Influence of thymectomy on B- and T-cell maintenance in the BF, spleen and CTs.
(***A***) Confirmation of thymectomy accuracy at D21 following surgery at D0. (***B***) Frequency of pre-B cells (Bu1^{high}) within the BF 7AAD$^-$CD45$^+$ population of Sham and thymectomy [TH(−)] chickens (n = 5–7 per group). (***C***) Frequencies of immature pre-B cells (Bu1^{high}IgM$^-$) and sIgM$^+$ B cells (Bu1^{low}IgM^{low} and Bu1^{low}IgM^{high}) within the spleen 7AAD$^-$CD45$^+$ population (n = 8–11 per group). (***D***) Numbers of CD4$^+$ cells (CD4$^+$CD8α$^-$) and CD8α$^+$ cells (CD4$^-$CD8α$^+$) within the CT 7AAD$^-$CD45$^+$CD3$^+$ cell population (n = 5 per group). (***E***) Four groups: Sham, TH(−), TH(−) chickens transplanted with thymic CD4$^+$ cells [TH(−)CD4$^+$] and TH(−) chickens transplanted with thymic CD8$^+$ cells [TH(−)CD8$^+$] at D12. (***F***) Frequencies of pre-B and sIgM$^+$ B cells in CTs (n = 5–19). (***G***) Immunofluorescence images of Bu1$^+$ B cells (green) and CD3$^+$ T cells (red) in CTs. White arrows: FR. Scale bars: 200 μm. (***H***) Quantification of the Bu1$^+$ B-cell area in the total FR and IFR (n = 5–11 per group). (***I***) Number and size of FRs in CTs (n = 5–11 per group). (***J***) mRNA expression levels of *cxcl12* in Bu1$^+$ cells purified from BF, Bu1$^+$ cells, CD4$^+$ cells, and CD8α$^+$ cells purified from CTs of chickens at D35-D50 (n = 6–12 per group). (***K***) Identification of CD4$^+$ and CD8$^+$ cells within the CXCL12$^+$ cell population of CTs (n = 24) and BF (n=13). (***L***) Representative immunofluorescence images of CXCL12$^+$CD3$^+$ T cells in CTs. White arrows: FR. Scale bars: 50 (high magnification) or 200 (low magnification) μm. Results obtained from samples collected from

independent chickens are presented as the mean ± SEM and were analyzed by either a two-tailed Mann–Whitney U test (***A-D***) or the Kruskal–Wallis test followed by Dunn's multiple comparisons test (***F*, *I*, and *J***). $^{*}p < 0.05$, $^{**}p < 0.01$, $^{***}p < 0.001$, $^{****}p < 0.0001$. D, day.

Fig. S10. Effect of adoptive transfer of BM-derived cells and thymic T cells on the intestinal IgA production.

(***A***) Frequencies of $Bu1^{-}IgA^{+}$ plasma cells in the cecum of bursectomized BF(−) chickens transplanted with PBS, BM cells (BMT) or BM cells pretreated with AMD3100 (AMD-BMT) at D22 (n = 5–15 per group). (***B***) Cecal IgA levels in BF(−) chickens transplanted with PBS, BMT or AMD-BMT (n = 5–12 per group). (***C***) Frequencies of $Bu1^{-}IgA^{+}$ plasma cells in the cecum of Sham, thymectomy [TH(−)] chickens, TH(−) chickens transplanted with thymic $CD4^{+}$ cells [TH(−)$CD4^{+}$] and TH(−) chickens transplanted with thymic $CD8^{+}$ cells [TH(−)$CD8^{+}$] at D12 (n = 5–11 per group). (***D***) Cecal IgA levels in Sham, TH(−) chickens, [TH(−)$CD4^{+}$] chickens, and [TH(−)$CD8^{+}$] chickens (n = 5–12 per group). Results obtained from samples collected from independent chickens are presented as mean ± SEM and were analyzed by the Kruskal–Wallis test followed by Dunn's multiple comparisons test. $^{*}p < 0.05$, $^{**}p < 0.01$. D, day.

Fig. S11. Effect of bursa of Fabricius (BF)-independent B cells on the metabolic characteristics of cecal microbiota.

(***A***) PCoA of the Jaccard distance based on Kyoto Encyclopedia of Genes and Genomes orthologs predicted from the 16S rRNA gene sequencing data of cecal samples from sham-operated (Sham), bursectomized [BF(−)], AMD3100-treated (AMD) and BF(−)/AMD chickens at D21 (n = 8–12 per group) and D50 (n = 6–8 per group, $p = 0.001$, PERMANOVA) using q2-picrust2. Two-dimensional sample classification was conducted using the k-means clustering algorithm. (***B***) Venn diagram comparing changes in MetaCyc pathways of the cecal microbiome among BF(−), AMD and BF(−)/AMD chickens at D21 and D50. (***C***) Top 10 significantly upregulated or downregulated metabolic pathways in the cecal microbiome of BF(−) and AMD chickens compared to Sham chickens at D21 and D50. Results obtained from samples collected from independent chickens were analyzed by the Kruskal–Wallis test followed by Dunn's multiple comparisons test. $^{****}p < 0.0001$. D, day.

Fig. S12. Structure of IgA-coated and uncoated gut microbiota in healthy and immune compromised chickens.

(***A***) Schematic diagram of IgA-seq to identify IgA^{+} and IgA^{-} microbiota in cecal contents from sham-operated (Sham), bursectomized [BF(−)] and AMD3100-treated chickens (AMD). **(B)** SYTO 9^{+} bacteria recognized by IgA (SYTO $9^{+}IgA^{+}$). (***C***) Alpha diversity of the IgA^{+} and IgA^{-} microbiota from Sham, BF(−) and AMD chickens (n = 6–8 per group and time point). (***D***) PCoA of weighted

UniFrac distance measurements based on the 16S rRNA gene sequencing of bacterial communities in IgA^+ and IgA^- fractions from Sham, BF(−), and AMD chickens (p = 0.001, PERMANOVA). (***E***) Procrustes analysis of the similarities between IgA-coated and -uncoated microbiota calculated using the weighted UniFrac distance based on the 16S rRNA gene sequence. Results obtained from samples collected from independent chickens are presented as mean ± SEM and were analyzed by the Kruskal–Wallis test with Dunn's multiple comparisons test. $^*p < 0.05$, $^{**}p < 0.01$, $^{***}p < 0.001$. NS, not significant; D, day; SS, sum of squares.

Fig. S13. IgA-coated bacteria in healthy and immune compromised chickens.
(***A* and *B***) PCA based on the IgA-binding probability ratio of 28 major bacterial families at D21 (***A***) and D50 (***B***). Black arrows: Bacterial contributions to the PC1 and PC2 scores. Two-dimensional sample classification was conducted using the k-means clustering algorithm. (***C***) The IgA-binding probability ratio calculated for bacterial ASVs that were detected in more than half of the samples within each group at D21 and D50. Black cells indicate no data. (***D***) IgA-binding probability ratio of f_*Lachnospiraceae* in the cecum of sham-operated (Sham), bursectomized [BF(−)] and AMD3100-treated chickens (AMD) at D21 and D50. Results obtained from samples collected from independent chickens are presented as mean ± SEM and were analyzed by the Kruskal–Wallis test with Dunn's multiple comparisons test. $^*p < 0.05$, $^{**}p < 0.01$, $^{***}p < 0.001$; D, day.

Fig. S14. Hepatic immune responses of chickens with intestinal immune abnormality.
(***A***) PCA of the normalized gene counts from bulk RNA sequencing analysis of liver tissues from sham-operated (Sham) and BF(−)/AMD3100 chickens at D21 and D50 (n = 4 per group and time point). (***B***) Volcano plot showing differentially expressed genes (DEGs) detected by bulk RNA-seq analysis in the liver of Sham and BF(−)/AMD chickens at D50 (n = 4 per group). (**C**) Gene set enrichment analysis of DEGs revealing five major immune- and lipid metabolism-related pathways at D50. (***D* and *E***) Transcripts per million of immune-related genes quantified using the TPMCalculator at D21 (***D***) and D50 (***E***). Results obtained from samples collected from independent chickens are presented as the mean ± SEM and were analyzed by a two-sided Mann–Whitney U test. $^*p < 0.05$. D, day.

Fig. S15. Difference in hepatic microbiome between healthy and immunocompromised chickens.
(***A***) Bacterial abundance as revealed by genomic DNA in plasma (n = 5 per group) and liver (n = 6 per group) of sham-operated (Sham) and BF(−)/AMD chickens. (***B***) Representative images of mGAM agar plates inoculated with liver homogenate from Sham and BF(−)/AMD chickens under an aerobic or anaerobic environment. (***C***) Fluorescence *in situ* hybridization using either EUB338 or cEUB338 to demonstrate differences in microbial abundance in the liver between Sham and

BF(−)/AMD chickens. (***D***) Numbers of live and dead bacteria in the livers of Sham and BF(−)/AMD chickens (n = 5–6 per group). (***E***) Phylogenetic trees based on the 16S rRNA gene sequences of bacterial species isolated from the livers of Sham, bursectomized [BF(−)], AMD3100-treated (AMD) and BF(−)/AMD chickens at D50. Numbers at the nodes indicate bootstrap support values based on 1,000 replicates. **(F)** Presence or absence of diverse microbes in the livers of chickens (n = 3 per group). (***G***) Raw whole-genome sequencing data from 184 isolates representing 14 bacterial species retrieved from the NCBI Sequence Read Archive and assembled de novo using SPAdes. A total of 154 high-quality assemblies (completeness >98% and contamination <10%) were retained for downstream analyses. The number of virulence-associated genes were analyzed in four representative bacterial species, including a commensal species (*Lactobacillus crispatus*) and three non-commensal species (*Streptococcus alactolyticus*, *Staphylococcus epidermidis* and *Staphylococcus chromogenes*). The presence (red) or absence (green) of virulence-associated genes in *S. alactolyticus* (27 isolates) is shown as a heatmap. (***H***) Heatmap showing the presence (blue) or absence (white) of genes associated with polysaccharide and monosaccharide metabolism in each isolate. *Streptococcus alactolyticus* isolated from our samples is indicated by a red arrow. (***I***) Prediction of microbial contributions to *pulA* abundance in the livers of chickens at D50 based on PICRUSt2 analysis. Results obtained from samples collected from independent chickens are presented as the mean ± SEM and were analyzed by a two-sided Mann–Whitney U test (***A*, *D***) or the Kruskal–Wallis test with Dunn's multiple comparisons test (***G***). $^{*}p < 0.05$, $^{**}p < 0.01$, $^{***}p < 0.001$, $^{****}p < 0.0001$. D, day.

Fig. S16. Diverse effects of intestinal IgA on hepatic homeostasis in chickens.
(***A***) PCA based on the cecal IgA reactivities against 48 bacteria isolates from the liver. Cecal IgA were collected from sham-operated (Sham), bursectomized [BF(−)] and AMD3100-treated chickens (AMD) at D50. (***B***) The reactivity of cecal IgA against *Streptococcus alactolyticus* (8 isolates) and *Lactobacillus crispatus* (5 isolates) at D50.
(***C***) Species-level classifications in *Cutibacterium* and *Lactobacillus*, which were both increased in the liver by AMD3100 treatment, were performed with BLAST algorithm.
(***D***) Partial least-squares path modelling showing the effects of intestinal IgA–bacteria specificity on the gut microbiome, hepatic microbiome, hepatic glycogen content and hepatic lipid level. Significant paths ($p < 0.05$) are shown as red (positive) or blue (negative) arrows. D, day.

Fig. S17. Restoration of bacterial IgA coating by administration of IgA-containing fecal preparations from healthy chickens.
(***A***) Phylogenetic diversity of the cecal microbiome from BF(−)/AMD chickens treated with an IgA-enriched fecal preparation (IgA-enrich) and BF(−)/AMD chickens treated with an IgA-non-enriched fecal preparation (IgA-non, n = 5 per group). (***B***) PCoA of weighted UniFrac distances (n

= 5 per group). (***C***) Relative abundance of *Streptococcus* in cecum (n = 5 per group). (***D***) PCoA of weighted UniFrac distance measurements based on the 16S rRNA gene sequencing of bacterial communities in IgA^+ and IgA^- fractions (n = 5 per group). (***E***) IgA-binding probability ratio of bacteria (ASV) calculated using IgAScores (n = 5 per group). (***F and G***) IgA-binding probability ratio of g_*Streptococcus*, the members of commensal f_*Lachnospiraceae* and g_*Eubacterium coprostanoligenes* (n = 5 per group). Results obtained from samples collected from independent chickens are presented as the mean ± SEM and were analyzed by either a two-tailed Mann–Whitney U test (***F* and *G***) or the Kruskal–Wallis test followed by Dunn's multiple comparisons test (***A-C***). $^*p < 0.05$, $^{**}p < 0.01$, $^{****}p < 0.0001$. NS, not significant.

Fig. S18. Restoration of hepatic disorders in IgA-deficient chickens by administration of IgA-containing fecal preparations from healthy chickens.

(***A***) The frequencies of MHC-II^+ cells and $KUL01^+$ macrophages within the liver $7AAD^-CD45^+$ population (n = 5 per group). (***B***) Frequencies of $CD4^+$ cells and $CD8\alpha^+$ cells within the liver $7AAD^-$ population of all recipients (n = 5 per group). (***C***) Serum biochemical parameters analyzed using commercial kits (n = 5 per group). (***D***) Presence or absence of various microbes in the liver (n = 3–5 per group), with red representing positive detection and green representing negative detection. Results obtained from samples collected from independent chickens are presented as the mean ± SEM and were analyzed by the Kruskal–Wallis test with Dunn's multiple comparisons test. $^*p < 0.05$, $^{**}p < 0.01$. NS, not significant.

Fig. S19. Effect of administration of IgA-depleted >100-kDa fecal fraction on the cecal IgA and IgA-coated microbes in IgA-deficient chickens.

(***A***) Total amount of protein and IgA transplanted by the IgA-depleted >100-kDa fecal fraction. (***B***) Total IgA concentrations in cecal contents after transplantation (n = 5 per group). (***C***) Frequencies of IgA-coated microbes after transplantation (n = 5 per group). Results obtained from samples collected from independent chickens are presented as the mean ± SEM and were analyzed by the Kruskal–Wallis test with Dunn's multiple comparisons test. $^{**}p < 0.01$.

Legend of Datasets

Dataset S1. Top 10 Differentially expressed genes in all cell cluster in cecal tonsils (associated with Fig. 2*E*).

Dataset S2. Top 10 differentially expressed genes in all cell cluster in bone marrow (associated with Fig. 3*A*).

Dataset S3. Enrichment pathway of cell cluster-10 in bone marrow (associated with Fig. S4*B*).

Dataset S4. Enriched GO terms in the liver of Sham chickens compared to BF(-)/AMD treated chickens at D21.

Dataset S5. Enriched GO terms in the liver of BF(-)/AMD treated chickens compared to Sham chickens at D21.

Dataset S6. Enriched GO terms in the liver of Sham control chickens compared to BF(-)/AMD treated chickens at D50.

Dataset S7. Enriched GO terms in the liver of BF(-)/AMD treated chickens compared to Sham chickens at D50.

Dataset S8. Results of gene set enrichment analysis (GSEA) at D50 (associated with Fig. S14*C*).

Dataset S9. 16S rRNA gene sequences and BLAST alignment results of 288 bacterial isolates from the liver (associated with Fig. 5*I*).

Dataset S10. Average nucleotide identity (ANI) between the representative hepatic *Streptococcus alactolyticus* isolate from this study and genomes from 46 *Streptococcus* species.

Dataset S11. Source of whole-genome sequencing data used in Fig. S15*G* and *H.*

Dataset S12. Quality of genome assemblies of bacterial isolates were confirmed by QUAST.

Dataset S13. Analysis of the completeness and contamination of assembled genomes by CheckM.

Dataset S14. Identification of virulence factors of *Streptococcus alactolyticus* (27 isolates), *Lactobacillus crispatus* (30 isolates), *Staphylococcus chromogenes* (30 isolates), and *Staphylococcus epidermidis* (30 isolates) using ABRicate and the VFDB database (associated with Fig. S15*G*).

Dataset S15. Datasets used for partial least squares path modeling in R (associated with Fig. S16*D*)

Dataset S16. 16S rRNA gene sequences and BLAST alignment results of 63 bacterial isolates from the liver (associated with Fig. 6*K*)

Dataset S17. 16S rRNA gene sequences and BLAST alignment results of 60 bacterial isolates from the liver (associated with Fig. 6*O*)

SI References

1. A. Teshigahara *et al.*, Formation of the junctions between lymph follicles in the Peyer's patches even before postweaning activation. *Sci Rep* 14, 15783 (2024).
2. M. Lange *et al.*, CellRank for directed single-cell fate mapping. *Nat Methods* 19, 159-170 (2022).
3. L. Song *et al.*, TRUST4: immune repertoire reconstruction from bulk and single-cell RNA-seq data. *Nat Methods* 18, 627-630 (2021).
4. J. Ye, N. Ma, T. L. Madden, J. M. Ostell, IgBLAST: an immunoglobulin variable domain sequence analysis tool. *Nucleic Acids Res* 41, W34-40 (2013).
5. K. Usami *et al.*, The gut microbiota induces Peyer's-patch-dependent secretion of maternal IgA into milk. *Cell Rep* 36, 109655 (2021).
6. S. P. Pandey *et al.*, Tet2 deficiency drives liver microbiome dysbiosis triggering Tc1 cell autoimmune hepatitis. *Cell Host Microbe* 30, 1003-1019.e1010 (2022).
7. E. Bolyen *et al.*, Reproducible, interactive, scalable and extensible microbiome data science using QIIME 2. *Nat Biotechnol* 37, 852-857 (2019).
8. J. Chong, P. Liu, G. Zhou, J. Xia, Using MicrobiomeAnalyst for comprehensive statistical, functional, and meta-analysis of microbiome data. *Nat Protoc* 15, 799-821 (2020).
9. G. M. Douglas *et al.*, PICRUSt2 for prediction of metagenome functions. *Nat Biotechnol* 38, 685-688 (2020).
10. J. C. Leinwand *et al.*, Intrahepatic microbes govern liver immunity by programming NKT cells. *J Clin Invest* 132 (2022).
11. R. Hirakawa *et al.*, Heat Stress Causes Immune Abnormalities via Massive Damage to Effect Proliferation and Differentiation of Lymphocytes in Broiler Chickens. *Front Vet Sci* 7, 46 (2020).
12. A. Kalizang'oma *et al.*, Population genomics of *Streptococcus mitis* in UK and Ireland bloodstream infection and infective endocarditis cases. *Nat Commun* 15, 7812 (2024).

Figure S1

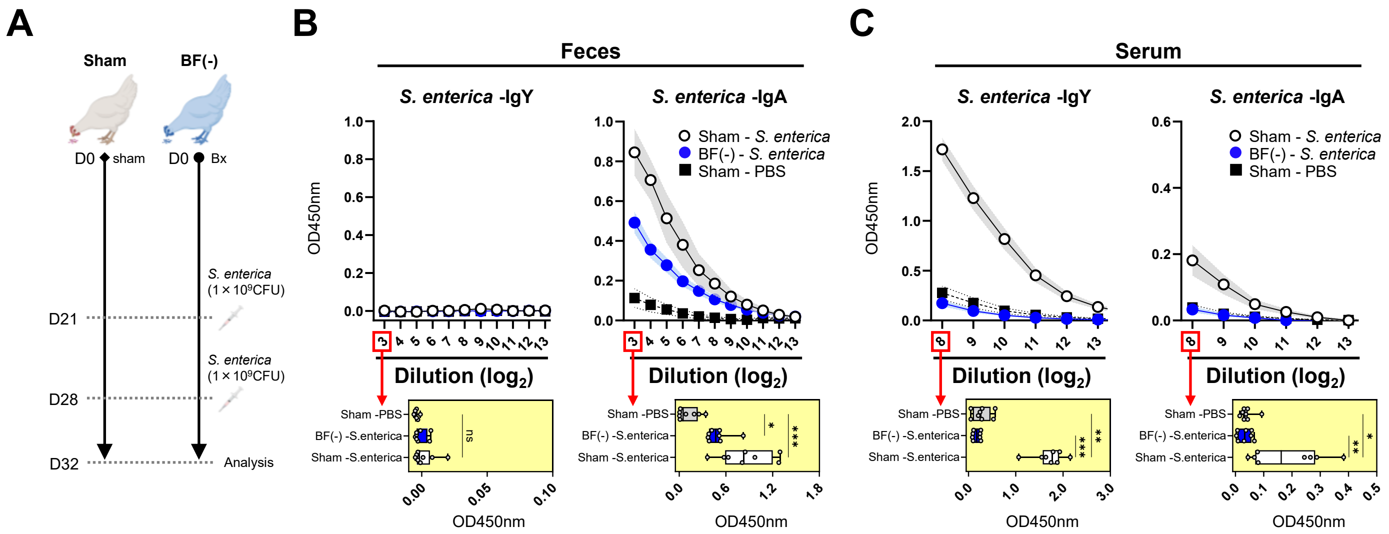

A
Sham
BF(-)
D0
sham
D0
Bx
S. enterica
(1 × 10⁹CFU)
D21
S. enterica
(1 × 10⁹CFU)
D28
D32
Analysis
B
Feces
S. enterica -IgY
S. enterica -IgA
OD450nm
Dilution (log2)
Sham - S. enterica
BF(-) - S. enterica
Sham - PBS
Sham -PBS
BF(-) -S.enterica
Sham -S.enterica
ns
OD450nm
C
Serum
S. enterica -IgY
S. enterica -IgA
OD450nm
Dilution (log2)
OD450nm

Figure S2

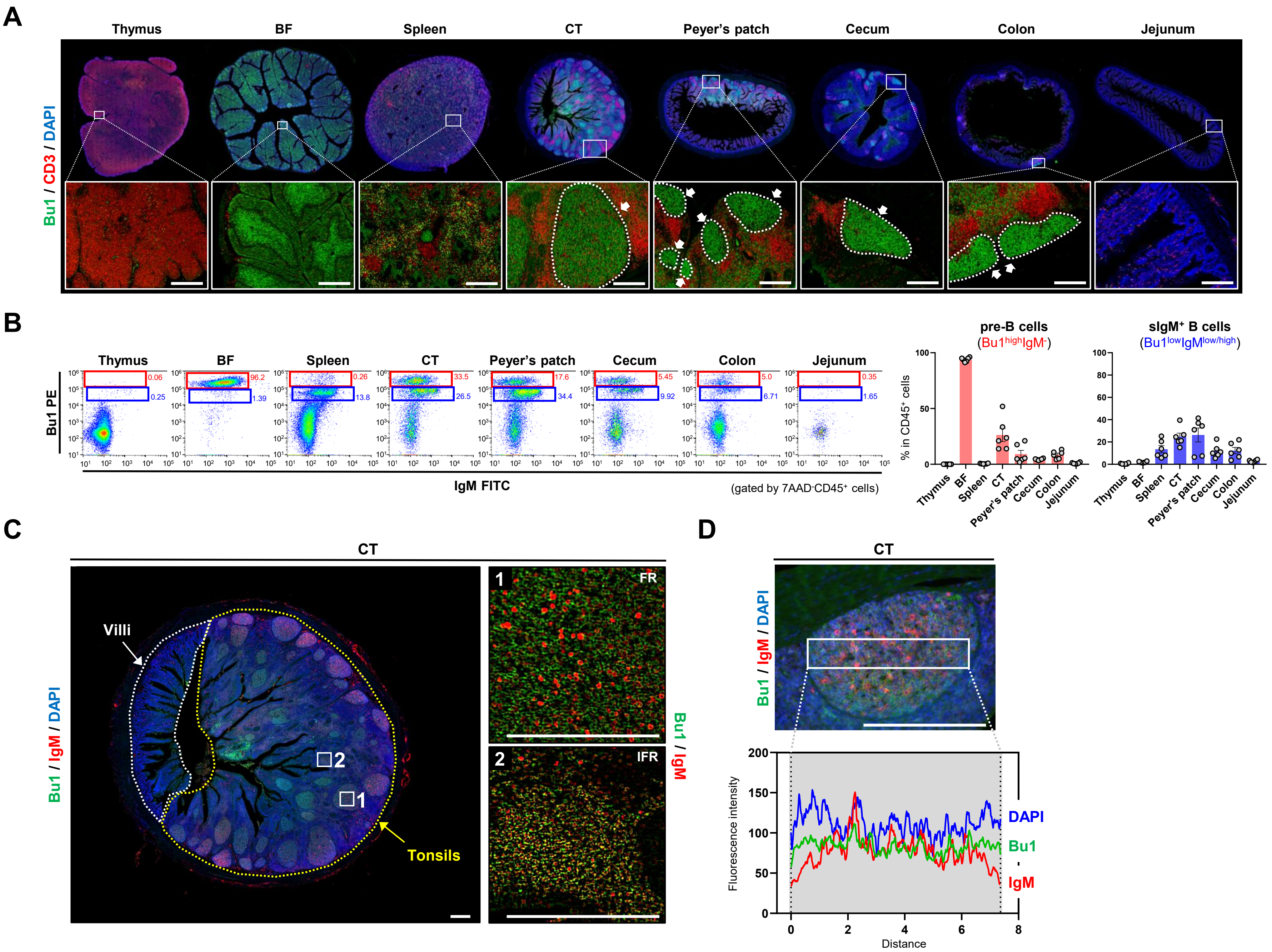
A
Thymus
BF
Spleen
CT
Peyer's patch
Cecum
Colon
Jejunum
Bu1 / CD3 / DAPI
B
Bu1 PE
IgM FITC
(gated by 7AAD-CD45+ cells)
0.06
0.25
96.2
1.39
0.26
13.8
33.5
26.5
17.6
34.4
5.45
9.92
5.0
6.71
0.35
1.65
pre-B cells
(Bu1highIgM-)
sIgM+ B cells
(Bu1lowIgMlow/high)
% in CD45+ cells
C
CT
Villi
Tonsils
Bu1 / IgM / DAPI
1
FR
2
IFR
Bu1 / IgM
D
CT
Bu1 / IgM / DAPI
Fluorescence intensity
Distance
DAPI
Bu1
IgM

Figure S3

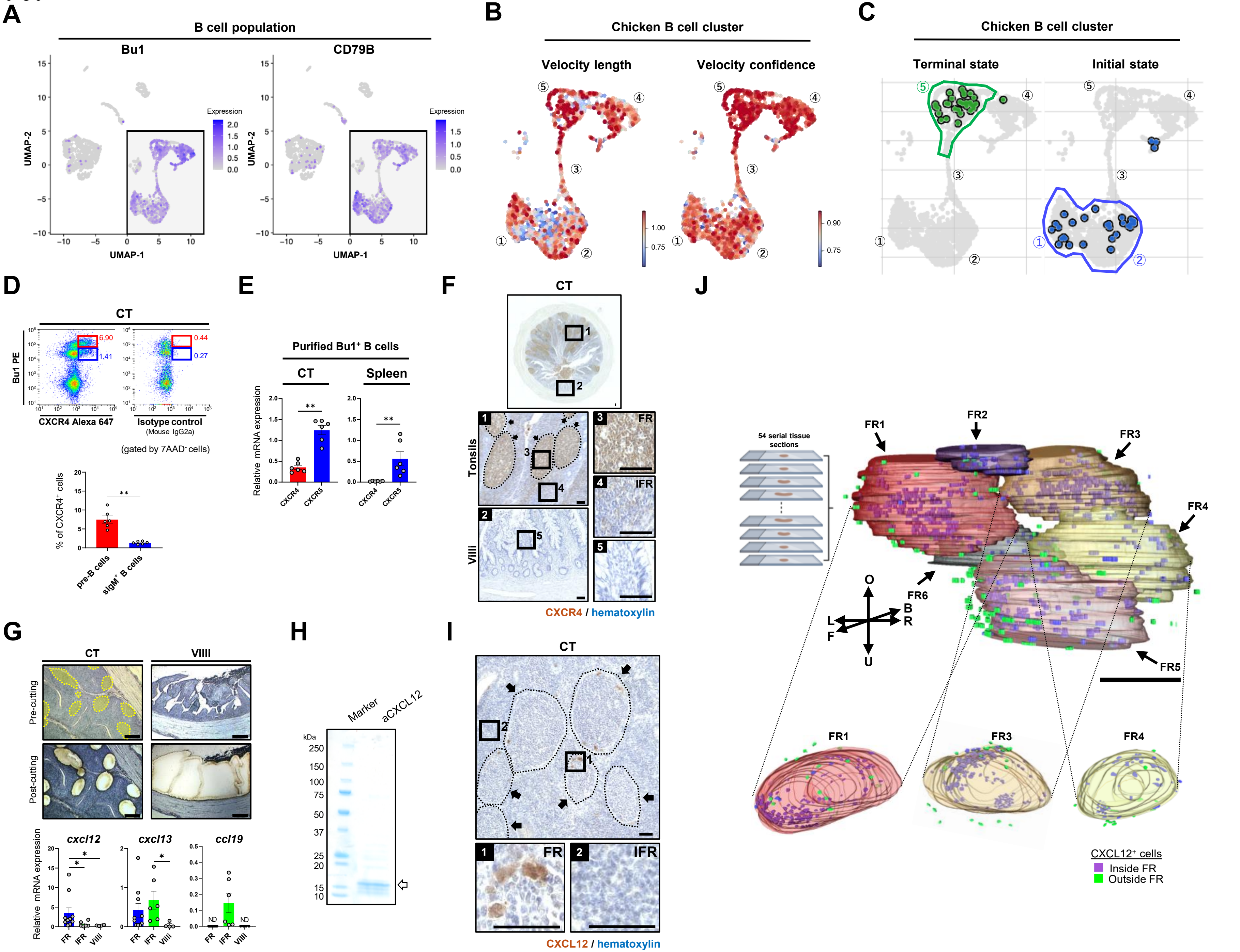

A
B cell population
Bu1
CD79B
UMAP-2
UMAP-1
Expression
B
Chicken B cell cluster
Velocity length
Velocity confidence
C
Chicken B cell cluster
Terminal state
Initial state
D
CT
Bu1 PE
CXCR4 Alexa 647
Isotype control
(Mouse IgG2a)
(gated by 7AAD- cells)
% of CXCR4+ cells
pre-B cells
sIgM+ B cells
E
Purified Bu1+ B cells
CT
Spleen
Relative mRNA expression
CXCR4
CXCR5
F
CT
Tonsils
Villi
FR
IFR
CXCR4 / hematoxylin
G
CT
Villi
Pre-cutting
Post-cutting
cxcl12
cxcl13
ccl19
Relative mRNA expression
FR
IFR
Villi
ND
H
Marker
aCXCL12
kDa
250
150
100
75
50
37
25
20
15
10
I
CT
FR
IFR
CXCL12 / hematoxylin
J
54 serial tissue sections
FR1
FR2
FR3
FR4
FR5
FR6
O
U
L
F
B
R
CXCL12+ cells
Inside FR
Outside FR

Figure S4

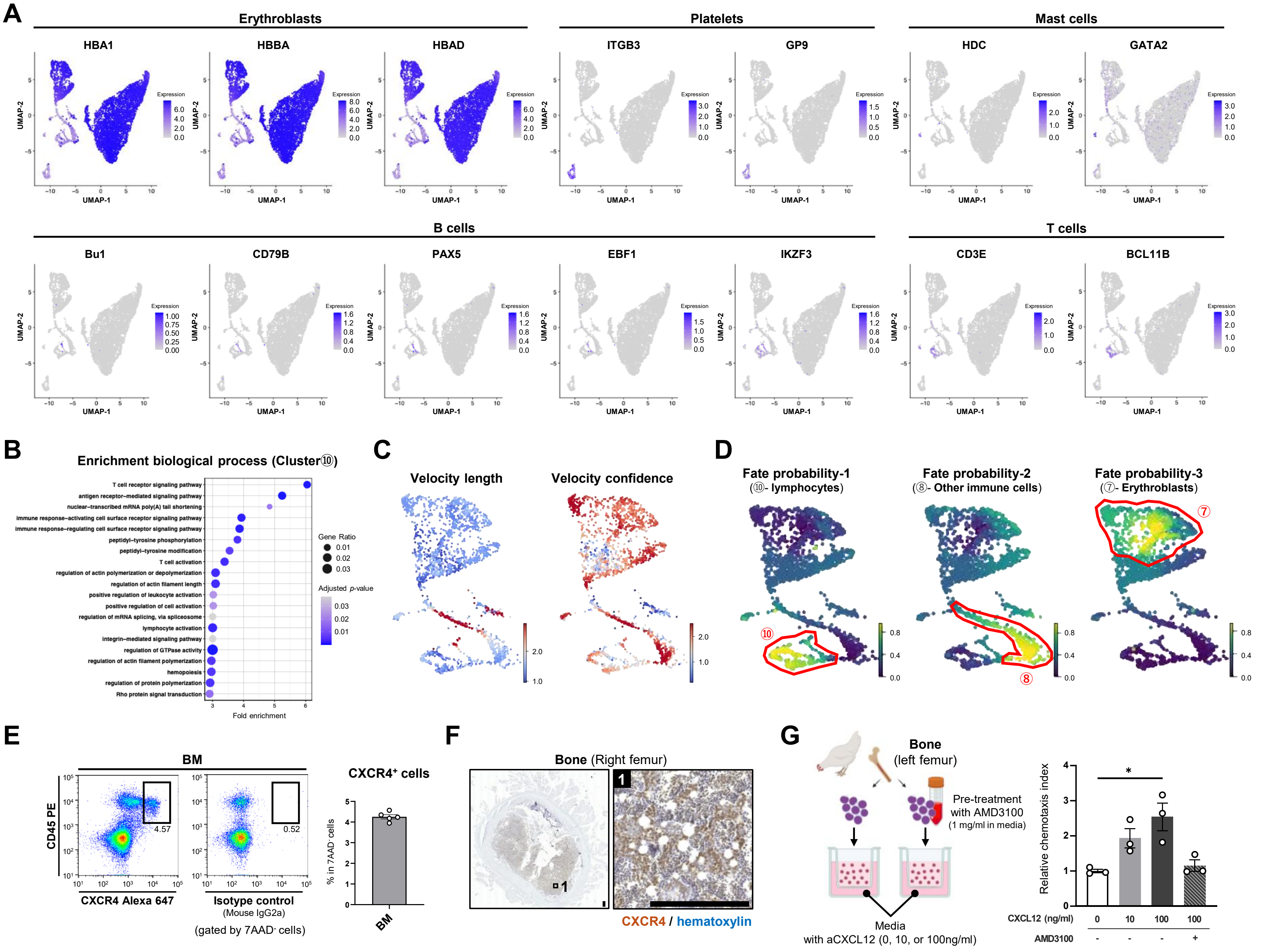

A
Erythroblasts
Platelets
Mast cells
HBA1
HBBA
HBAD
ITGB3
GP9
HDC
GATA2
B cells
T cells
Bu1
CD79B
PAX5
EBF1
IKZF3
CD3E
BCL11B
UMAP-1
UMAP-2
Expression
B
Enrichment biological process (Cluster⑩)
T cell receptor signaling pathway
antigen receptor-mediated signaling pathway
nuclear-transcribed mRNA poly(A) tail shortening
immune response-activating cell surface receptor signaling pathway
immune response-regulating cell surface receptor signaling pathway
peptidyl-tyrosine phosphorylation
peptidyl-tyrosine modification
T cell activation
regulation of actin polymerization or depolymerization
regulation of actin filament length
positive regulation of leukocyte activation
positive regulation of cell activation
regulation of mRNA splicing, via spliceosome
lymphocyte activation
integrin-mediated signaling pathway
regulation of GTPase activity
regulation of actin filament polymerization
hemopoiesis
regulation of protein polymerization
Rho protein signal transduction
Fold enrichment
Gene Ratio
0.01
0.02
0.03
Adjusted p-value
0.03
0.02
0.01
C
Velocity length
Velocity confidence
D
Fate probability-1
(⑩- lymphocytes)
Fate probability-2
(⑧- Other immune cells)
Fate probability-3
(⑦- Erythroblasts)
E
BM
CD45 PE
CXCR4 Alexa 647
4.57
Isotype control
(Mouse IgG2a)
0.52
(gated by 7AAD- cells)
CXCR4+ cells
% in 7AAD- cells
BM
F
Bone (Right femur)
CXCR4 / hematoxylin
G
Bone
(left femur)
Pre-treatment
with AMD3100
(1 mg/ml in media)
Media
with aCXCL12 (0, 10, or 100ng/ml)
Relative chemotaxis index
CXCL12 (ng/ml)
AMD3100

Figure S5

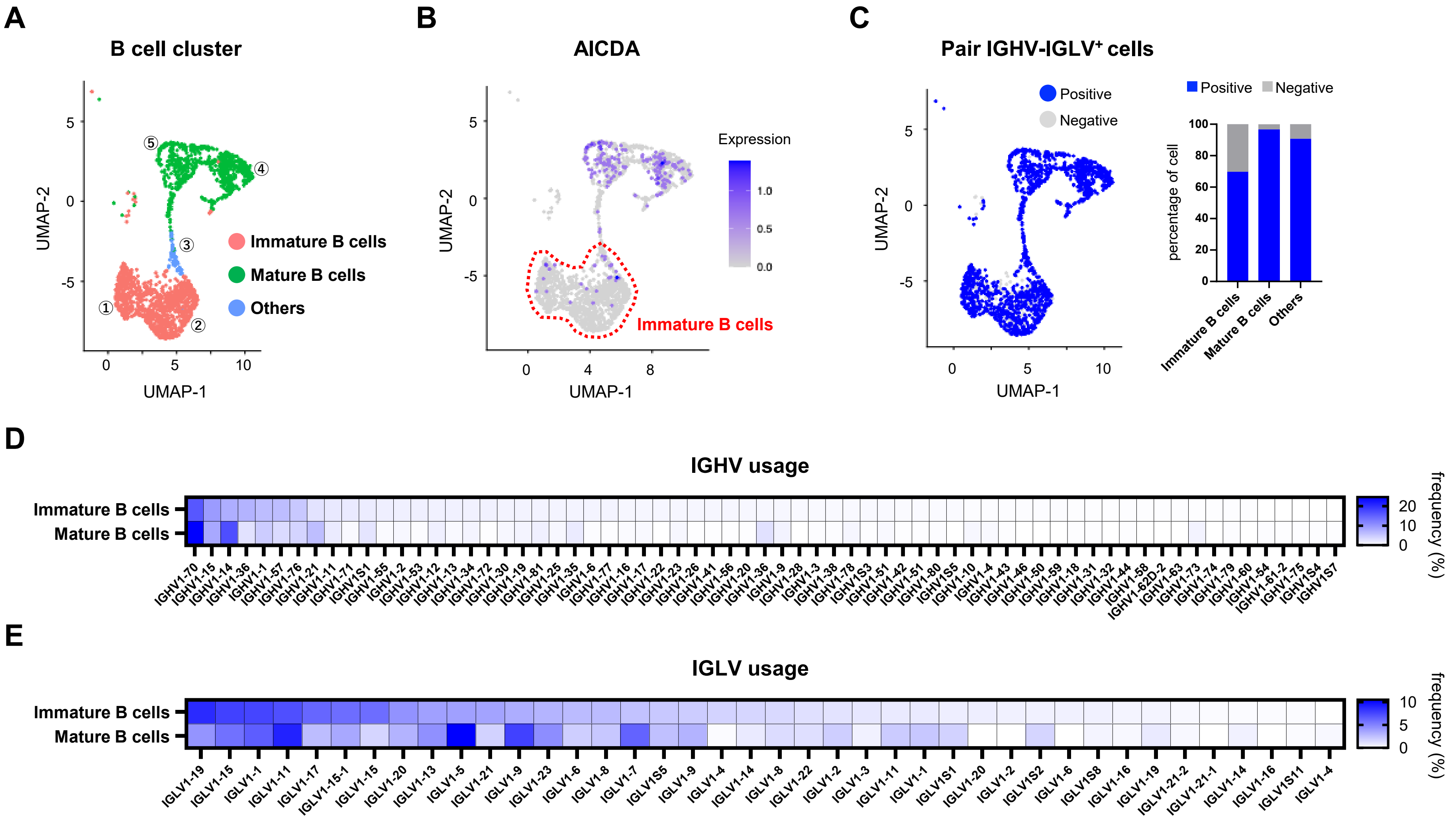
A
B cell cluster
UMAP-2
UMAP-1
Immature B cells
Mature B cells
Others
①
②
③
④
⑤
B
AICDA
UMAP-2
UMAP-1
Expression
1.0
0.5
0.0
Immature B cells
C
Pair IGHV-IGLV⁺ cells
UMAP-2
UMAP-1
Positive
Negative
percentage of cell
Immature B cells
Mature B cells
Others
D
IGHV usage
Immature B cells
Mature B cells
frequency (%)
IGHV1-70
IGHV1-15
IGHV1-14
IGHV1-36
IGHV1-1
IGHV1-57
IGHV1-76
IGHV1-21
IGHV1-11
IGHV1-71
IGHV1S1
IGHV1-55
IGHV1-2
IGHV1-53
IGHV1-12
IGHV1-13
IGHV1-34
IGHV1-72
IGHV1-30
IGHV1-19
IGHV1-81
IGHV1-25
IGHV1-35
IGHV1-6
IGHV1-77
IGHV1-16
IGHV1-17
IGHV1-22
IGHV1-23
IGHV1-26
IGHV1-41
IGHV1-56
IGHV1-20
IGHV1-36
IGHV1-9
IGHV1-28
IGHV1-3
IGHV1-38
IGHV1-78
IGHV1S3
IGHV1-51
IGHV1-42
IGHV1-51
IGHV1-80
IGHV1S5
IGHV1-10
IGHV1-4
IGHV1-43
IGHV1-46
IGHV1-50
IGHV1-59
IGHV1-18
IGHV1-31
IGHV1-32
IGHV1-44
IGHV1-58
IGHV1-62D-2
IGHV1-63
IGHV1-73
IGHV1-74
IGHV1-79
IGHV1-60
IGHV1-54
IGHV1-61-2
IGHV1-75
IGHV1S4
IGHV1S7
E
IGLV usage
Immature B cells
Mature B cells
frequency (%)
IGLV1-19
IGLV1-15
IGLV1-1
IGLV1-11
IGLV1-17
IGLV1-15-1
IGLV1-15
IGLV1-20
IGLV1-13
IGLV1-5
IGLV1-21
IGLV1-9
IGLV1-23
IGLV1-6
IGLV1-8
IGLV1-7
IGLV1S5
IGLV1-9
IGLV1-4
IGLV1-14
IGLV1-8
IGLV1-22
IGLV1-2
IGLV1-3
IGLV1-11
IGLV1-1
IGLV1S1
IGLV1-20
IGLV1-2
IGLV1S2
IGLV1-6
IGLV1S8
IGLV1-16
IGLV1-19
IGLV1-21-2
IGLV1-21-1
IGLV1-14
IGLV1-16
IGLV1S11
IGLV1-4

Figure S6

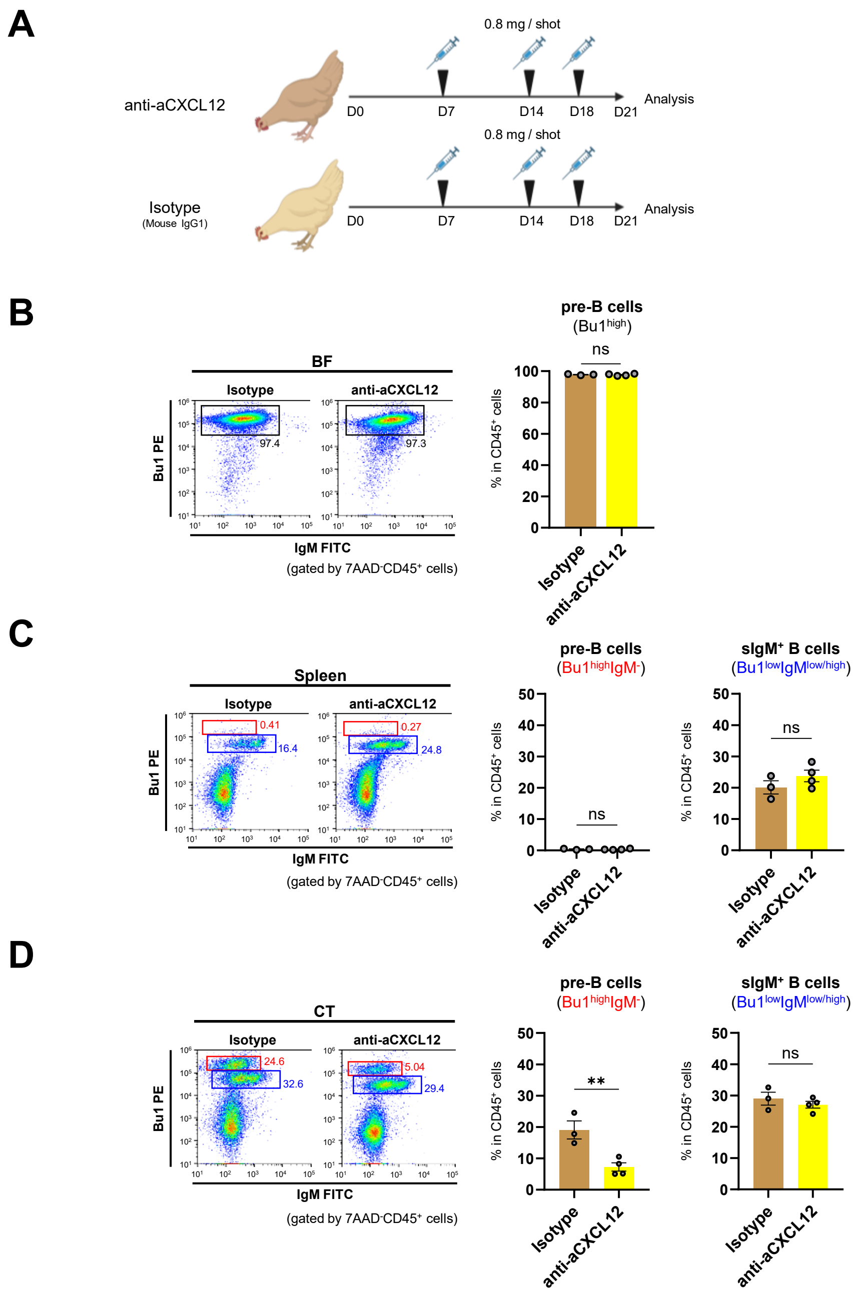

A
anti-aCXCL12
0.8 mg / shot
D0
D7
D14
D18
D21
Analysis
Isotype
(Mouse IgG1)
B
BF
Isotype
anti-aCXCL12
Bu1 PE
IgM FITC
97.4
97.3
(gated by 7AAD-CD45+ cells)
pre-B cells
(Bu1high)
ns
% in CD45+ cells
C
Spleen
0.41
16.4
0.27
24.8
pre-B cells
(Bu1highIgM-)
sIgM+ B cells
(Bu1lowIgMlow/high)
D
CT
24.6
32.6
5.04
29.4
**

Figure S7

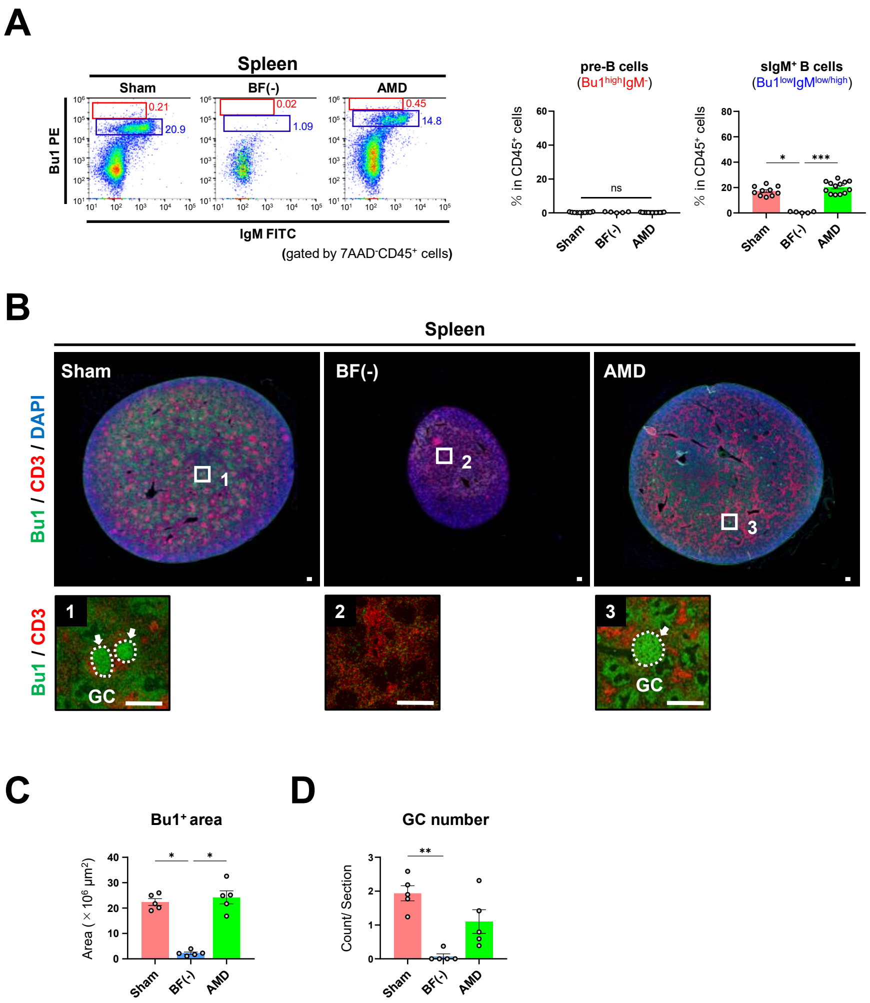

A
Spleen
Sham
BF(-)
AMD
0.21
20.9
0.02
1.09
0.45
14.8
Bu1 PE
IgM FITC
(gated by 7AAD-CD45+ cells)
pre-B cells
(Bu1highIgM-)
sIgM+ B cells
(Bu1lowIgMlow/high)
% in CD45+ cells
ns
*
***
B
Spleen
Sham
BF(-)
AMD
Bu1 / CD3 / DAPI
Bu1 / CD3
1
2
3
GC
C
Bu1+ area
Area (× 10^6 μm^2)
D
GC number
Count/ Section
**

Figure S8

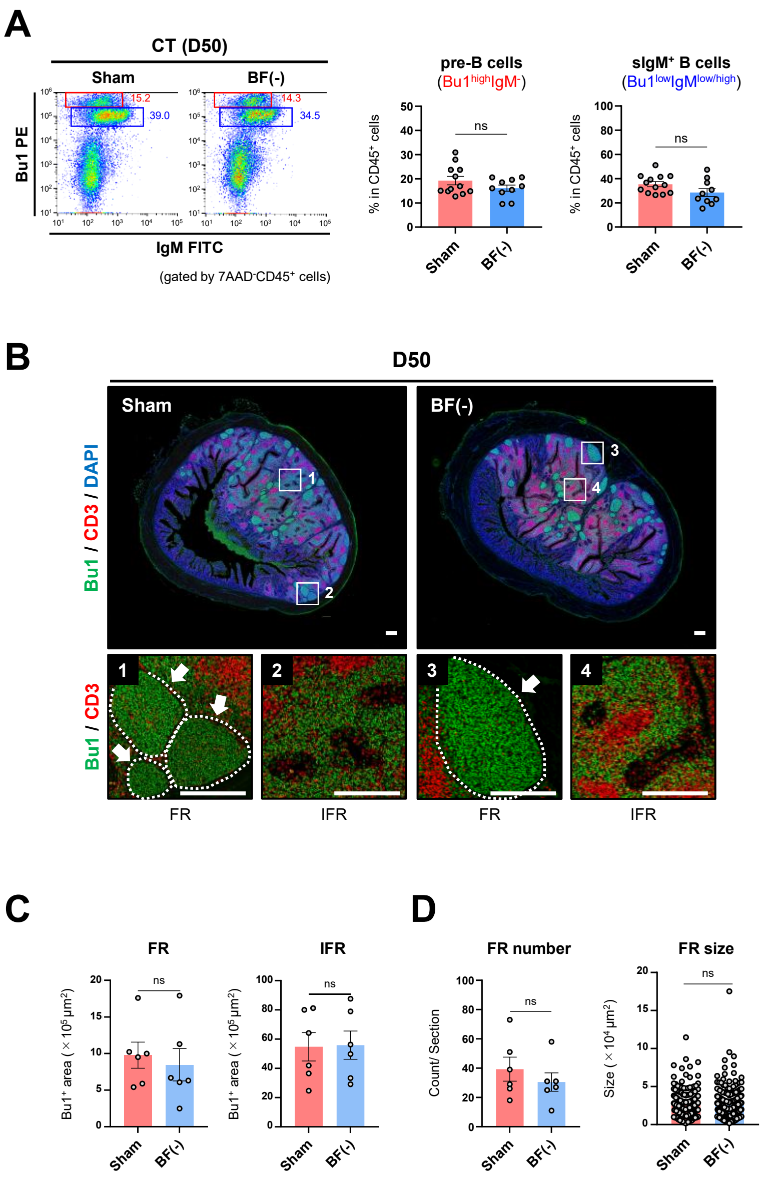

A
CT (D50)
Sham
BF(-)
Bu1 PE
IgM FITC
(gated by 7AAD-CD45+ cells)
pre-B cells
(Bu1highIgM-)
sIgM+ B cells
(Bu1lowIgMlow/high)
% in CD45+ cells
ns
Sham
BF(-)
B
D50
Sham
BF(-)
Bu1 / CD3 / DAPI
Bu1 / CD3
FR
IFR
FR
IFR
C
FR
IFR
Bu1+ area (×10^5 µm^2)
D
FR number
FR size
Count/ Section
Size (×10^4 µm^2)

Figure S9

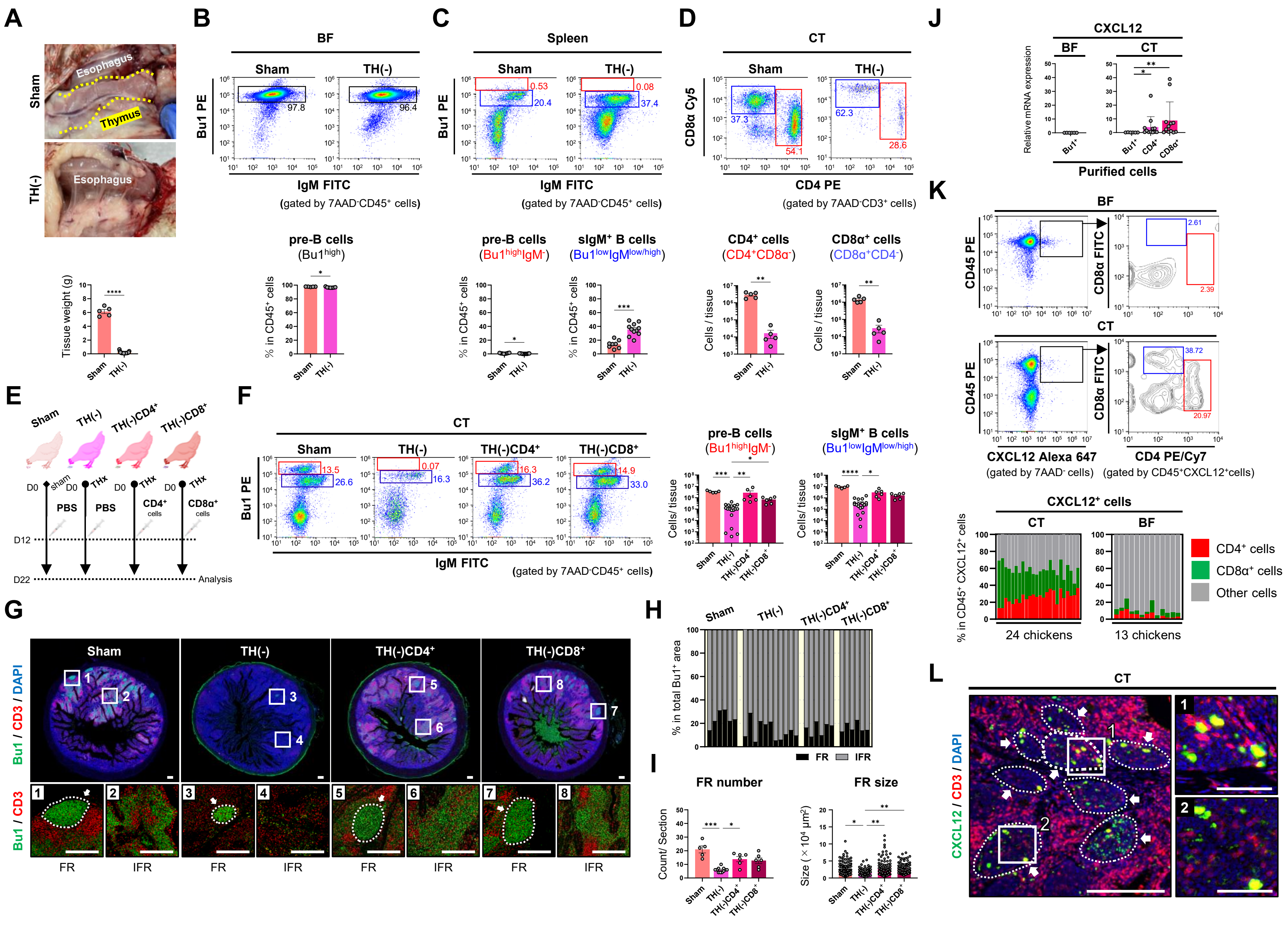

A
Sham
TH(-)
Esophagus
Thymus
Tissue weight (g)
B
BF
Sham
TH(-)
Bu1 PE
IgM FITC
(gated by 7AAD-CD45+ cells)
pre-B cells
(Bu1high)
% in CD45+ cells
C
Spleen
pre-B cells
(Bu1highIgM-)
sIgM+ B cells
(Bu1lowIgMlow/high)
D
CT
CD8α Cy5
CD4 PE
(gated by 7AAD-CD3+ cells)
CD4+ cells
(CD4+CD8α-)
CD8α+ cells
(CD8α+CD4-)
Cells / tissue
E
Sham
TH(-)
TH(-)CD4+
TH(-)CD8+
D0
D12
D22
PBS
CD4+ cells
CD8α+ cells
Analysis
F
CT
Cells/ tissue
G
Bu1 / CD3 / DAPI
Bu1 / CD3
FR
IFR
H
% in total Bu1+ area
FR
IFR
I
FR number
FR size
Count/ Section
Size (×10⁴ μm²)
J
CXCL12
Relative mRNA expression
Purified cells
K
CD45 PE
CD8α FITC
CXCL12 Alexa 647
CD4 PE/Cy7
(gated by 7AAD- cells)
(gated by CD45+CXCL12+cells)
CXCL12+ cells
% in CD45+ CXCL12+ cells
24 chickens
13 chickens
CD4+ cells
CD8α+ cells
Other cells
L
CXCL12 / CD3 / DAPI

Figure S10

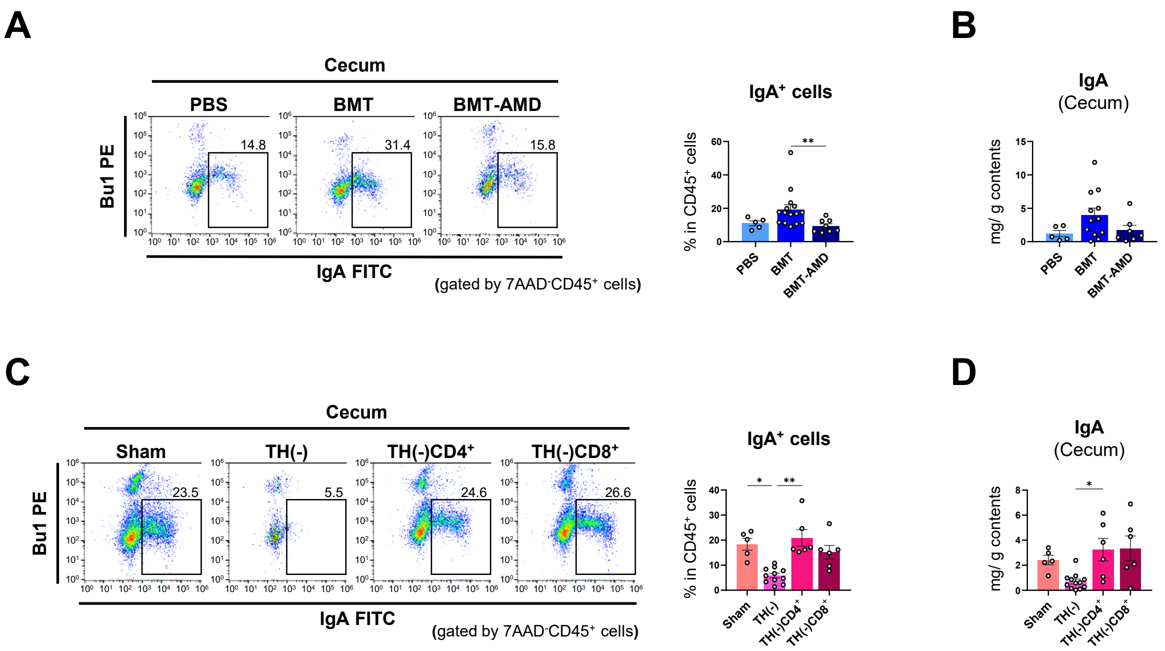

A
Cecum
PBS
BMT
BMT-AMD
14.8
31.4
15.8
Bu1 PE
IgA FITC
(gated by 7AAD-CD45+ cells)
IgA+ cells
% in CD45+ cells
**
PBS
BMT
BMT-AMD
B
IgA
(Cecum)
mg/ g contents
C
Cecum
Sham
TH(-)
TH(-)CD4+
TH(-)CD8+
23.5
5.5
24.6
26.6
Bu1 PE
IgA FITC
(gated by 7AAD-CD45+ cells)
IgA+ cells
% in CD45+ cells
D
IgA
(Cecum)
mg/ g contents

Figure S11

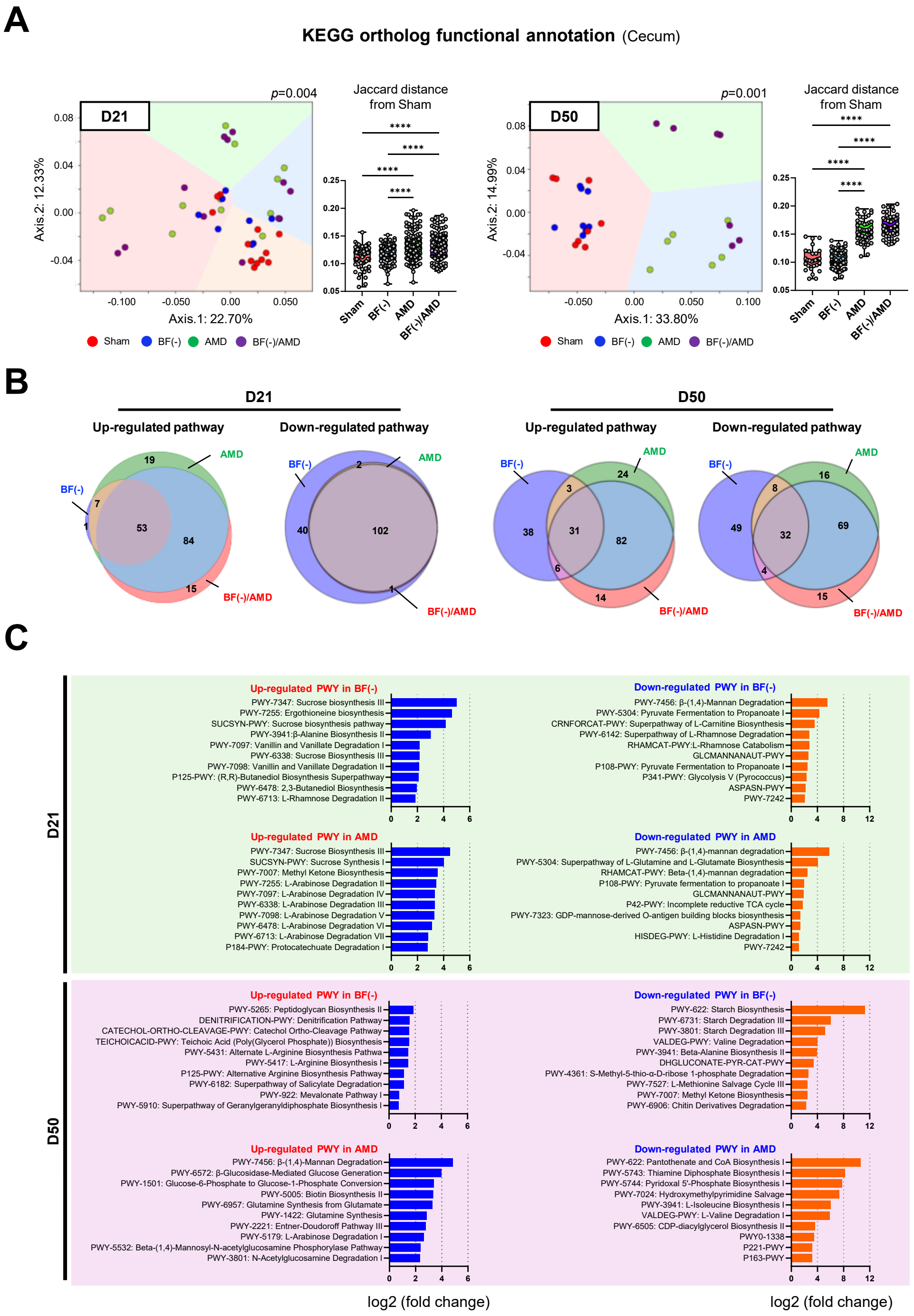
A
KEGG ortholog functional annotation (Cecum)
D21
p=0.004
Axis.2: 12.33%
Axis.1: 22.70%
Jaccard distance from Sham
Sham BF(-) AMD BF(-)/AMD
D50
p=0.001
Axis.2: 14.99%
Axis.1: 33.80%
Jaccard distance from Sham
Sham BF(-) AMD BF(-)/AMD
B
D21
Up-regulated pathway
Down-regulated pathway
D50
Up-regulated pathway
Down-regulated pathway
BF(-)
AMD
BF(-)/AMD
C
D21
D50
Up-regulated PWY in BF(-)
PWY-7347: Sucrose biosynthesis III
PWY-7255: Ergothioneine biosynthesis
SUCSYN-PWY: Sucrose biosynthesis pathway
PWY-3941:β-Alanine Biosynthesis II
PWY-7097: Vanillin and Vanillate Degradation I
PWY-6338: Sucrose Biosynthesis III
PWY-7098: Vanillin and Vanillate Degradation II
P125-PWY: (R,R)-Butanediol Biosynthesis Superpathway
PWY-6478: 2,3-Butanediol Biosynthesis
PWY-6713: L-Rhamnose Degradation II
Down-regulated PWY in BF(-)
PWY-7456: β-(1,4)-Mannan Degradation
PWY-5304: Pyruvate Fermentation to Propanoate I
CRNFORCAT-PWY: Superpathway of L-Carnitine Biosynthesis
PWY-6142: Superpathway of L-Rhamnose Degradation
RHAMCAT-PWY:L-Rhamnose Catabolism
GLCMANNANAUT-PWY
P108-PWY: Pyruvate Fermentation to Propanoate I
P341-PWY: Glycolysis V (Pyrococcus)
ASPASN-PWY
PWY-7242
Up-regulated PWY in AMD
PWY-7347: Sucrose Biosynthesis III
SUCSYN-PWY: Sucrose Synthesis I
PWY-7007: Methyl Ketone Biosynthesis
PWY-7255: L-Arabinose Degradation II
PWY-7097: L-Arabinose Degradation IV
PWY-6338: L-Arabinose Degradation III
PWY-7098: L-Arabinose Degradation V
PWY-6478: L-Arabinose Degradation VI
PWY-6713: L-Arabinose Degradation VII
P184-PWY: Protocatechuate Degradation I
Down-regulated PWY in AMD
PWY-7456: β-(1,4)-mannan degradation
PWY-5304: Superpathway of L-Glutamine and L-Glutamate Biosynthesis
RHAMCAT-PWY: Beta-(1,4)-mannan degradation
P108-PWY: Pyruvate fermentation to propanoate I
GLCMANNANAUT-PWY
P42-PWY: Incomplete reductive TCA cycle
PWY-7323: GDP-mannose-derived O-antigen building blocks biosynthesis
ASPASN-PWY
HISDEG-PWY: L-Histidine Degradation I
PWY-7242
Up-regulated PWY in BF(-)
PWY-5265: Peptidoglycan Biosynthesis II
DENITRIFICATION-PWY: Denitrification Pathway
CATECHOL-ORTHO-CLEAVAGE-PWY: Catechol Ortho-Cleavage Pathway
TEICHOICACID-PWY: Teichoic Acid (Poly(Glycerol Phosphate)) Biosynthesis
PWY-5431: Alternate L-Arginine Biosynthesis Pathwa
PWY-5417: L-Arginine Biosynthesis I
P125-PWY: Alternative Arginine Biosynthesis Pathway
PWY-6182: Superpathway of Salicylate Degradation
PWY-922: Mevalonate Pathway I
PWY-5910: Superpathway of Geranylgeranyldiphosphate Biosynthesis I
Down-regulated PWY in BF(-)
PWY-622: Starch Biosynthesis
PWY-6731: Starch Degradation III
PWY-3801: Starch Degradation III
VALDEG-PWY: Valine Degradation
PWY-3941: Beta-Alanine Biosynthesis II
DHGLUCONATE-PYR-CAT-PWY
PWY-4361: S-Methyl-5-thio-α-D-ribose 1-phosphate Degradation
PWY-7527: L-Methionine Salvage Cycle III
PWY-7007: Methyl Ketone Biosynthesis
PWY-6906: Chitin Derivatives Degradation
Up-regulated PWY in AMD
PWY-7456: β-(1,4)-Mannan Degradation
PWY-6572: β-Glucosidase-Mediated Glucose Generation
PWY-1501: Glucose-6-Phosphate to Glucose-1-Phosphate Conversion
PWY-5005: Biotin Biosynthesis II
PWY-6957: Glutamine Synthesis from Glutamate
PWY-1422: Glutamine Synthesis
PWY-2221: Entner-Doudoroff Pathway III
PWY-5179: L-Arabinose Degradation I
PWY-5532: Beta-(1,4)-Mannosyl-N-acetylglucosamine Phosphorylase Pathway
PWY-3801: N-Acetylglucosamine Degradation I
Down-regulated PWY in AMD
PWY-622: Pantothenate and CoA Biosynthesis I
PWY-5743: Thiamine Diphosphate Biosynthesis I
PWY-5744: Pyridoxal 5'-Phosphate Biosynthesis I
PWY-7024: Hydroxymethylpyrimidine Salvage
PWY-3941: L-Isoleucine Biosynthesis I
VALDEG-PWY: L-Valine Degradation I
PWY-6505: CDP-diacylglycerol Biosynthesis II
PWY0-1338
P221-PWY
P163-PWY
log2 (fold change)
log2 (fold change)

Figure S12

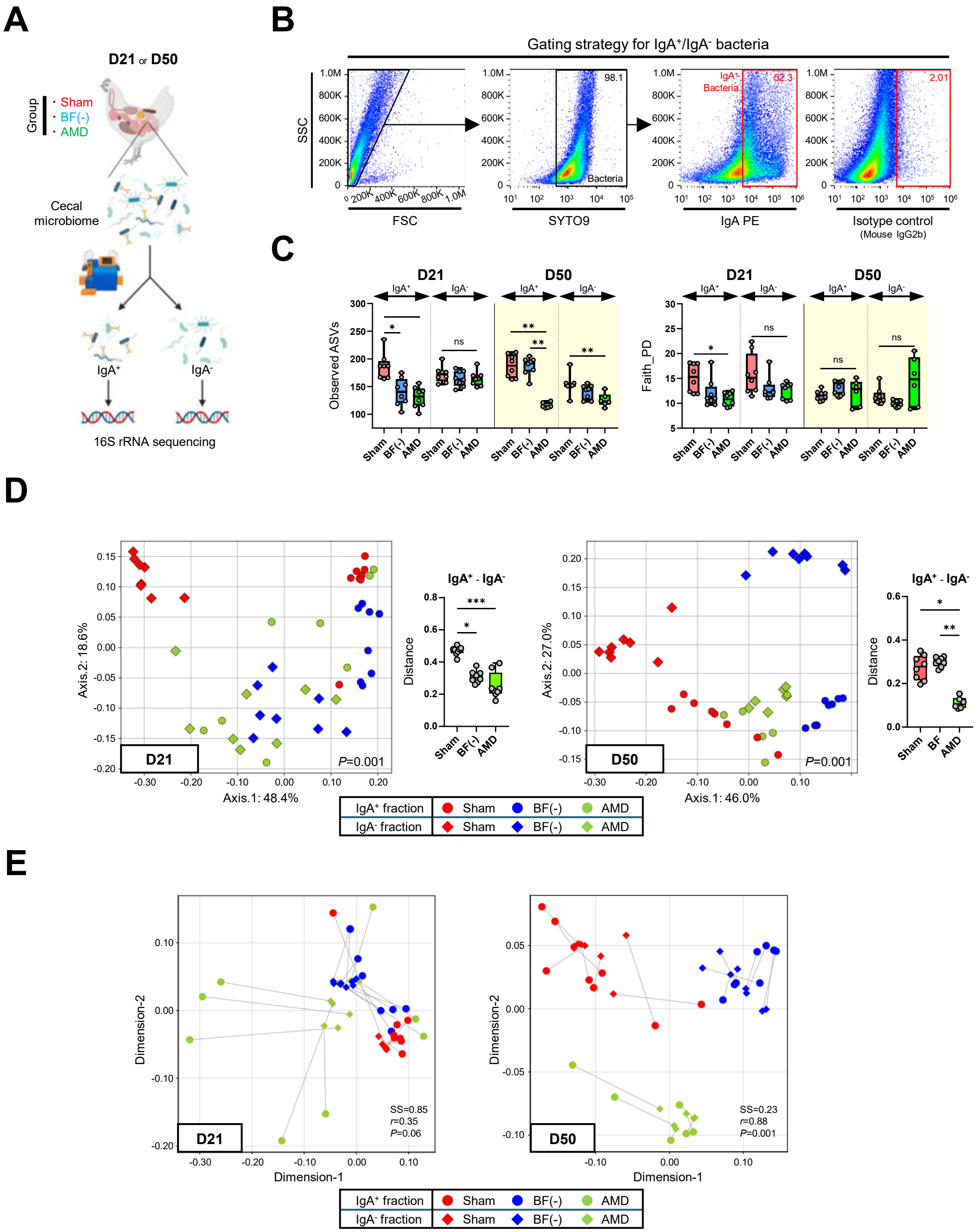
A
D21 or D50
Group
Sham
BF(-)
AMD
Cecal microbiome
IgA+
IgA-
16S rRNA sequencing
B
Gating strategy for IgA+/IgA- bacteria
SSC
FSC
SYTO9
Bacteria
98.1
IgA+ Bacteria
52.3
IgA PE
2.01
Isotype control
(Mouse IgG2b)
C
D21
D50
Observed ASVs
Faith_PD
Sham
BF(-)
AMD
ns
D
IgA+ - IgA-
Distance
Axis.1: 48.4%
Axis.2: 18.6%
P=0.001
Axis.1: 46.0%
Axis.2: 27.0%
IgA+ fraction
IgA- fraction
E
Dimension-1
Dimension-2
SS=0.85
r=0.35
P=0.06
SS=0.23
r=0.88
P=0.001

Figure S13

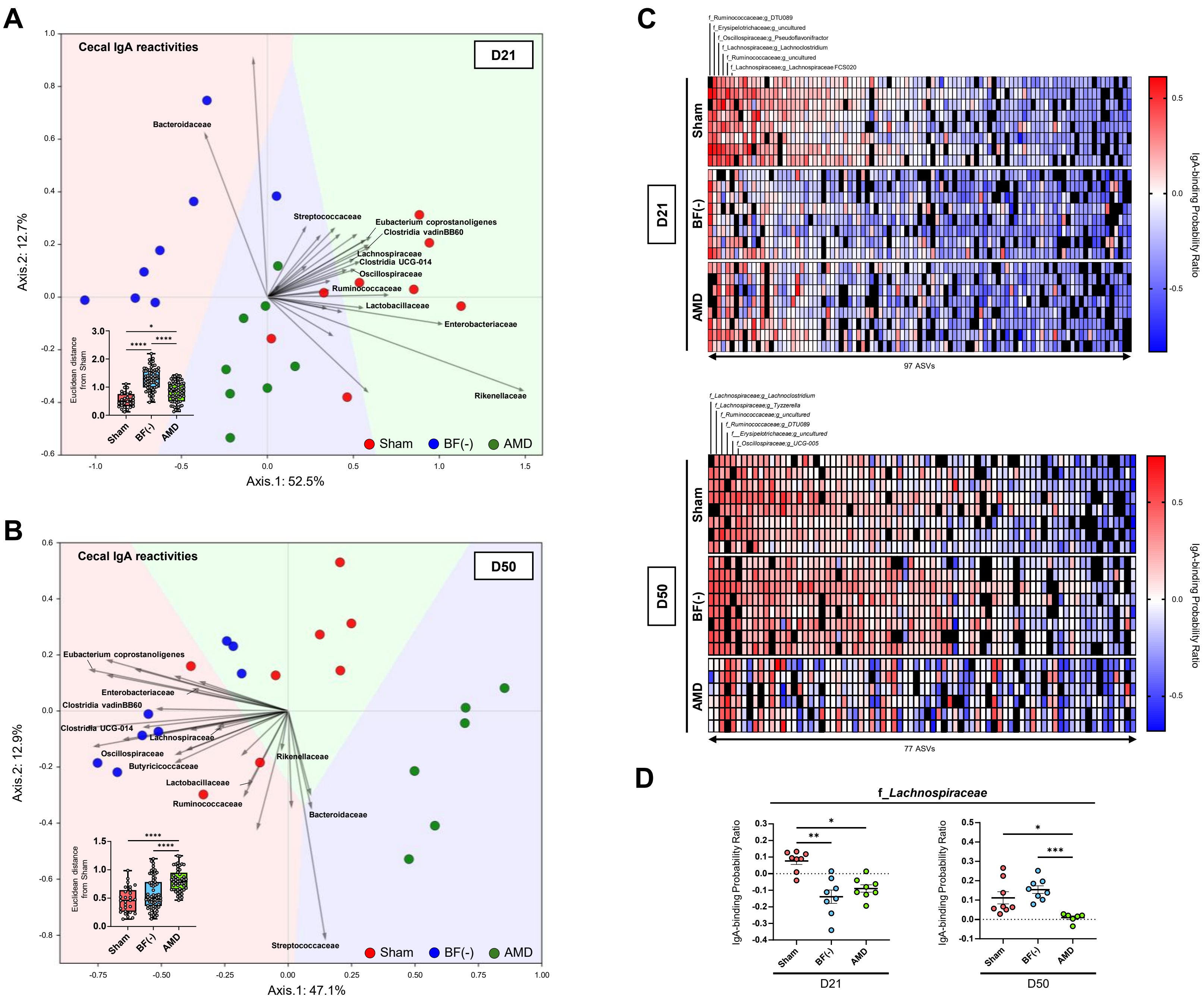
A
Cecal IgA reactivities
D21
Bacteroidaceae
Streptococcaceae
Eubacterium coprostanoligenes
Clostridia vadinBB60
Lachnospiraceae
Clostridia UCG-014
Oscillospiraceae
Ruminococcaceae
Lactobacillaceae
Enterobacteriaceae
Rikenellaceae
Axis.2: 12.7%
Axis.1: 52.5%
Euclidean distance from Sham
Sham
BF(-)
AMD
B
Cecal IgA reactivities
D50
Eubacterium coprostanoligenes
Enterobacteriaceae
Clostridia vadinBB60
Clostridia UCG-014
Lachnospiraceae
Oscillospiraceae
Butyricicoccaceae
Lactobacillaceae
Ruminococcaceae
Rikenellaceae
Bacteroidaceae
Streptococcaceae
Axis.2: 12.9%
Axis.1: 47.1%
C
f_Ruminococcaceae;g_DTU089
f_Erysipelotrichaceae;g_uncultured
f_Oscillospiraceae;g_Pseudoflavonifractor
f_Lachnospiraceae;g_Lachnoclostridium
f_Ruminococcaceae;g_uncultured
f_Lachnospiraceae;g_Lachnospiraceae FCS020
D21
97 ASVs
IgA-binding Probability Ratio
f_Lachnospiraceae;g_Lachnoclostridium
f_Lachnospiraceae;g_Tyzzerella
f_Ruminococcaceae;g_uncultured
f_Ruminococcaceae;g_DTU089
f__Erysipelotrichaceae;g_uncultured
f_Oscillospiraceae;g_UCG-005
D50
77 ASVs
D
f_Lachnospiraceae
IgA-binding Probability Ratio
D21
D50

Figure S14

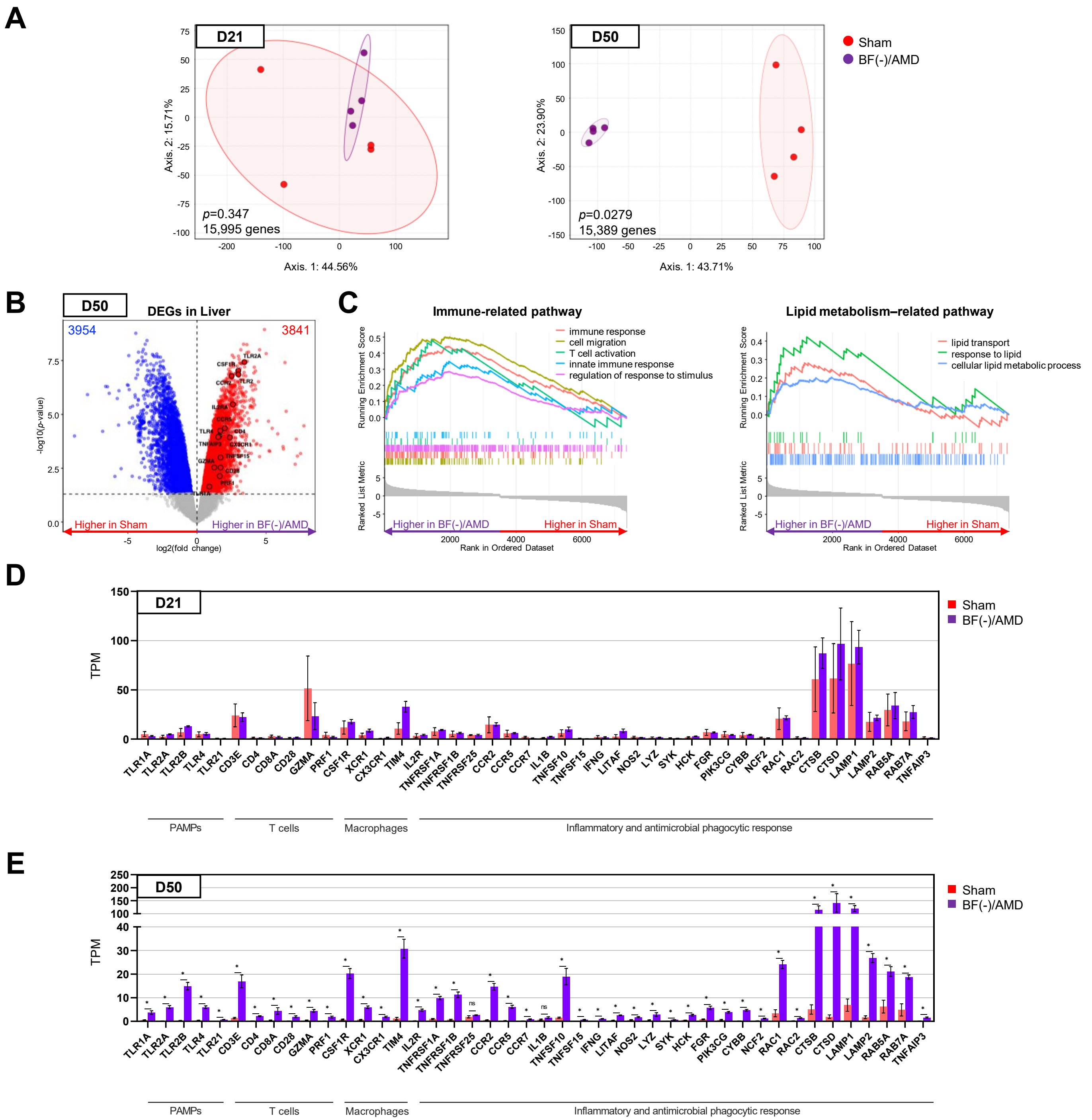
A
D21
p=0.347
15,995 genes
Axis. 1: 44.56%
Axis. 2: 15.71%
D50
p=0.0279
15,389 genes
Axis. 1: 43.71%
Axis. 2: 23.90%
Sham
BF(-)/AMD
B
D50
DEGs in Liver
3954
3841
Higher in Sham
Higher in BF(-)/AMD
log2(fold change)
-log10(p-value)
C
Immune-related pathway
immune response
cell migration
T cell activation
innate immune response
regulation of response to stimulus
Lipid metabolism–related pathway
lipid transport
response to lipid
cellular lipid metabolic process
Running Enrichment Score
Ranked List Metric
Rank in Ordered Dataset
Higher in BF(-)/AMD
Higher in Sham
D
D21
TPM
Sham
BF(-)/AMD
TLR1A TLR2A TLR2B TLR4 TLR21 CD3E CD4 CD8A CD28 GZMA PRF1 CSF1R XCR1 CX3CR1 TIM4 IL2R TNFRSF1A TNFRSF1B TNFRSF25 CCR2 CCR5 CCR7 IL1B TNFSF10 TNFSF15 IFNG LITAF NOS2 LYZ SYK HCK FGR PIK3CG CYBB NCF2 RAC1 RAC2 CTSB CTSD LAMP1 LAMP2 RAB5A RAB7A TNFAIP3
PAMPs
T cells
Macrophages
Inflammatory and antimicrobial phagocytic response
E
D50
TPM
Sham
BF(-)/AMD
PAMPs
T cells
Macrophages
Inflammatory and antimicrobial phagocytic response

Figure S15

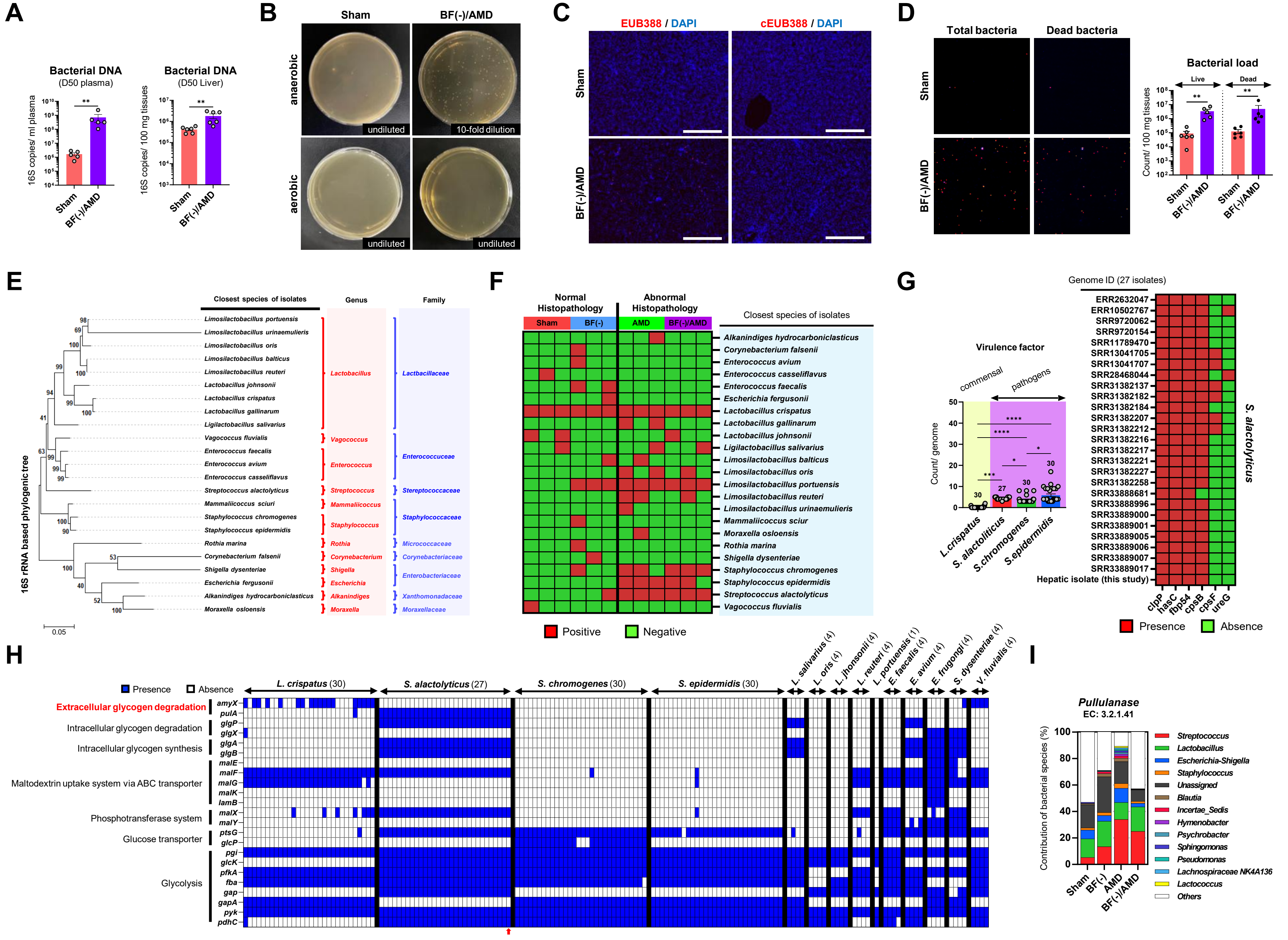
A
Bacterial DNA (D50 plasma)
16S copies/ ml plasma
Bacterial DNA (D50 Liver)
16S copies/ 100 mg tissues
Sham
BF(-)/AMD
B
Sham
BF(-)/AMD
anaerobic
aerobic
undiluted
10-fold dilution
C
EUB388 / DAPI
cEUB388 / DAPI
Sham
BF(-)/AMD
D
Total bacteria
Dead bacteria
Bacterial load
Live
Dead
Count/ 100 mg tissues
E
Closest species of isolates
Genus
Family
16S rRNA based phylogenic tree
Limosilactobacillus portuensis
Limosilactobacillus urinaemulieris
Limosilactobacillus oris
Limosilactobacillus balticus
Limosilactobacillus reuteri
Lactobacillus johnsonii
Lactobacillus crispatus
Lactobacillus gallinarum
Ligilactobacillus salivarius
Vagococcus fluvialis
Enterococcus faecalis
Enterococcus avium
Enterococcus casseliflavus
Streptococcus alactolyticus
Mammaliicoccus sciuri
Staphylococcus chromogenes
Staphylococcus epidermidis
Rothia marina
Corynebacterium falsenii
Shigella dysenteriae
Escherichia fergusonii
Alkanindiges hydrocarbonoclasticus
Moraxella osloensis
Lactobacillus
Vagococcus
Enterococcus
Streptococcus
Mammaliicoccus
Staphylococcus
Rothia
Corynebacterium
Shigella
Escherichia
Alkanindiges
Moraxella
Lactbacillaceae
Enterococcuceae
Stereptococcaceae
Staphylococcaceae
Micrococcaceae
Corynebacteriaceae
Enterobacteriaceae
Xanthomonadaceae
Moraxellaceae
0.05
F
Normal Histopathology
Abnormal Histopathology
Sham
BF(-)
AMD
BF(-)/AMD
Closest species of isolates
Alkanindiges hydrocarboniclasticus
Corynebacterium falsenii
Enterococcus avium
Enterococcus casseliflavus
Enterococcus faecalis
Escherichia fergusonii
Lactobacillus crispatus
Lactobacillus gallinarum
Lactobacillus johnsonii
Ligilactobacillus salivarius
Limosilactobacillus balticus
Limosilactobacillus oris
Limosilactobacillus portuensis
Limosilactobacillus reuteri
Limosilactobacillus urinaemulieris
Mammaliicoccus sciur
Moraxella osloensis
Rothia marina
Shigella dysenteriae
Staphylococcus chromogenes
Staphylococcus epidermidis
Streptococcus alactolyticus
Vagococcus fluvialis
Positive
Negative
G
Virulence factor
commensal
pathogens
Count/ genome
L.crispatus
S. alactoliticus
S.chromogenes
S.epidermidis
Genome ID (27 isolates)
ERR2632047
ERR10502767
SRR9720062
SRR9720154
SRR11789470
SRR13041705
SRR13041707
SRR28468044
SRR31382137
SRR31382182
SRR31382184
SRR31382207
SRR31382212
SRR31382216
SRR31382217
SRR31382221
SRR31382227
SRR31382258
SRR33888681
SRR33888996
SRR33889000
SRR33889001
SRR33889005
SRR33889006
SRR33889007
SRR33889017
Hepatic isolate (this study)
clpP
hasC
fbp54
cpsB
cpsF
ureG
S. alactolyticus
Presence
Absence
H
Presence
Absence
L. crispatus (30)
S. alactolyticus (27)
S. chromogenes (30)
S. epidermidis (30)
L. salivarius (4)
L. oris (4)
L. jhonsonii (4)
L. reuteri (4)
L. portuensis (1)
E. faecalis (4)
E. avium (4)
E. frugongi (4)
S. dysenteriae (4)
V. fluvialis (4)
Extracellular glycogen degradation
Intracellular glycogen degradation
Intracellular glycogen synthesis
Maltodextrin uptake system via ABC transporter
Phosphotransferase system
Glucose transporter
Glycolysis
amyX
pulA
glgP
glgX
glgA
glgB
malE
malF
malG
malK
lamB
malX
malY
ptsG
glcP
pgi
glcK
pfkA
fba
gap
gapA
pyk
pdhC
I
Pullulanase
EC: 3.2.1.41
Contribution of bacterial species (%)
Sham
BF(-)
AMD
BF(-)/AMD
Streptococcus
Lactobacillus
Escherichia-Shigella
Staphylococcus
Unassigned
Blautia
Incertae_Sedis
Hymenobacter
Psychrobacter
Sphingomonas
Pseudomonas
Lachnospiraceae NK4A136
Lactococcus
Others

Figure S16

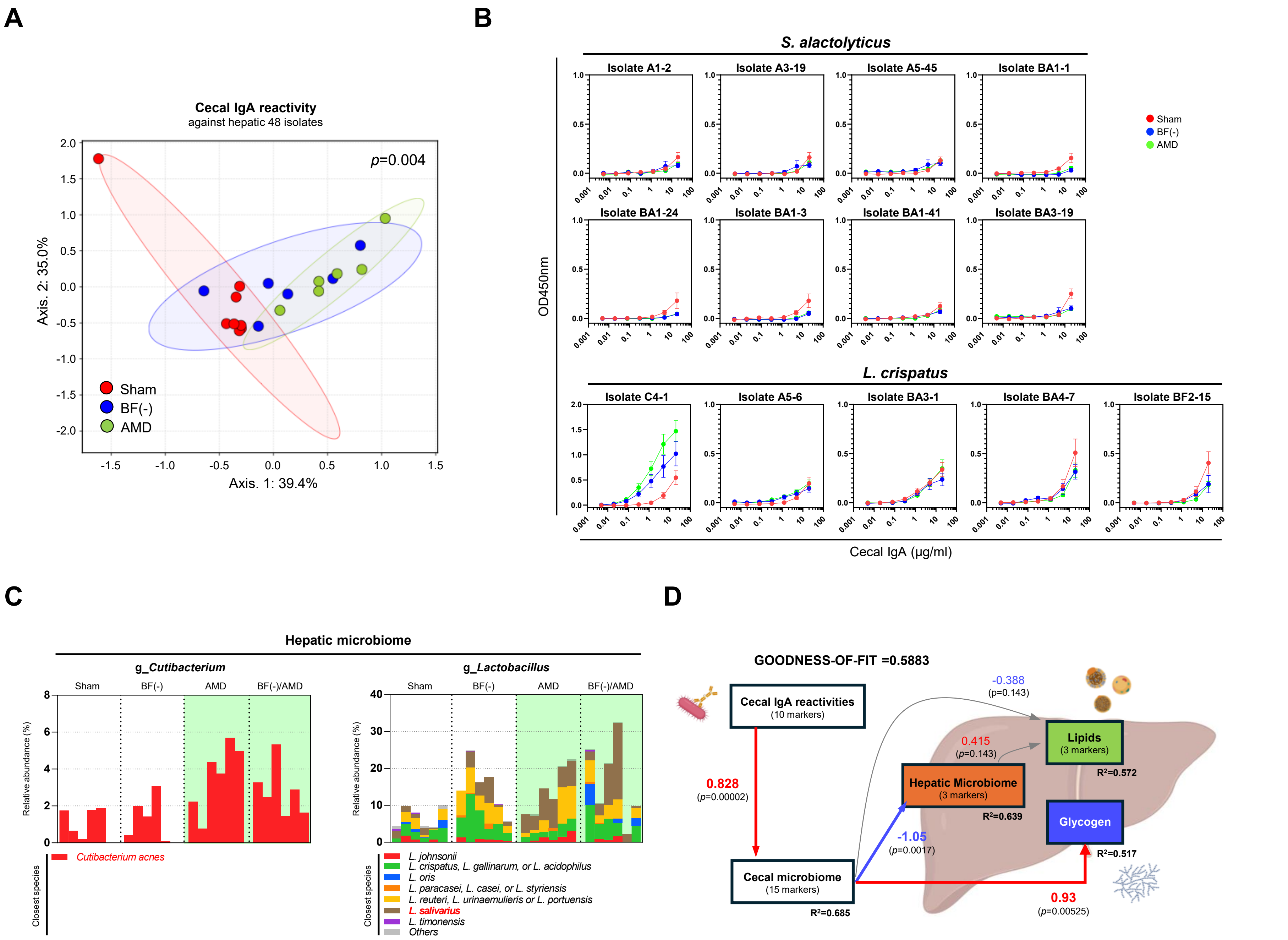
A
Cecal IgA reactivity
against hepatic 48 isolates
p=0.004
Axis. 2: 35.0%
Axis. 1: 39.4%
Sham
BF(-)
AMD
B
S. alactolyticus
Isolate A1-2
Isolate A3-19
Isolate A5-45
Isolate BA1-1
Isolate BA1-24
Isolate BA1-3
Isolate BA1-41
Isolate BA3-19
L. crispatus
Isolate C4-1
Isolate A5-6
Isolate BA3-1
Isolate BA4-7
Isolate BF2-15
OD450nm
Cecal IgA (μg/ml)
Sham
BF(-)
AMD
C
Hepatic microbiome
g_Cutibacterium
g_Lactobacillus
Sham
BF(-)
AMD
BF(-)/AMD
Relative abundance (%)
Closest species
Cutibacterium acnes
L. johnsonii
L. crispatus, L. gallinarum, or L. acidophilus
L. oris
L. paracasei, L. casei, or L. styriensis
L. reuteri, L. urinaemulieris or L. portuensis
L. salivarius
L. timonensis
Others
D
GOODNESS-OF-FIT =0.5883
Cecal IgA reactivities
(10 markers)
0.828
(p=0.00002)
Cecal microbiome
(15 markers)
R²=0.685
-1.05
(p=0.0017)
Hepatic Microbiome
(3 markers)
R²=0.639
0.415
(p=0.143)
-0.388
(p=0.143)
Lipids
(3 markers)
R²=0.572
Glycogen
R²=0.517
0.93
(p=0.00525)

Figure S17

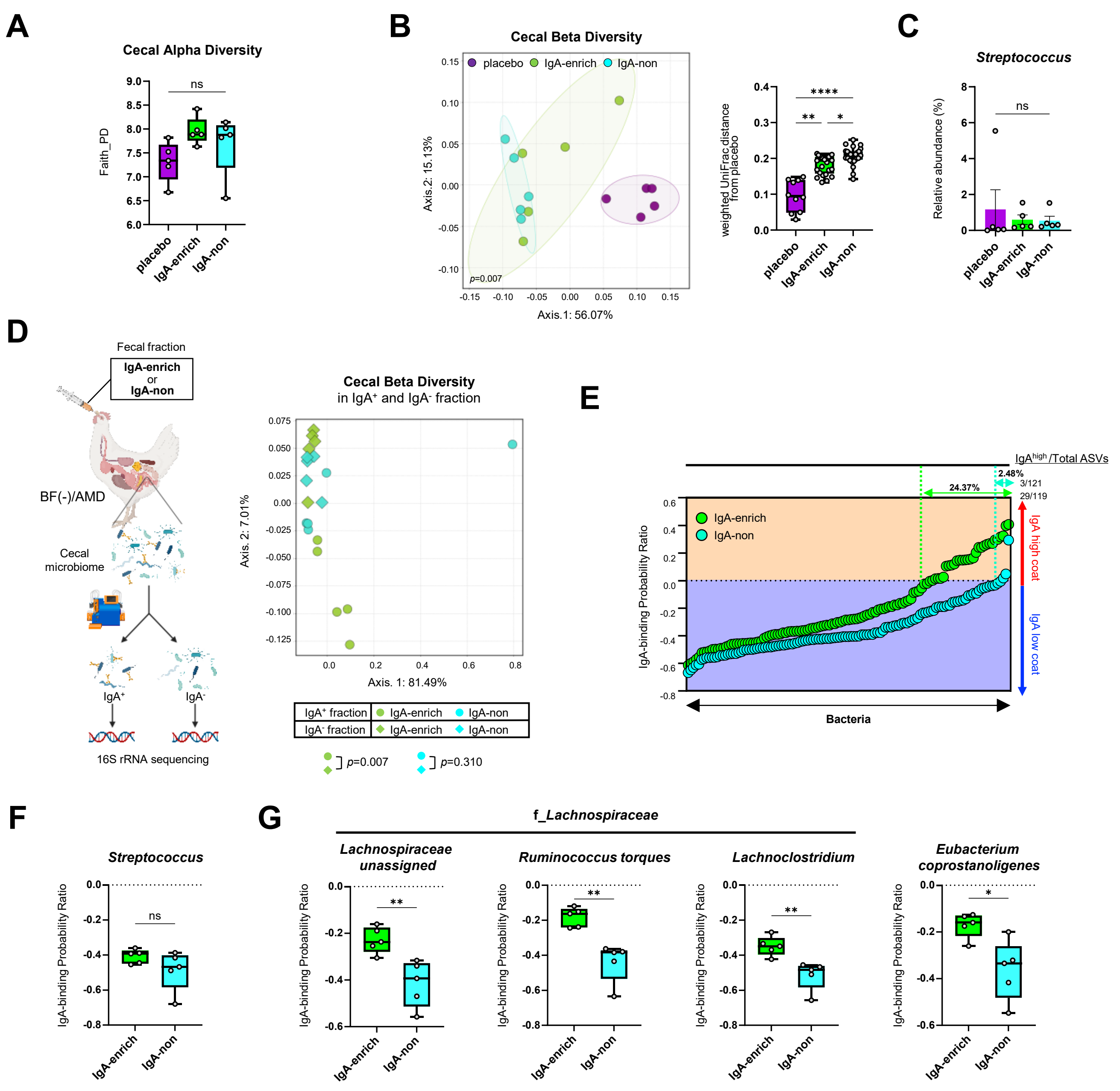
A
Cecal Alpha Diversity
Faith_PD
ns
placebo
IgA-enrich
IgA-non
B
Cecal Beta Diversity
placebo
IgA-enrich
IgA-non
Axis.2: 15.13%
Axis.1: 56.07%
p=0.007
weighted UniFrac distance from placebo
****
**
*
C
Streptococcus
Relative abundance (%)
ns
D
Fecal fraction
IgA-enrich
or
IgA-non
BF(-)/AMD
Cecal microbiome
IgA+
IgA-
16S rRNA sequencing
Cecal Beta Diversity
in IgA+ and IgA- fraction
Axis. 2: 7.01%
Axis. 1: 81.49%
IgA+ fraction
IgA- fraction
p=0.007
p=0.310
E
IgAhigh /Total ASVs
2.48%
3/121
24.37%
29/119
IgA-binding Probability Ratio
IgA high coat
IgA low coat
Bacteria
F
Streptococcus
IgA-binding Probability Ratio
G
f_Lachnospiraceae
Lachnospiraceae unassigned
Ruminococcus torques
Lachnoclostridium
Eubacterium coprostanoligenes
**

Figure S18

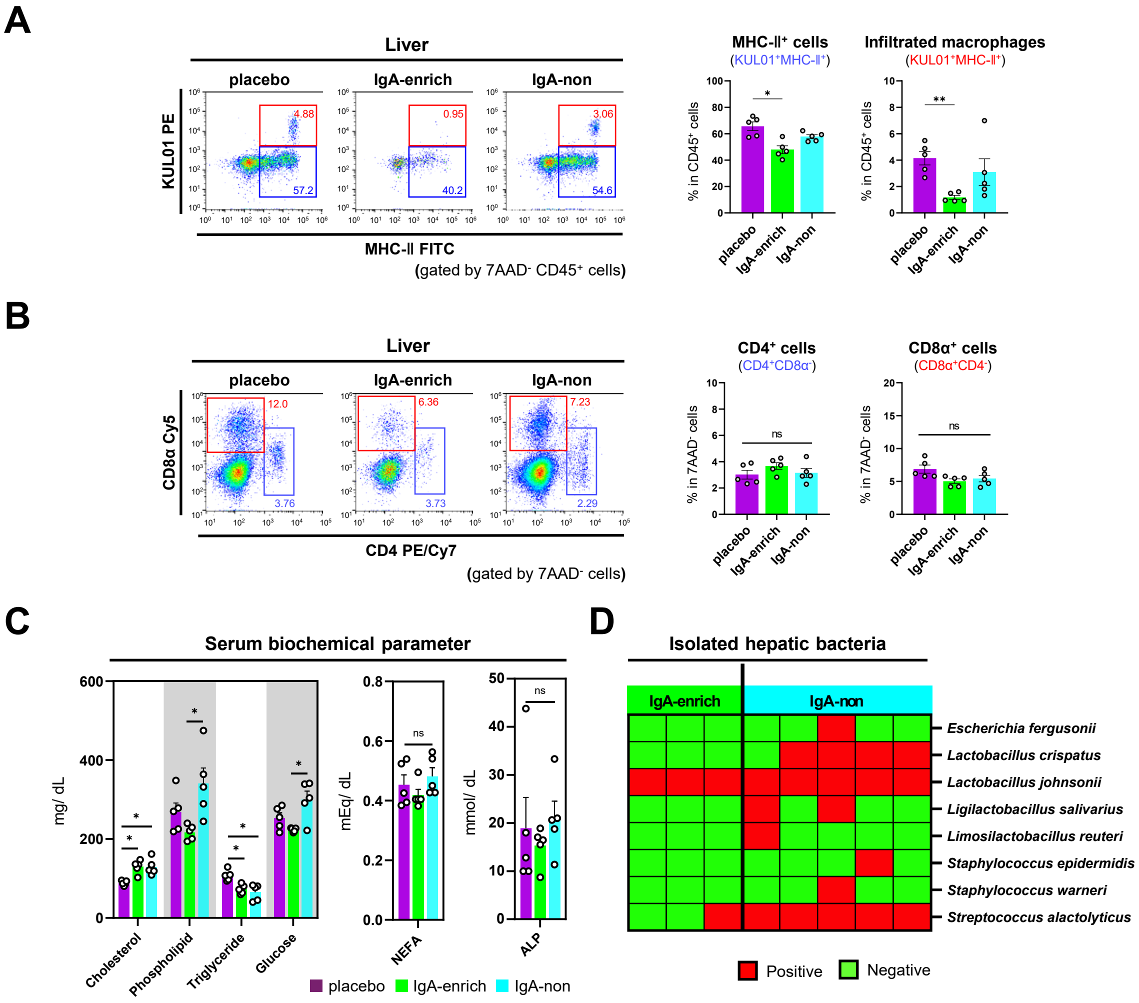
A
Liver
placebo
IgA-enrich
IgA-non
KUL01 PE
MHC-Ⅱ FITC
(gated by 7AAD⁻ CD45⁺ cells)
4.88
57.2
0.95
40.2
3.06
54.6
MHC-Ⅱ⁺ cells
(KUL01⁺MHC-Ⅱ⁺)
Infiltrated macrophages
(KUL01⁺MHC-Ⅱ⁺)
% in CD45⁺ cells
B
CD8α Cy5
CD4 PE/Cy7
(gated by 7AAD⁻ cells)
12.0
3.76
6.36
3.73
7.23
2.29
CD4⁺ cells
(CD4⁺CD8α⁻)
CD8α⁺ cells
(CD8α⁺CD4⁻)
% in 7AAD⁻ cells
ns
C
Serum biochemical parameter
mg/ dL
mEq/ dL
mmol/ dL
Cholesterol
Phospholipid
Triglyceride
Glucose
NEFA
ALP
placebo
IgA-enrich
IgA-non
D
Isolated hepatic bacteria
IgA-enrich
IgA-non
Escherichia fergusonii
Lactobacillus crispatus
Lactobacillus johnsonii
Ligilactobacillus salivarius
Limosilactobacillus reuteri
Staphylococcus epidermidis
Staphylococcus warneri
Streptococcus alactolyticus
Positive
Negative

Figure S19

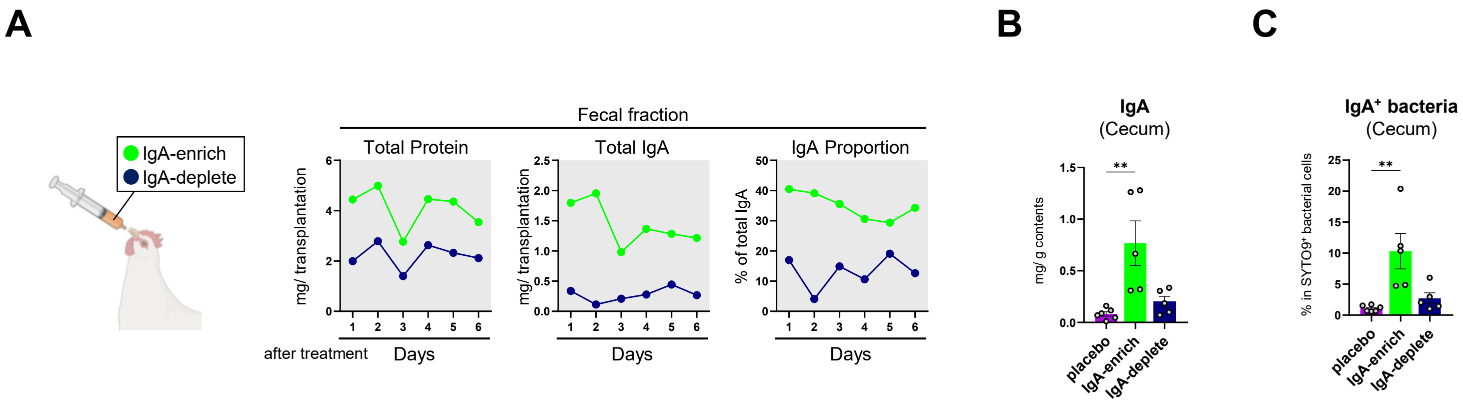
A
B
C
IgA-enrich
IgA-deplete
Fecal fraction
Total Protein
Total IgA
IgA Proportion
mg/ transplantation
% of total IgA
after treatment
Days
IgA
(Cecum)
mg/ g contents
IgA⁺ bacteria
(Cecum)
% in SYTO9⁺ bacterial cells
**
placebo
IgA-enrich
IgA-deplete
